# A male-biased sex-distorter gene drive for the human malaria vector *Anopheles gambiae*

**DOI:** 10.1038/s41587-020-0508-1

**Published:** 2020-05-11

**Authors:** Alekos Simoni, Andrew M. Hammond, Andrea K. Beaghton, Roberto Galizi, Chrysanthi Taxiarchi, Kyros Kyrou, Dario Meacci, Matthew Gribble, Giulia Morselli, Austin Burt, Tony Nolan, Andrea Crisanti

**Affiliations:** 1 Department of Life Sciences, Imperial College London, London, UK; 2 Polo d’Innovazione Genomica, Genetica e Biologia, Terni, Italy; 3 W. Harry Feinstone Department of Molecular Microbiology and Immunology, Johns Hopkins University, Baltimore, MD USA; 4 Centre for Applied Entomology and Parasitology, School of Life Sciences, Keele University, Keele, UK; 5 Department of Life Sciences, Imperial College London, Silwood Park, Ascot, UK; 6 Liverpool School of Tropical Medicine, Liverpool, UK; 7 Department of Molecular Medicine, University of Padova, Padova, Italy

**Keywords:** Molecular engineering, Genetics, Gene regulation

## Abstract

Only female insects transmit diseases such as malaria, dengue and Zika; therefore, control methods that bias the sex ratio of insect offspring have long been sought. Genetic elements such as sex-chromosome drives can distort sex ratios to produce unisex populations that eventually collapse, but the underlying molecular mechanisms are unknown. We report a male-biased sex-distorter gene drive (SDGD) in the human malaria vector *Anopheles gambiae*. We induced super-Mendelian inheritance of the X-chromosome-shredding I-PpoI nuclease by coupling this to a CRISPR-based gene drive inserted into a conserved sequence of the *doublesex* (*dsx*) gene. In modeling of invasion dynamics, SDGD was predicted to have a quicker impact on female mosquito populations than previously developed gene drives targeting female fertility. The SDGD at the *dsx* locus led to a male-only population from a 2.5% starting allelic frequency in 10–14 generations, with population collapse and no selection for resistance. Our results support the use of SDGD for malaria vector control.

## Abstract (web summary)

A sex-distorter gene drive causes population collapse in the malaria mosquito.

## Main

Sex-chromosome drivers are genetic elements that interfere with chromosome segregation during meiosis and are over-represented in progeny^1^. In heterogametic sex, they cause an unbalanced male-to-female ratio among offspring, which can potentially lead to population suppression or extinction. Relatively few sex-chromosome drives have been characterized, most likely because they produce an evolutionary conflict with the rest of the genome that selects for autosomal suppressors or resistant sex chromosomes^2,3^.

Mathematical modeling predicts that a driving sex distorter will spread in a population and, in the absence of resistance, cause eventual collapse^4,5^. Population collapse using natural sex-chromosome drives has been reported in laboratory colonies of *Drosophila*^6,7^. In the field, a population crash of the species *Drosophila neotestacea* was detected in Washington State due to a natural X-chromosome distorter that produced a female-only population^8^. Therefore, sex-distorter drives could conceivably be harnessed for invasive pest or vector control^9,10^.

Although Y drives are less common than X drives, they have been described in *Aedes aegypti* and *Culex pipiens* mosquitoes^11,12^. Y drives are particularly attractive for mosquito vector control because they can progressively reduce the number of females and hence disease transmission as they spread. In addition, Y drives are likely to be more effective than X drives because they can increase at a greater rate the fraction of heterogametic driving individuals^3–5^. Synthetic sex distorters have been generated in *A. gambiae* mosquitoes by using site-specific nucleases such as I-PpoI or CRISPR–Cas9, which cleave conserved repeated sequences in the mosquito ribosomal DNA gene cluster located exclusively on the X chromosome^13,14^. These nucleases, when expressed during spermatozoa development, selectively cleave the X chromosome, thereby favoring the production of Y-bearing gametes and causing a 95% male bias in the progeny^13,14^. However, attempts to convert synthetic sex-ratio distorters into Y-chromosome drives have been unsuccessful so far. In most insect species, including *A. gambiae*, the sex chromosomes are transcriptionally shut down during gametogenesis, a process known as meiotic sex-chromosome inactivation^15,16^, which prevents the transcription of X-shredding nucleases if they are inserted into the Y chromosome (personal observation, A.C. and R.G.).

Recently, a gene drive that targeted the *dsx* gene reached 100% frequency in 7–11 generations and crashed a caged population of 600 mosquitoes without inducing resistance^17^. We hypothesized that it might be possible to circumvent meiotic sex-chromosome inactivation by developing an autosomal male-biased sex distorter and coupling sex-ratio distortion with drive. This could result in a quicker impact on disease transmission and a synergistic effect (robustness) between the sex distorter and gene-drive components. Here we report the design and validation of an SDGD to spread the X-chromosome-shredding I-PpoI endonuclease and produce a male-only insect population.

## Results

### Designing an SDGD

**Fig. 1:**
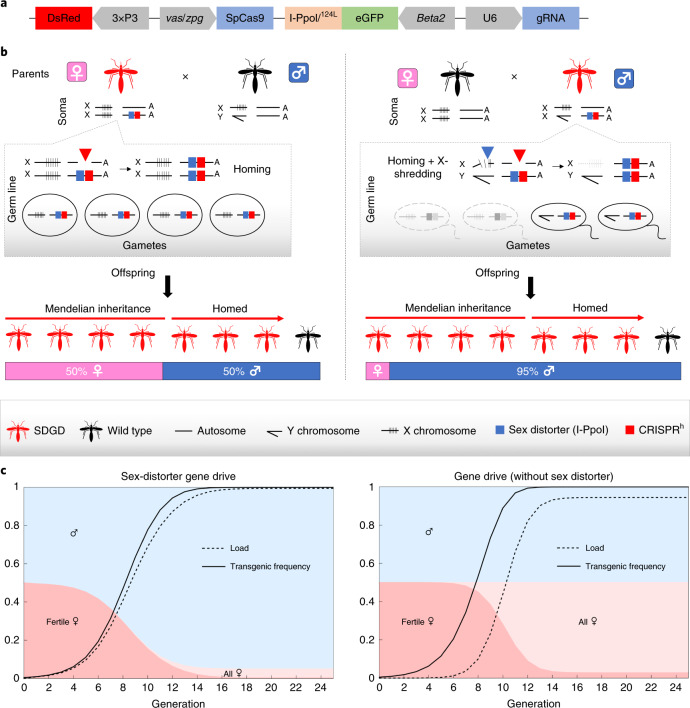
Driving a sex-distorter system in the autosome. **a**, Schematic overview of the construct used to build an SDGD, which contains four transcription units: the I-Ppol nuclease (variant W124L), expressed as a fusion protein with the enhanced green fluorescent protein (eGFP) visual marker, under the control of the male-specific *beta2-tubulin* germline promoter; the SpCas9 nuclease, regulated by a promoter that is active in the germ line of both males and females (from the *vasa* or *zpg* gene); a gRNA under the control of the ubiquitous U6 polymerase III promoter, designed for homing at previously characterized haplosufficient fertility genes; and a 3xP3::DsRed gene as a fluorescent integration marker. **b**, Mode of action of the autosomal SDGD. The sex-distorter (I-Ppol; blue square) and gene-drive (CRISPR^h^; red square) components are linked head to tail in the same construct that is integrated in the autosome within a fertility gene. In the germ line of a female transgenic mosquito (highlighted in red), the CRISPR^h^ component is active (red arrowhead), leading to super-Mendelian inheritance of the transgene by homology-directed repair. In the germ line of a male transgenic mosquito, both the gene-drive (red arrowhead) and sex-distorter (blue arrowhead) transcription units are active, leading to homing of the construct (by action of CRISPR^h^) and shredding of the X chromosome (by action of I-PpoI targeting ribosomal DNA repeats; indicated by vertical lines). This results in a bias of the sex ratio toward males in the progeny and super-Mendelian inheritance of the transgene. **c**, Idealized predictions (discrete-generation deterministic model) of transgenic frequency for spread in a population (solid line) alongside the load on the target population (dashed line) for an SDGD construct (left; fraction of male progeny (*m*) = 0.95) and a gene drive (right; *m* = 0.50) targeting a female fertility gene. The colored shading represents the fraction of males (blue) and females (pink) in the population, with fertile females indicated by a darker color. This idealized model makes several assumptions that are likely to vary by strain, including but not limited to full fitness in males and heterozygous females (fully recessive female fertility gene); complete sterility in homozygous females; 95% SDGD transmission in male and female heterozygotes; no generation of drive-resistant mutations; no loss of function of the sex distorter; and single release of male drive heterozygotes equal to 1% of the male population.

We designed an SDGD system by combining (on the same construct) a CRISPR-based gene drive that targets a haplosufficient female fertility gene with the I-PpoI endonuclease, which in turn cleaves a conserved sequence in the X-linked ribosomal gene cluster (Fig. 1a,b). We used mathematical modeling to test the likely spread of this SDGD design. Our results indicate that our SDGD could spread rapidly from a low starting frequency to produce a largely unisex male population and would also impose a fitness load by impairing female fertility, which together would eliminate the population (Fig. 1c). This SDGD design is different from the previously reported CRISPR-based gene drives that target recessive female fertility genes and impose a fitness load by the generation of homozygous sterile mutants^17,18^. The modeling predicted that this SDGD would quickly bias the population toward males and gradually reduce the abundance of biting females, which both reduce pathogen transmission (by females only) and suppress the population (Table 1 and Supplementary Fig. 1).

**Table 1:** Comparison of performance of gene-drive and sex-distorter genetic control approaches in terms of efficacy, spread and robustness

Construct type	Construct name	Homing rate	Male-biased sex-ratio distortion	Spread in caged population	Population suppression	Development of resistance in cages	Impact of heterozygotes on population size^a^	Component redundancy	Ref.
Gene drive	*dsx*F^CRISPRh^	92% males; 99% females	50%	Yes	Yes	No	No	No	^17^
Sex-distorter gene drive	SDGD^dsx^	92% males; 99% females	93%	Yes	Yes	No	Yes	Yes	This study
Autosomal sex distorter	gfp111A-2	0%	95%	No	Yes (over-flooding ratio of 3×)	No	Yes	No	^13^
Y drive	NA	100% males; 0% females*	95%*	Yes*	NA	NA	Yes*	No	NA

Homing rate is defined as the fraction of transgenic progeny above Mendelian inheritance. An asterisk denotes values based on a hypothetical X-shredder construct inserted on the Y chromosome generating 95% male offspring, all of which inherit the transgene. NA, not applicable. ^a^Ability of the construct to have an impact on the population size (that is, the number of females) in heterozygosity (or hemizygosity for the Y drive) as compared to constructs targeting recessive female fertility loci, which impact population size when homozygote transgenic females are generated.

We generated distinct *A. gambiae* SDGD strains targeting three haplosufficient genes (*AGAP011377*, *AGAP007280* and *AGAP005958*) with established roles in female fertility^18^. We assessed the activity of three SDGD constructs (SDGD^011377^, SDGD^007280^ and SDGD^005958^) in the progeny of crosses between SDGD-heterozygous and wild-type individuals by scoring the fraction of offspring containing the drive element and the sex ratio of the progeny. SDGD^007280^ had severely reduced fertility, and we did not recover enough progeny to assess drive activity. We found average inheritance rates of 79% (±0.17% (s.d.)) for SDGD^011377^ and 98% (±0.08% (s.d.)) for SDGD^005958^ (Supplementary Fig. 2 and Supplementary Table 1). Furthermore, we observed a male bias ranging from 92% to 94% in the progeny of males heterozygous for SDGD^011377^ and SDGD^005958^. Monitoring of life history traits revealed a dramatic reduction of female fertility in females heterozygous for SDGD^011377^ and SDGD^005958^ (Supplementary Fig. 3), similarly to previous findings where in the same genes were targeted with a *vasa*-Cas9 gene-drive construct^18^. We attributed this reduction in fertility to ectopic expression of the *vasa* promoter and subsequent conversion to a null genotype for the target gene in somatic tissues, where the gene product is required^18–20^. In addition, the *vasa* promoter is known to induce maternal deposition of Cas9 into the developing embryo, resulting in deleterious mutations of the paternally inherited gene copy, in addition to the null allele inherited from the mother, imposing additional fitness costs to heterozygous female offspring. We also observed a strong reduction in the fertility of heterozygous males, particularly in SDGD^007280^ and SDGD^005958^ (Supplementary Fig. 3). We hypothesized that male sterility in SDGD^007280^ and partial male sterility in SDGD^005958^ was due to locus-dependent high expression of the I-PpoI nuclease, which, if persisting in spermatozoa, shreds the maternally inherited X chromosome in the fertilized embryo, resulting in embryo lethality^13,21^. Despite high levels of drive transmission and male bias, unintended and severe fertility costs prevented the spread of SDGD^011377^ and SDGD^005958^ into caged mosquito populations when these were seeded at 12.5% allelic frequency (Supplementary Fig. 4). SDGD^005958^ failed to persist in the populations and disappeared after two generations. SDGD^011377^ was stable for eight generations, owing to a better balance of drive and fitness costs. This, in turn, generated low-level population suppression by maintaining a sex ratio of approximately 65% males (Supplementary Fig. 4).

### Optimization of temporal and spatial characteristics and level of expression of Cas9 and I-PpoI

Our initial findings revealed that SDGD constructs targeting female fertility genes could bias both their own inheritance and the sex ratio of progeny. However, fitness costs, most likely associated with non-optimal spatial and temporal activity of both the Cas9 and I-PpoI genes, impaired SDGD spread into mosquito populations. To minimize the ectopic activity of Cas9, we replaced the *vasa* promoter with the regulatory regions of the *zero population growth* (*zpg*) gene (*AGAP006241*). The *zpg* promoter has previously been applied to regulate Cas9 expression in gene-drive constructs, increasing the fertility of heterozygous individuals as compared to those harboring constructs using *vasa*^17,19^. Previous studies have also shown that the expression levels of I-PpoI during spermatogenesis are crucial in determining whether the outcome is sex bias or sterility; high levels of activity correlate with male sterility^13,21^. The destabilized version of I-PpoI (W124L^13^) used in this study was previously found to confer the highest levels of fertility while maintaining strong male bias from at least three independent genomic loci^13^. However, this I-PpoI variant impaired male fertility when expressed under the transgenic *beta2-tubulin* promoter inserted into the *AGAP011377*, *AGAP007280* and *AGAP005958* loci. To reduce the transcriptional activity of the *beta2-tubulin* promoter, we generated three variants by inserting a G+C-rich sequence of 100 bp in proximity to conserved sequences at position −244, −271 or −355 with respect to the ATG start codon (Supplementary Fig. 5). Each variant was tested for expression using a dual-fluorescence reporter system in vivo (Supplementary Fig. 5). For subsequent experiments, we selected *beta2-tubulin* promoter variant 244 (*beta2*^*244*^), which showed transcriptional activity that was about 8.1% that of the wild-type promoter sequence (Supplementary Fig. 6). The initial SDGD plasmid was then modified to replace the *vasa* promoter with the *zpg* regulatory sequences (as described in ref. ^17^), while the *beta2-tubulin* promoter was replaced with *beta2*^*244*^.

### An SDGD targeting the *dsx* gene

**Fig. 2:**
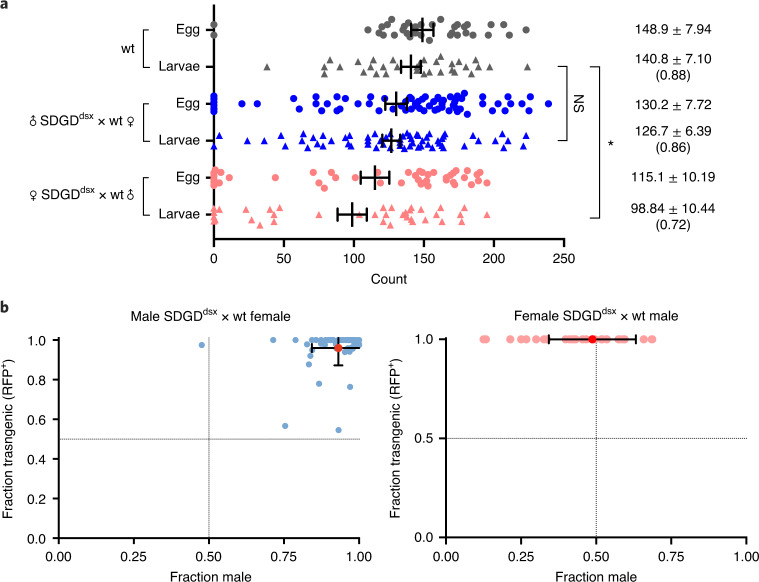
Fertility and sex and inheritance bias of an SDGD targeting the female isoform of the sex-determination *dsx* gene (*AGAP004050*). **a**, Counts of eggs and hatched larvae determined in individual crosses (*n* ≥ 33) of SDGD^dsx^-heterozygous females and males to wild-type (wt) mosquitoes. While male fertility was comparable to that of wild type (male fertility 0.86; no significant difference, NS), females showed a 37% reduction in larval output as compared to wild type (female fertility of 0.627; *P = 0.0124, Kruskal–Wallis test). Values on the right indicate the mean count ± s.e.m., with larval hatching rate in parentheses. **b**, Scatterplots showing the fraction of SDGD^dsx^ transgene inheritance (*y* axis) against sex bias (*x* axis) in the progeny of individual SDGD^dsx^ *trans*-heterozygous males (left; *n* = 63) and females (right; *n* = 39) crossed to wild-type individuals. Individual blue and pink dots represent the progeny derived from a single female, and the red dot indicates the average of the population. Error bars correspond to s.d. Both male and female SDGD^dsx^-heterozygous mosquitoes showed super-Mendelian inheritance of the transgene determined by scoring the presence of the RFP marker in the progeny. Male SDGD^dsx^-mosquitoes showed a strong bias in sex ratio toward males (0.93 ± 0.09). Dotted lines indicate the fraction of males (*x* axis) and the fraction of SDGD (*y* axis) as expected by Mendelian inheritance.

To maximize the performance of the SDGD, we developed the construct SDGD^dsx^, containing the *zpg*-Cas9 transcription unit, *beta2*^*244*^*-*I-PpoI and a guide RNA (gRNA) designed to target the intron 4–exon 5 boundary of the *dsx* gene (*AGAP004050*), because we previously reported that this site minimizes the development of resistance to a gene drive^17^. In addition, females that are homozygous for *dsxF* exhibit an ‘inter-sex’ phenotype and are viable but unable to bite^17^; therefore, this affects the vector competence of the population earlier than an SDGD targeting a standard female fertility locus, in which homozygous females are sterile but can bite and transmit. Unlike SDGD^007280^, SDGD^011377^ and SDGD^005958^, SDGD^dsx^ had no measurable impact on the fertility of heterozygotes: the larval output of SDGD^dsx^ males was comparable to that of controls (126.7 ± 50.7 (s.d.) and 140.8 ± 40.8 (s.d.), respectively; *P* = 0.39; Fig. 2a and Supplementary Table 1). The fertility of SDGD^dsx^ heterozygous females, measured as viable offspring, was reduced as compared to controls (98.8 ± 63 (s.d.) and 140.8 ± 40.8 (s.d.), respectively; *P* = 0.012), although it was still sufficient to produce a large number of fertile individuals (Fig. 2a). High levels of maternal nuclease deposition can affect the fertility of the female progeny^17,18,20^; however, we did not observe a significant difference in fertility when comparing females inheriting the transgene from a transgenic female parent to those inheriting the trangene from a male parent (Supplementary Fig. 7). As expected, we observed a marked male bias (93.1% ± 0.08% (s.d.)) in the offspring of SDGD^dsx^ heterozygous males (Fig. 2b). The sex-distortion phenotype was stably transmitted from male mosquitoes to their transgenic male offspring, and no differences were observed as compared to males that inherited the construct from a female or a male (Supplementary Table 3). Strong super-Mendelian inheritance of the construct of 96.0% ± 0.08% (s.d.) and 99.9% ± 0.01% (s.d.) was observed from both males and females, respectively, based on the frequency of red fluorescent protein (RFP)^+^ progeny from heterozygous parents (Fig. 2b), making SDGD^dsx^ suitable for population suppression experiments.

### SDGD^dsx^ invades caged mosquito populations

**Fig. 3:**
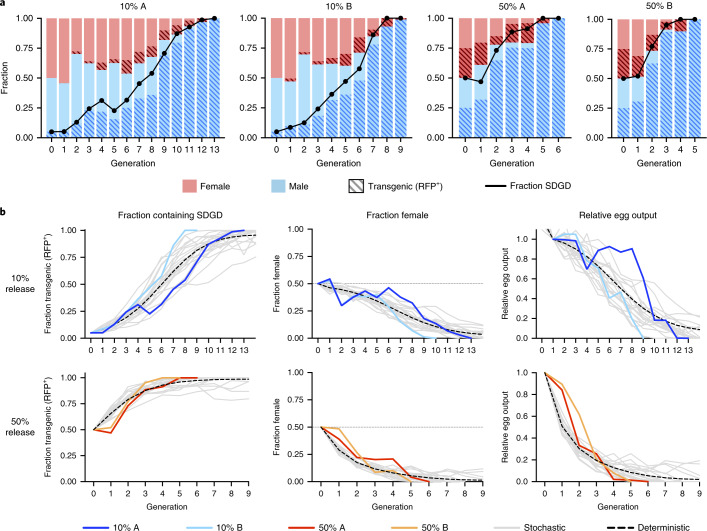
Kinetics of SDGD^dsx^ spread in target mosquito populations. The spread of SDGD^dsx^ was investigated in two different experiments starting with an allelic frequency of 2.5% (10% male release) and 25% (50% male and female release), in replica (cage A and cage B). The 10% release cages were set up with a starting population of 300 wild-type females, 270 wild-type males and 30 SDGD^dsx^-heterozygous males. The 50% release cages were started with 150 wild-type females, 150 wild-type males, 150 SDGD^dsx^-heterozygous males and 150 SDGD^dsx^-heterozygous females (allelic frequency of 25%). Each consecutive generation was established by selecting 600 larvae. The frequency of the transgene (fraction of RFP^+^ individuals), the sex ratio (female/male) and the relative egg output (fraction of eggs produced relative to the first generation) were recorded at each generation. **a**, The bar plots represent the fraction of males and females (blue and pink shading, respectively) for each population, and the striped pattern shows the fraction of transgenic individuals. Black lines indicate the total fraction of individuals containing SDGD^dsx^ (as a fraction of RFP^+^) individuals. **b**, The frequency of the transgene, the sex ratio and the relative egg output superimposed on both a deterministic model (black dashed lines) and 20 representative stochastic simulations (gray solid lines) of the dynamics of invasion of SDGD^dsx^ based on release scenarios of 25% and 2.5% SDGD^dsx^ allelic frequency. In 93% and 98% of the stochastic simulations (of 10,000 runs), the release of SDGD-heterozygous individuals at a starting frequency of 2.5% and 25%, respectively, is predicted to collapse the population within 30 generations. Dotted lines indicate the expected Mendelian distribution of sex. Fitness and life history parameter estimates are provided in Supplementary Table 2.

We used fertility, inheritance bias, sex-distortion data and mutant phenotype information to develop both deterministic and stochastic discrete-generation models (Methods and Supplementary Tables 2, 4 and 5) to predict the spread of SDGD^dsx^ into mosquito populations, simulating the release of 10% and 50% SDGD^dsx^-heterozygous mosquitoes into caged populations of 600 individuals. The stochastic model predicted that the transgene would quickly invade the population, reaching 100% allelic frequency and leading to collapse of the population in 93% and 98% of 10,000 simulations after 30 generations from a 10% and 50% SDGD release, respectively (Fig. 3). The deterministic model, however, showed differences in outcome depending on the values for the fertility of heterozygous females and males, ranging from population elimination to suppression and to the disappearance of SDGD^dsx^ if male fertility was below 0.5 compared to wild type (Supplementary Figs. 8 and 9). To test the model prediction, we released SDGD^dsx^ heterozygotes at either 2.5% or 25% allelic frequency into two populations of 600 caged mosquitoes, each in two replicates. At each generation, larvae were screened for the presence of the fluorescence marker linked to the transgene, and subsequently the fraction of males and females in the population was assessed. We observed a rapid spread of SDGD^dsx^ in all four populations, with the transgene reaching 100% allelic frequency between 4 and 12 generations. The spread of SDGD^dsx^ induced a strong bias of the population sex ratio toward males, accompanied by a progressive reduction of egg output, which led to population elimination at generations 5 and 6 for the replica cages that started with 25% SDGD^dsx^ allelic frequency and at generations 9 and 13 for the replica cages that started with 2.5% SDGD^dsx^ release (Fig. 3).

### Fitness of female progeny in SDGD^dsx^ males

SDGD-heterozygous males generated < 6% female progeny, and female offspring inherited an X chromosome from male gametes exposed to the I-PpoI nuclease during spermatogenesis. We investigated whether the inheritance of a potentially damaged X chromosome affected female fertility and the SDGD homology-directed repair rate. We crossed females that carried one ‘I-PpoI-exposed’ X chromosome from the father to wild-type males and compared their fertility parameters to those of daughters of SDGD^dsx^ females that carried two unaffected copies of the X chromosome. We observed that females inheriting one I-PpoI-exposed X chromosome did not significantly differ in fertility (measured as the number of hatched larvae) nor in drive inheritance, suggesting that, if there is a contribution to fitness of a damaged X chromosome in females, this was not detectable in our assay. To further investigate the potential impact of I-PpoI-exposed X chromosomes, we modeled additional fitness reductions in individuals with a damaged X chromosome using deterministic discrete-generation cage simulations of a theoretical scenario of SDGD^dsx^ release (10% males and 50% males/50% females) into a caged population (Supplementary Fig. 10). The model predicted little or no effect during the initial spread of the transgene, but a reduction in the suppression load that correlated with the cost of the damaged X chromosome was detected (Supplementary Fig. 10).

### Dynamics of sex-distorter drive

Driving a sex distorter into a female fertility locus could impose a sufficiently high load on the population to the point that the population is suppressed and eliminated. However, the dynamics of an SDGD are complex and depend not only on the fertility of SDGD^dsx^-heterozygous individuals (Supplementary Figs. 8 and 9) but also on the rate of male bias (Supplementary Fig. 11), and in certain scenarios these dynamics are not intuitive. For example, when female (W/D, where W is the wild-type allele and D represents the SDGD allele) fertility is reduced, such as below 0.5, the load on the population increases with increasing sex distortion, whereas for higher female fertility, such as above 0.5, the load is greater when there is no sex distortion (equivalent to a gene drive without a sex distorter, *m* = 0.5; Supplementary Fig. 11). The sex distorter allows the SDGD construct to spread at low (or even zero) female fertility, imposing a substantial load (Supplementary Fig. 12). This is because the male bias mitigates the effect of low female fitness. Overall, increasing sex distortion makes the construct less sensitive to variation in female heterozygous fertility (Supplementary Fig. 12). At the limit of complete male bias (male progeny = 100%), the load is independent of female fertility because no SDGD females are created and only SDGD-heterozygous males can pass on the construct. Based on our experimental parameter estimates for SDGD^dsx^, the SDGD allele is predicted to be present in an intermediate equilibrium with wild-type and nonfunctional resistance alleles at a sufficient frequency to induce a dramatic population reduction and possibly prevent reinvasion events (Supplementary Fig. 13).

## Discussion

Our results show that SDGD^dsx^ functions as a sex-distorter autosomal gene drive. In four cage experiments, SDGD^dsx^ progressively biased the sex ratio toward males, with eventual population collapse. Notably, we did not observe the development of functional mutations at the target *dsx* site that blocked the spread of the distorter. This observation further supports the notion that the *dsx* sequence at the intron 4–exon 5 boundary is highly functionally constrained and validates its use as a target sequence for gene-drive solutions in anopheline mosquitoes. It should also be noted that a sex distorter that simultaneously destroys the female isoform of the *dsx* gene while reducing the female population also decreases the opportunity of resistant mutations to arise (because they are not selected in males). In addition, targeting a sequence present in hundreds of copies on the X chromosome reduces the likelihood that nuclease-induced resistance will evolve to block the sex-distorter component.

Our SDGD solution also combines a number of features in terms of efficacy, robustness and predicted time to impact (on disease transmission), which differ from those for previously described gene drives or autosomal sex-distorter systems, making it particularly attractive for field implementation (Table 1). In two replicate caged experiments, SDGD^dsx^ consistently induced population collapse starting from an allelic frequency of 2.5%. For field experiments, this translates into mosquito numbers to be released that are within the range of production capability; recent studies modeling the impact of hypothetical X-shredder Y-drive mosquitoes on a national scale predict that the release of as few as ten males in 1% of human settlements will achieve over 90% population suppression after 4 years^22^.

SDGD^dsx^ is predicted to show a higher level of robustness than a gene drive alone, even if one of the critical components breaks down or mutates, due to the synergy of the components. Loss or inactivation of the I-PpoI sequence will result in the generation of functional *dsx* gene drive that will also contribute to population suppression (Supplementary Fig. 12), and loss of function of either of the two drive components (Cas9 or gRNA) will produce nonfunctional *dsx* alleles (R) that, in heterozygous individuals, will still contribute to the production of male-biased progeny owing to the presence of functional I-PpoI. Mutations and recombination events of the constructs involving both the drive and distorter will generate R nonfunctional *dsx* mutations. These R mutations are constantly generated at the target locus by the action of the nuclease^17^, but they are not selected because they do not restore function of the *dsx* gene and homozygous R females are sterile; therefore, they are continuously lost as they arise.

Modeling based on our experimental data shows that SDGD^dsx^ offers some important advantages in short-term drive dynamics and long-term outcomes. Importantly, the number of transmission-competent (that is, biting) females is reduced more quickly by SDGD^dsx^ than by a standard gene drive targeting the same locus (Supplementary Fig. 1), which could lead to a strong effect on disease transmission (time to impact after release). In comparing a distorting and a non-distorting gene drive, the equilibrium load imposed by SDGD^dsx^ is less sensitive to female fitness costs, which is particularly relevant given the uncertain extrapolation of fitness effect measurements from the lab to the field.

Previous modeling of gene drive without the sex distorter showed that under certain conditions (for example, leaky expression of the drive construct) there can be an accumulation of nonfunctional cleavage-resistant sequences, which prevents the transgene from going to fixation^17,23^. Deterministic modeling of SDGD^dsx^ indicates that there is also the potential for the transgene to go to an intermediate equilibrium frequency and population suppression, rather than complete fixation and elimination (Supplementary Figs. 8, 9 and 12). The lower the SDGD fertility in heterozygous individuals, the more likely an intermediate equilibrium is reached. For the observed fertility values of SDGD^dsx^-heterozygous females, stochastic models predicted population elimination for finite cage populations in 93–98% of the simulations, with kinetics of spread in line with observed data. Under field or semi-field conditions, the fertility estimates of heterozygous individuals could differ and tilt the balance one way or the other toward population reduction rather than population elimination. Achieving a strong population reduction may be regarded as less effective than elimination in a field scenario; however, it could help achieve long-term stable vector control via a higher tolerance to repopulation through migration as compared to a system that quickly eliminates an entire target population.

Males carrying a non-driving I-PpoI construct designed to cause dominant male sterility^21^ were recently released in a field location of Burkina Faso^24^ as part of a phased, step-by-step assessment of novel genetic approaches to malaria control, following independent guidance and recommendations^25,26^. This opened the way to the use of an I-PpoI-based distorter for the implementation of genetic vector control measures.

We believe that SDGD^dsx^ outperforms other anopheline gene drives, combining efficacy, resistance management and robustness, and is well suited as an anti-malaria intervention.

## Methods

### Ethics statement

#### Generation of SDGD constructs

To create SDGD vectors p172 (*vas2*; GenBank accession MT270142) and p182 (*zpg*), the β2-eGFP(F2A)I-PpoI transcription unit from pBac[3xP3-DsRed]β2-eGFP::I-PpoI-124L^13^ was excised by AscI digestion and cloned into AscI-digested p165 (*vas2*-CRISPR^h^ (ref. ^18^): GenBank accession KU189142) and p174 (*zpg*-CRISPR^h^ (ref. ^19^); GenBank accession MH541847), respectively. SDGD vectors were further modified by BsaI-mediated Golden Gate assembly to contain gRNA spacers targeting *AGAP011377* (GCAGACGTAGAAATTTTC), *AGAP007280* (GGAAGAAAGTGAGGAGGA), *AGAP005958* (GAGATACTGGAGCCGCGAGC)^18^ and *AGAP004050* (GTTTAACACAGGTCAAGCGG)^17^. To include the *beta2*^*244*^ promoter modification, plasmid p182 was further modified to generate p182–244 (GenBank accession MT270141) according to the *beta2*^*244*^ variant described below. Additional sequences of all vectors are available as Supplementary Information.

### Microinjection of embryos and selection of transformed mosquitoes

All mosquitoes were reared under standard conditions of 80% relative humidity and 28 °C. The mosquitoes were blood fed on anesthetized mice or by Hemotek, and freshly laid embryos were aligned and used for microinjections as described previously^27^. To generate SDGD mosquitoes, we injected respective docking line^17,18^ embryos with solution containing p174 or p182–244 and a plasmid-based source of PhiC31 integrase^28^ (at 200 ng μl^–1^ and 400 ng μl^–1^, respectively). All surviving G_0_ larvae were crossed to wild-type mosquitoes, and G_1_ positive transformants were identified using a fluorescence microscope (Nikon, Eclipse TE200) as RFP^+^ larvae for the recombination-mediated cassette exchange (RMCE) events.

### Containment of gene-drive mosquitoes

All mosquitoes were housed at Imperial College London in an insectary that is compliant with Arthropod Containment Guidelines Level 2 (ref. ^29^). All genetically modified (GM) work was performed under institutionally approved biosafety and GM protocols. In particular, GM mosquitoes containing constructs with the potential to show gene drive were housed in dedicated cubicles, separated from the external environment by at least six doors requiring two levels of security card access. Moreover, because the insectary is located in a city with a northern temperate climate, *A. gambiae* mosquitoes are also ecologically contained. The physical and ecological containment of the insectary are compliant with guidelines set out in a recent commentary calling for safeguards in the study of synthetic gene-drive technologies^30^.

### Mutagenesis of the *beta2-tubulin* promoter

Bioinformatic analysis of the regulatory region of the *beta2*-*tubulin* gene (*AGAP008622*) was performed using the Promoter2.0 Prediction Server^31^ and the Neural Network Promoter Prediction tool^32^ to identify the conserved region. A synthetic 100-bp DNA sequence with a G+C content of 65% (sequence reported in Supplementary Fig. 3) was designed using Geneious R11 (https://www.geneious.com/) and cloned into the *beta2-tubulin* promoter at position −244, −271 or −355 with respect to the ATG start codon using site-specific mutagenesis of plasmid pBac[3xP3-DsRed]β2-eGFP::I-PpoI-124L^13^ by nested PCR with primer pairs B2–355_r and B2–355_f, B2–271_r and B2–271_f, and B2–244_r and B2–244_f followed by Spac-fwd and Spac-rev, for the *beta2*^*355*^, *beta2*^*271*^ and *beta2*^*244*^ variants, respectively. A second unmodified copy of the *beta2-tubulin* promoter was cloned to express the mCherry gene.

**Table:** 

Name	Sequence
B2–355_r	GGCCAACTCGGGTCCGAGTCGTCTTCTTGGATGGGATGATG
B2–355_f	CGCCAGCACTCTCAGACTCAATACGAATTTATTTGTGGCATCG
B2–271_r	GGCCAACTCGGGTCCGAGTCATATGACTACTATGATCATCTTTTGC
B2–271_f	CGCCAGCACTCTCAGACTCAGAG CCG TAC GTG CCG G
B2–244_r	GGCCAACTCGGGTCCGAGTCCACGAAATGATCCGGCAC
B2–244_f	CGCCAGCACTCTCAGACTCACAGAACCTTCAGAGACGTTG
Spac-fwd	GTGAGAAGTGCGCGTCTCGTTCCCGCAGCTCGCCAGCACTCTCAGACTCA
Spac-rev	CATCCGCCCTAACTCCGCCCGTGGGTCGTTGGCCAACTCGGGTCCGAGTC

### Dual-fluorescence assay experiment

Three- to five-day-old adult male heterozygous mosquitoes were collected in Falcon tubes and anesthetized on ice 5 min before dissection. Testes were micro-dissected using an Olympus SZX7 optical microscope, and pictures of gonads were taken using the EVOS imaging system (Thermo-Fisher) with magnification of ×20 and the following exposure settings: bright field: gain 50%; GFP channel: gain 30%, 120 ms; RFP channel: gain 80%, 120 ms. Unmodified pictures were then analyzed using ImageJ software^33^. Testis areas were selected using the freeform selection tool, and integrated density and mean gray values were measured for the GFP and RFP channels independently using the same selection area. A reading for the background (same selection area) was then subtracted from the integrated density value for each testis to remove background noise. The value for fluorescence intensity was measured as the ratio between the GFP reading and the mCherry reading and normalized to the value of the unmodified *beta2-tubulin* control.

### Phenotypic assays

Phenotypic assays designed to examine SDGD inheritance and relative fecundity in mosquitoes were carried out essentially as described before^17,18^. Briefly, the offspring of heterozygous individuals crossed to wild-type counterparts were screened by RFP expression. Non-fluorescent progeny were kept as controls. Groups of 50 male and 50 female blood-fed mosquitoes were mated to an equal number of wild-type mosquitoes for 5 d, and a minimum of 40 females were allowed to lay individually. The entire egg and larval progeny was counted for each lay. Females that failed to give progeny and had no evidence of sperm in their spermathecae were excluded from the analysis. To determine the inheritance and sex-ratio bias of SDGD, the entire larval progeny was screened for the presence of DsRed, which is linked to the SDGD allele, and all the progeny was sexed at the pupal stage to determine the sex ratio. Statistical differences between genotypes were assessed using the Kruskal–Wallis test.

### Cage trial assays

To perform cage trials of SDGD^011377^ and SDGD^005958^, we introduced 100 heterozygous transgenic males into a population of 100 wild-type males and 200 wild-type females (transgenic allelic frequency of 12.5%) in triplicate. As a control, 100 heterozygous transgenic males from the autosomal self-limiting sex-distorter ^gfp^124L-2 line^13^ were released at the same frequency in a separate population, in triplicate. In addition, a population of 200 wild-type males and 200 wild-type females served as a negative control.

For the starting generation only, age-matched male and female pupae were allowed to emerge in separate cages and were mixed only when all the pupae had emerged. Mosquitoes were left to mate for 5 d before they were blood fed on anesthetized mice. After 2 d, the mosquitoes were set to lay in a 300-ml egg bowl filled with water and lined with filter paper. The eggs produced from the cage were photographed and counted using JMicroVision v1.27. Before counting, the eggs were dispersed using gentle water spraying in the egg bowl to homogenize the population, and 450 eggs were randomly selected to seed the next generation. Larvae emerging from the 450 eggs were counted and screened for the presence of the RFP marker to score the transgenic rate of the progeny. All the pupae were sexed to determine the sex ratio of the population.

To perform cage trials of SDGD^dsx^, we set up two different experiments, in replicate. The 10% release cages were set up with a starting population of 300 wild-type females, 270 wild-type males and 30 SDGD^dsx^-heterozygous males (starting allelic frequency of 2.5%). The 50% release cages were started with 150 wild-type females, 150 wild-type males, 150 SDGD^dsx^-heterozygous males and 150 SDGD^dsx^-heterozygous females (allelic frequency of 25%). For the starting generation only, age-matched male and female pupae were allowed to emerge in separate cages and were mixed only when all the pupae had emerged. Mosquitoes were left to mate for 5 d before blood feeding. After 2 d, the mosquitoes were set to lay in a 300-ml egg bowl filled with water and lined with filter paper. All larvae were allowed to hatch, and each consecutive generation was established by randomly selecting 600 larvae, split into 3 trays of 200 larvae each. All 600 larvae were screened for the presence of the RFP marker, and the pupae from one tray were sexed to determine the sex ratio. On day 8, mosquitoes were offered a second blood meal, and all the eggs produced were photographed and counted using Egg counter software^34^.

### Statistical analysis

Statistical analysis was performed as indicated using GraphPad Prism version 7.0.

### Population genetics model

#### Discrete time

To model the results of the cage experiments, we use discrete-generation recursion equations for the genotype frequencies, with males and females treated separately, similarly to Kyrou et al.^17^. We extend the previous study^17^ to model the SDGD by including a sex bias and possible X-chromosome damage in the progeny of SDGD males, although here we do not include parental effects on fitness (as these effects were not strongly observed). We consider three alleles at the female fertility target site, W (wild type), D (driving sex distorter) and R (nonfunctional nuclease resistant). We also differentiate between the two possible types of X chromosome: x (wild type) and X, which denotes an X chromosome that has passed through an SDGD male and survived X-shredding but may be damaged, resulting in an additional fitness cost to the individual carrying it. $F_{ij,pq}(t)$ and $M_{ij,qY}\left( t \right)$ denote the genotype frequency of females and males, respecitvely, in the total population, where the first set of indices denotes alleles at the target site {WW, WD, WR, DD, DR, RR} and the second set denotes the sex chromosomes, *pq* = {xx, xX, XX} for females and *q* = {x, X} for males. There were 18 female genotypes and 12 male genotypes; six types of eggs: *E*_W,x_, *E*_D,x_, *E*_R,x_, *E*_W,X_, *E*_D,X_ and *E*_R,X_, where the first index refers to the target site allele and the second index to the sex chromosome; and eight types of sperm: *S*_W,X_, *S*_R,X_ (no *S*_D,x_, because we assume that SDGD males only contribute X chromosomes), *S*_W,X_, *S*_D,X_, *S*_R,X_, *S*_W,Y_, *S*_D,Y_ and *S*_R,Y_.

#### Homing

Adults of genotype W/D at the target site produced gametes at meiosis in the ratio W:D:R as follows:

$$\left( {1 - d_{\rm{f}}} \right)\left( {1 - u_{\rm{f}}} \right):d_{\rm{f}}:\left( {1 - d_{\rm{f}}} \right)u_{\rm{f}}\;{\textrm{in}}\;{\textrm{females}}$$

$$\left( {1 - d_{\rm{m}}} \right)\left( {1 - u_{\rm{m}}} \right):d_{\rm{m}}:\left( {1 - d_{\rm{m}}} \right)u_{\rm{m}}\;{\textrm{in}} \;{\textrm{males}}$$

Here *d*_f_ and *d*_m_ are the rates of transmission of the driver allele in the two sexes and *u*_f_ and *u*_m_ are the fractions of non-drive gametes at the target site that are repaired by meiotic end-joining and are nonfunctional and resistant to the drive (R). In all other genotypes, inheritance at the target site is Mendelian.

#### Sex distortion

The SDGD X-shredder only affects the sex ratio of the progeny if it is in males. It destroys the X chromosome while males are making their sperm, resulting in mostly Y-bearing sperm. From male SDGD heterozygotes, progeny will therefore consist of *m*_1_ (1/2 < *m*_1_ ≤ 1) males and (1 − *m*_1_) females; from male SDGD homozygotes (D/D), the progeny will be *m*_2_ (1/2 < *m*_2_ ≤ 1) males and (1 − *m*_2_) females. For simplicity, when comparing to the experiment, we assume *m*_1_ = *m*_2_ = *m*. We assume no mutations cause loss of function of the sex distorter from the construct or resistance to X-shredding.

All X chromosomes contributed by SDGD males that survived X-shredding are assumed to be ‘damaged’ X chromosomes (versus wild-type X chromosomes), reflected in the reduced reproductive fitness of the individual carrying it (see ‘Fitness’). We assume a damaged X chromosome was susceptible to further shredding if it was inherited by an SDGD male and, for simplicity, that the fitness cost of carrying a damaged X chromosome is the same no matter how many times the chromosome passes through an SDGD male and survives X-shredding.

#### Fitness

We let $w_{ij,pq},w_{ij,qY} \le 1$ represent the reproductive fitnesses of female and male genotypes relative to a fitness of 1 for wild-type homozygotes, where {*ij*} denotes alleles at the target site of the construct {WW, WD, WR, DD, DR, RR} and the second set of indices denotes *pq* = {xx, xX, XX} for females and *q* = {x, X} for males. While all fitness parameters are retained in the recursion equations for generality, for comparison with the experiment, we assume that the target gene is needed for female fertility; thus, females with D/D, D/R and R/R at the target site are sterile. There is no reduction in fitness in W/R females from carrying only one copy of the target gene (W/R), but W/D females have reduced fitness due to the presence of the SDGD construct, as observed experimentally (Supplementary Table 2). We assume no costs to males with no copies of the driving sex distorter (W/R, R/R), but that males with one or two copies of the SDGD (W/D, D/D, D/R) have a fitness reduction consistent with experimental observation (Supplementary Table 2).

If the individual also carries a damaged X chromosome, we assume that this imposes an additional cost that affects reproductive success. To calculate the overall fitness of the genotype, the fitness value associated with carrying the damaged X chromosome is multiplied by the fitness value associated with D (or R) alleles at the target site (Supplementary Tables 4 and 5). Reduced fitness in males with a copy of the damaged X chromosome is (1 − *s*_X,m_) and in females with two copies of the damaged X chromosome is (1 − *s*_X,f_), with *s*_X,f_, *s*_X,m_ = 0 if there is no cost and 1 if the damaged X chromosome causes sterility. For females with one damaged X chromosome and one wild-type x chromosome, the reduction is (1 − *h*_X,f_*s*_X,f_), where *h*_X,f_ is the dominance coefficient (0 for fully recessive; 1 for fully dominant). For baseline parameters, we assume these costs are equal to zero.

#### Recursion equations

We first consider the gamete contributions from each genotype. The proportions *E*_*k*,*l*_(*t*) with allele *k* = {W, D, R} at the target site and sex chromosome *l* = {x, X} in eggs produced by females participating in reproduction are given in terms of the female genotype frequencies $F_{ij,pq}\left( t \right)$:

1 $$E_{k,l}\left( t \right) = \frac{{\mathop {\sum }\nolimits_{i = 1}^3 \mathop {\sum }\nolimits_{j = i}^3 \mathop {\sum }\nolimits_{pq = {\textrm{xx; xX; XX}}} \left( {c_{ij,pq}^{k,l}w_{ij,pq}F_{ij,pq}\left( t \right)} \right)}}{{\mathop {\sum }\nolimits_{i = 1}^3 \mathop {\sum }\nolimits_{j = i}^3 \mathop {\sum }\nolimits_{pq = {\textrm{xx; xX; XX}}} \left( {w_{ij,pq}F_{ij,pq}\left( t \right)} \right)}}$$

where *i* and *j* are each summed such that {1, 2, 3} corresponds to {W, D, R}. The coefficients $c_{ij,pq}^{k,l}$ in equation (1) correspond to the proportion of the gametes from female individuals of genotypes (*ij*, *pq*) that carry alleles (*k*, *l*), as shown in Supplementary Table 4 (rows correspond to genotypes; columns correspond to alleles).

The proportions $S_{k,l}(t)$ with allele *k* = {W, D, R} at the target site and sex chromosome *l* = {x, X, Y} in sperm are given in terms of the male genotype frequencies $M_{ij,qY}\left( t \right)$:

2 $$S_{k,l}\left( t \right) = \frac{{\mathop {\sum }\nolimits_{i = 1}^3 \mathop {\sum }\nolimits_{j = i}^3 \mathop {\sum }\nolimits_{q = {\textrm{x, X}}} \left( {c_{ij,qY}^{k,l}w_{ij,qY}M_{ij,qY}(t)} \right)}}{{\mathop {\sum }\nolimits_{i = 1}^3 \mathop {\sum }\nolimits_{j = i}^3 \mathop {\sum }\nolimits_{q = {\textrm{x, X}}} \left( {w_{ij,qY}M_{ij,qY}(t)} \right)}}$$

where, again, *i* and *j* are each summed such that {1, 2, 3} corresponds to {W, D, R}. The coefficients $c_{ij,qY}^{k,l}$ in equation (2) correspond to the proportion of the gametes from male individuals of type (*ij*, *qY*) that carry alleles (*k*, *l*), as shown by the rows and columns, respectively, in Supplementary Table 5. Note that *S*_*D*,*x*_(*t*) = 0 because SDGD males only contribute damaged X chromosomes, so no entry for this is included in Supplementary Table 5.

We define the proportion of females in the population as

$$F(t) = \mathop {\sum}\limits_{i = 1}^3 {\mathop {\sum}\limits_{j = i}^3 {\mathop {\sum}\limits_{pq = {\textrm{xx; xX; XX}}} {F_{ij,pq}} } } \left( t \right)$$

and the average female reproductive fitness as

$$\bar w_f(t) = \mathop {\sum }\limits_{i = 1}^3 \mathop {\sum }\limits_{j = i}^3 \mathop {\sum }\limits_{pq = {\textrm{xx; xX; XX}}} \left( {\frac{{w_{ij,pq}F_{ij,pq}\left( t \right)}}{{F(t)}}} \right)$$

Analogously, for the male proportion, we define the proportion of males in the population as

$$M(t) = \mathop {\sum }\limits_{i = 1}^3 \mathop {\sum }\limits_{j = i}^3 \mathop {\sum }\limits_{q = {\textrm{x,X}}} M_{ij,qY}(t)$$

and the average male fitness as

$$\bar w_m(t) = \mathop {\sum }\limits_{i = 1}^3 \mathop {\sum }\limits_{j = i}^3 \mathop {\sum }\limits_{q = {\textrm{x,X}}} \left( {\frac{{w_{ij,qY}M_{ij,qY}\left( t \right)}}{{M(t)}}} \right)$$

Note that in equations (1) and (2), the normalization factor in the denominator is therefore $\bar w_{\rm{f}}(t)F(t)$ and $\bar w_{\rm{m}}(t)M(t)$, respectively.

The load on the population incorporates reductions in female and male fertility and decreased frequency of females due to the SDGD spreading in the population, and at time (*t*) is defined as

$$L(t) = 1 - 2F(t)\bar w_{\rm{f}}(t)\bar w_{\rm{m}}(t)$$

This equals zero when only wild-type individuals are present and one if the SDGD has been established and the average female fitness, or fraction of females present, is equal to zero. We note that increases in load predicted by the cage model do not predict absolute changes in population density in the field but can be an indication of comparative potential reductions^35^.

To model cage experiments, we start with an equal number of males and females. For 50% release, the initial frequency for wild-type females and males is $F_{{\mathrm{WW}},{\mathrm{xx}}} = M_{{\mathrm{WW}},{\mathrm{xY}}} = 1/4$ and for heterozygote drive females and males is $F_{{\mathrm{WD}},{\mathrm{xx}}} = M_{{\mathrm{WD}},{\mathrm{xY}}} = 1/4$. For 10% release of males only, $M_{{\mathrm{WW}},{\mathrm{xY}}} = 9/20$ and $M_{{\mathrm{WD}},{\mathrm{xY}}} = 1/20$ and all females are wild type, $F_{{\mathrm{WW}},{\mathrm{xx}}} = 1/2$. Assuming random mating, we obtain the following recursion equations for the female genotype frequencies in generation (*t* + 1):

$$\begin{array}{*{20}{l}} {F_{ij,pq}\left( {t + 1} \right)} \hfill & = \hfill & {\left( {1 - \frac{{\delta _{ij}}}{2}} \right)\left( {1 - \frac{{\delta _{pq}}}{2}} \right)} \hfill \\ {} \hfill & {} \hfill & {\left( {E_{i,p}\left( t \right)S_{j,q}\left( t \right) + E_{j,p}\left( t \right)S_{i,q}\left( t \right) + E_{i,q}\left( t \right)S_{j,p}\left( t \right) + E_{j,q}\left( t \right)S_{i,p}\left( t \right)} \right)} \hfill \end{array}$$

where *pq* = {xx, xX, XX}, and *δij* is the Kronecker delta. The factors $\left( {1 - \frac{{\delta _{ij}}}{2}} \right),\left( {1 - \frac{{\delta _{pq}}}{2}} \right)$ account for the factor of 1/2 for homozygosity at the target site (for *ij* = {W/W, D/D, R/R}) and at the sex chromosomes (for *pq* = {xx, XX}). We obtain the following recursion equations for the male genotype frequencies:

$$M_{ij,qY}\left( {t + 1} \right) = \left( {1 - \frac{{\delta _{ij}}}{2}} \right)\left( {E_{i,q}\left( t \right)S_{j,Y}\left( t \right) + E_{j,q}\left( t \right)S_{i,Y}\left( t \right)} \right)$$

where *q* = {x, X} and $\left( {1 - \frac{{\delta _{ij}}}{2}} \right)$ accounts for the factor of 1/2 for homozygosity at the target site (for *ij* = {W/W, D/D, R/R}).

#### Stochastic version

In the stochastic version of the model described above, random values for probabilistic events are taken from the appropriate multinomial distributions, with probabilities estimated from the experiment where applicable (Supplementary Table 2). To model the cage experiments, 150 female and 150 male wild-type adults (or 300 females and 270 males for 10% release of males only) along with 150 female and 150 male heterozygotes (or no females and 30 males for 10% release) are initially present. Females may fail to mate or mate once in their life with a male of a given genotype, according to its frequency in the male population, chosen randomly and with replacement such that males may mate multiple times. The number of eggs from each mated female is multiplied by the egg production of the male relative to the wild-type male, to account for experimental observations of reduced egg production from SDGD fathers. The eggs may hatch or not, with a probability that depends on the product of larval hatching values from the mother and father, relative to wild type. To start the next generation, 600 larvae are randomly selected, unless fewer than 600 larvae have hatched, in which case the smaller amount initiates the next generation, following the experiment. The probability of subsequent survival to adulthood is assumed to be equal across genotypes. Assuming very large population sizes allows results for the genotype frequencies that are indistinguishable from the deterministic model. For the deterministic egg count, we use the large population limit of the stochastic model.

#### Population dynamics model (continuous time)

To model changing population sizes in the field (for Supplementary Fig. 1), we use a continuous-time population dynamics model with one life stage and logistic density dependence in the recruitment rate based on models developed previously^36,37^. Here $n\left( t \right)$ represents the abundance of adult individuals, *f*(*t*) and *m*(*t*) represent the total abundances of adult females and males, and $f_{ij,pq}\left( t \right)$ and $m_{ij,qY}\left( t \right)$ are the genotype abundances where, as above, the first set of indices denotes alleles at the target site and the second set denotes the sex chromosomes. Populations are normalized to the prerelease wild-type population size such that *n*(*t* = 0) = 1, and time is continuous and measured in generations. The dynamics of the total population size are given by the following differential equation:

$$\frac{{dn\left( t \right)}}{{dt}} = 2\left( {\frac{{R_{\rm{m}}}}{{1 + 2(R_{\rm{m}} - 1)\bar w_{\rm{m}}(t)\bar w_{\rm{f}}(t)f(t)}}} \right)\bar w_{\rm{m}}(t)\bar w_{\rm{f}}(t)f\left( t \right) - n\left( t \right)$$

The total recruitment rate of adults incorporates a density-dependent factor (the term in parentheses) based on Deredec et al.^5^ and depends on the total number of females, *f*(*t*), multiplied by the average female fitness, $\bar w_{\rm{f}}(t) = \mathop {\sum}\nolimits_{i = 1}^3 {\mathop {\sum}\nolimits_{j = i}^3 {\mathop {\sum}\nolimits_{pq = {\textrm{xx;xX;XX}}} {\left( {w_{ij,pq}f_{ij,pq}\left( t \right)/f(t)} \right)} } }$. Because SDGD males may have reduced fertility, the recruitment rate is also dependent on the average male fitness, $\bar w_{\rm{m}}(t) = \mathop {\sum}\nolimits_{i = 1}^3 {\mathop {\sum}\nolimits_{j = i}^3 {\mathop {\sum}\nolimits_{q = {\textrm{x,X}}} {\left( {w_{ij,qY}m_{ij,qY}\left( t \right)/m(t)} \right)} } }$ (we assume that the number of males is not limiting and that all males participate in mating). *R*_m_ is the intrinsic growth rate of the population per generation at low density.

The equations for the individual genotype populations for females and males are

$$\begin{array}{*{20}{l}} {\frac{{df_{ij,pq}\left( t \right)}}{{dt}}} \hfill & = \hfill & {2\left( {\frac{{R_{\rm{m}}\bar w_{\rm{m}}(t)\bar w_{\rm{f}}(t)f\left( t \right)}}{{1 + 2\left( {R_{\rm{m}} - 1} \right)\bar w_{\rm{m}}\left( t \right)\bar w_{\rm{f}}\left( t \right)f\left( t \right)}}} \right)\left( {1 - \frac{{\delta _{ij}}}{2}} \right)\left( {1 - \frac{{\delta _{pq}}}{2}} \right)} \hfill \\ {} \hfill & {} \hfill & {\left( {e_{i,p}\left( t \right)s_{j,q}\left( t \right) + e_{j,p}\left( t \right)s_{i,q}\left( t \right) + e_{i,q}\left( t \right)s_{j,p}\left( t \right) + e_{j,q}\left( t \right)s_{i,p}\left( t \right)} \right) - f_{ij,pq}(t)} \hfill \end{array}$$

$$\begin{array}{*{20}{l}} {\frac{{dm_{ij,qY}\left( t \right)}}{{dt}}} \hfill & = \hfill & {2\left( {\frac{{R_{\rm{m}}\bar w_{\rm{m}}(t)\bar w_{\rm{f}}(t)f\left( t \right)}}{{1 + 2(R_{\rm{m}} - 1)\bar w_{\rm{m}}(t)\bar w_{\rm{f}}(t)f(t)}}} \right)\left( {1 - \frac{{\delta _{ij}}}{2}} \right)} \hfill \\ {} \hfill & {} \hfill & {\left( {e_{i,q}\left( t \right)s_{j,Y}\left( t \right) + e_{j,q}\left( t \right)s_{i,Y}\left( t \right)} \right) - m_{ij,qY}\left( t \right)} \hfill \end{array}$$

Egg and sperm proportions $e_{k,l}\left( t \right)$ and $s_{k,l}\left( t \right)$ are as defined in equations (1) and (2) in the discrete-generation model above, with $f_{ij,pq}\left( t \right)$ and $m_{ij,pq}\left( t \right)$ instead of frequencies $F_{ij,pq}\left( t \right)$ and $M_{ij,pq}\left( t \right)$.

All calculations were carried out using Wolfram Mathematica^38^.

### Reporting Summary

Further information on research design is available in the Nature Research Reporting Summary linked to this article.

## Online content

Any methods, additional references, Nature Research reporting summaries, source data, extended data, supplementary information, acknowledgements, peer review information; details of author contributions and competing interests; and statements of data and code availability are available at 10.1038/s41587-020-0508-1.

## Supplementary Information

### Integrated supplementary information

Supplementary Fig. 1 Model prediction of the reduction in abundance of transmission competent females. Model prediction of the reduction over time in abundance of transmission competent (that is biting) females, normalised by the initial total population size, for the SDGD^dsx^ construct compared to dsxF^CRISPRh^ (Kyrou et al., 2018), using a continuous-time population dynamics model (see Supp. modeling Methods) for ‘field' release of 1% heterozygous transgenic males in the male population. The SDGD^dsx^ construct is predicted to suppress the population of transmission competent females faster than the dsxF^CRISPRh^, mainly due to the creation of a male bias in the population by the sex distorter. Parameters used for SDGD^dsx^ are in Table S2; dsxF^CRISPRh^ parameters were estimated from Kyrou et al. (2018) using an average W/D female fitness of 0.4335; for both, R_m_ (intrinsic growth rate per generation) = 6. At long times (not shown), the SDGD^dsx^ population rebounds to an intermediate equilibrium (suppressed) population.

Supplementary Fig. 2 Sex and inheritance bias caused by SDGD^011377^ and SDGD^005958^ males. Scattered plots showing the fraction of transgene inheritance (y-axis) against male bias (x-axis) in the progeny of heterozygous male SDGD^011377^ (left-hand panel) SDGD^005958^ and (right-hand panel) crossed to wild type females. Individual coloured dots represent the progeny derived from a single female and the red dots indicate the average of the population (with respective values indicated next to the plot ± s.e.m.). Error bars indicate standard deviation. SDGD at both loci showed a high transmission rate of the transgene determined by scoring in the progeny the presence of RFP marker that is linked to the SDGD allele. The progeny of SDGD/+ at both loci showed a strong sex ratio distortion towards males. Dotted lines indicate expected Mendelian inheritance.

Supplementary Fig. 3 Fecundity phenotype of SDGD targeting 3 different fertility loci in An. gambiae. SDGD constructs expressing the Cas9 nuclease under the control of the *Vas2* promoter were generated targeting the fertility loci *AGAP011377*, *AGAP007280* and *AGAP005859* (as indicated). SDGD heterozygous male and female were crossed to wild-type counterparts. Each dot represents progeny of individual females. Fecundity was measured by counting the number of eggs per female and the hatched larvae. Values on the right represent average ± s.e.m. A strong fertility effect was observed in heterozygous SDGD females at 3 loci, while male fecundity was strongly impaired by targeting 7280 and 5859 loci. Vertical red bars indicate average count, and error bars indicate standard deviation. A minimum of 20 females were analysed for each cross.

Supplementary Fig. 4 Kinetics of SDGD^011377^ and SDGD^005958^ spread in target mosquito populations. In these experiments 100 heterozygous transgenic males were introduced into a population of 100 wild-type males and 200 wild-type females (transgenic allele frequency of 12.5%). The frequency of the transgene was monitored every generation together with the fraction of males in the population and the total number of eggs laid. Each consecutive generation was established by collecting 450 eggs. The frequency of the SDGD^011377^ (red lines) and SDGD^005958^ (blue lines) was compared to that of the autosomal self-limiting sex-distorter ^gfp^124L-2 (grey lines) (Galizi et al., 2014) as well as to that of wild-type populations (black lines) as control. Each genotype was tested in triplicate cages. The SDGD^005958^ allele disappeared from the populations at generation 2 due to the strong fertility effects. The SDGD^011377^ alleles persisted in the populations despite the fertility effects but failed to increase over the frequency of release on subsequent generations. The fraction of males in the population was stably biased to about 65%.

Supplementary Fig. 5 Mutagenesis of the beta2 tubulin promoter. (**a**) Schematic overview of the *AGAP008622* (*beta2-tubulin*) locus and the three *beta2* promoter variants generated by placing a 100bp GC-rich DNA sequence (blue rectangle) upstream of the start codon at position 244, 271 and 355. (**b**) The double-fluorescence reporter assay developed to detect the effects of the *beta2* promoter modifications on its transcription level. A construct for each modification was generated harbouring the modified *beta2* promoter (stars key) driving an eGFP marker, while a second, unmodified, *beta2* promoter was driving a mCherry protein. The constructs were integrated within the same autosomal docking line by PhiC31-mediated integration by replacing a 3xP3::CFP cassette with a 3xP3::DsRed as integration marker. C) The sequence of 100bp GC-rich DNA region inserted at the 5’ of the *beta2* promoter.

Supplementary Fig. 6 GFP and mCherry signal quantification from mosquito testes transformed with modified beta2 promoters. (**a**) Dissected testis from beta2^wt^, beta2^271^, beta2^244^ and beta2^355^ transgenic lines containing a single heterozygous insertion were microphotographed under the same exposure settings (as indicated), using mCherry as internal control. Line beta2^271^ showed GFP fluorescence intensity comparable to background; GFP expression was detectable at increased exposure (100% gain, 120ms, inset). Scale bar, 200 µm. (**b**) Quantification of fluorescence intensity as GFP/mCherry ratio, normalized to the beta2^wt^ control (100%). Average relative intensity is indicated above the bars. *** indicates P value < 0.001 (ordinary One-way ANOVA). A minimum of 31 testes were analysed from individual expressing each promoter variant.

Supplementary Fig. 7 Maternal or paternal contribution to the fecundity of the SDGD^dsx^ allele. Male and female SDGD^dsx^ heterozygotes that had inherited a maternal or paternal copy of the SDGD^dsx^ allele were crossed to wild type counterparts and assessed for fecundity. The total larval output is plotted for individual females (dots). Red bars indicate the average and the mean count (± s.e.m.) is shown. Females inheriting the transgene from the mother (G1♀ → G2♀) have significantly lower larval progeny (*P = 0.0256, Kruskal-Wallis test) compared to wild-type control.

Supplementary Fig. 8 Time dynamics of the frequency of SDGD^dsx^ as function of female fitness. Time dynamics of the frequency of SDGD^dsx^ transgenic individuals in the population as a function of W/D (where D represent the SDGD allele and W the wild-type allele) female fitness (*w*_WD,xx_), as predicted by the deterministic discrete-generation model at 25% initial allelic frequency. The graph shows the frequency of SDGD heterozygote males and females as a proportion of the male (or female) population, with other parameter estimates and baseline values given in Supp Table 2 (SDGD male fitness = 0.854; *m* (sex distortion) = 0.93). The predicted outcome at high W/D female fitness is elimination of the population, and at lower fitness, an intermediate equilibrium with W, R and D alleles. The result (black line) for the experimental estimate for female SDGD heterozygote fitness, *w*_WD,xx_ = 0.627, is in a parameter region where even a small (positive) change leads to a prediction of population elimination instead of suppression.

Supplementary Fig. 9 Time dynamics of the frequency of SDGD^dsx^ as function of male fitness. Time dynamics of the frequency of SDGD^dsx^ transgenic individuals in the population as a function of the fitness of SDGD males (assume *w*_WD,xY_ = *w*_DR,xY_ = *w*_DD,xY_) as predicted by the deterministic discrete-generation model. Initial release is 50% of SDGD heterozygote males and females as a proportion of the male (or female) population, with other parameter estimates and baseline values given in Supp Table 2 (W/D female fitness *w*_WD,xx_ = 0.627; *m* = 0.93). For low SDGD male fitness (<≈0.5), the construct is eventually lost.

Supplementary Fig. 10 Impact of fitness due to damaged X chromosome. modeling the impact of fitness reduction, due to the inheritance of damaged X chromosomes passed through X-shredding in a SDGD male, on the spread of the SDGD transgenics as predicted by the deterministic discrete-generation cage model. For simplicity, we assume that the additional cost to carrying one damaged X chromosome in males is the same as the cost in females that carry two copies of the damaged X (*s*_X,f_ = *s*_X,m_), and females with one damaged X and one wildtype X chromosome have only half the fitness cost of females with two copies (dominance coefficient *h*_X,f_ = 1/2). Estimates used for other parameters given in Supplementary Table 2.

Supplementary Fig. 11Effect of female fitness on SDGD frequency and population load.Effect of female W/D heterozygote fitness (D refers to the SDGD^dsx^ allele) on the SDGD genotype frequency (that is, individuals with at least one copy of the SDGD) and load after 200 generations, as predicted by the deterministic discrete-generation model. Parameter estimates and baseline values given in Supp Table 2 (SDGD male fitness = 0.854). The possible outcomes (load = 1 and population elimination; intermediate equilibrium with W, D and R; or load = 0 and construct lost) depend upon the sex distortion *m* (0.5 [no sex bias] to 1 [only male progeny]) and the female W/D heterozygote fitness ($0 \le w_{{\mathrm{WD}},{\mathrm{xx}}} \le 1$). For low female fitness, adding an X-shredder (increasing sex distortion *m*) is predicted to beneficially increase the load on the population. For complete female heterozygous sterility (purple line), the drive construct disappears from the population if there is no sex distortion (*m* = 1/2), whereas sufficiently increasing the sex distortion allows the SDGD^dsx^ to spread and impose a load on the population. The presence of the X-shredder keeps the construct predominantly in males, therefore mitigating the heterozygous female sterility effect. By contrast if female fitness is sufficiently high (lines of fitness 0.5 or greater on plot), the load decreases at high sex distortion because due to male bias, SDGD^dsx^ males replace high-fitness W/D heterozygote females which decreases the ability of the construct to spread. For a complete male sex bias, *m* = 1, no W/D heterozygote females are created (and therefore no female/male SDGD^dsx^ homozygotes), since all X chromosomes are shredded and SDGD^dsx^ males have no female progeny; thus the load at *m* = 1 does not depend on the fitness of female W/D heterozygote individuals since none are present. Only SDGD^dsx^ heterozygous males can pass on the construct, with the SDGD^dsx^ allele present in reduced frequency in an intermediate equilibrium with wildtype and resistance alleles and a load less than one for these parameters. (In general, the amount of reduction in SDGD^dsx^ frequency and load at *m* = 1 will depend on parameters such as the homing rate, here less than 100%, the rate of resistant R mutation, and the relative fertility of the R and SDGD^dsx^ allele).

Supplementary Fig. 12 Comparison of the predicted equilibrium for release of SDGD^dsx^ or dsxF^CRISPRh^ into a wild-type population. (Top panel) Comparison of the predicted equilibrium load (that is, the reduction in reproductive output by the population after 400 generations) for release of SDGD^dsx^ or dsxF^CRISPRh^ into a wild-type population, varying the fitness cost to heterozygote W/D females. The discrete generation model predicts that the SDGD^dsx^ construct (blue solid line) is more robust to reductions in female heterozygote fitness compared to dsxF^CRISPRh^ (red line), still maintaining a substantial load even at 100% reduction in female heterozygote fitness (that is females heterozygous for the drive are completely non-viable). We also consider the possibility that the X-shredder component may be lost from the SDGD^dsx^ construct during homing (bottom panel, blue dashed line), such that out of the drive alleles transmitted from female or male W/D individuals, 0.01% will not have a functioning X-shredder component. For low to mid-fitness costs, the predicted load is similar to that of the dsxF^CRISPRh^ drive-only construct since the fraction of drive individuals without an intact X-shredder is high; for high fitness costs, the load merges with that of intact SDGD^dsx^ (blue solid line) since almost all drive individuals have an intact X-shredder. We use representative parameters for both constructs for comparison (drive transmission *d*_f_ = *d*_m_ = 0.95 for both males and females; rate of resistance *u*_f_ = *u*_m_ = 0.5; X-shredding parameter for SDGD^dsx^ is *m*=0.95; no reduction in fitness for heterozygote males).

Supplementary Fig. 13 Time dynamics of the frequency of SDGD^dsx^ transgenic individuals and relative egg output. Time dynamics of the frequency of SDGD^dsx^ transgenic individuals (solid lines) and relative eggs output (dotted lines) in the population as predicted by the deterministic discrete-generation model using experimental parameters given in Supp Table 2 and assuming two initial releases of 50% of SDGD heterozygote males and females (black line) or 10% SDGD heterozygous males only (grey line). Independently of the release scenarios, for these parameters, the frequency of transgenic individuals reaches an intermediate equilibrium while W, R and D alleles and the egg output is reduced (population suppressed).

### Supplementary information

Supplementary Information Supplementary Figs. 1–13, Tables 1–5 and Note

Reporting Summary

Supplementary Data

## Data Availability

The full sequence of vectors is provided through the NCBI database. The GenBank accession codes for vectors p172 and p182–244 are MT270142 and MT270141, respectively. Sanger sequencing of vector p182–244 is available as Supplementary Information. Additional vector sequences are provided as Supplementary Note.
