# Author Correction: A male-biased sex-distorter gene drive for the human malaria vector *Anopheles gambiae*

**DOI:** 10.1038/s41587-020-0658-1

**Published:** 2020-08-06

**Authors:** Alekos Simoni, Andrew M. Hammond, Andrea K. Beaghton, Roberto Galizi, Chrysanthi Taxiarchi, Kyros Kyrou, Dario Meacci, Matthew Gribble, Giulia Morselli, Austin Burt, Tony Nolan, Andrea Crisanti

**Affiliations:** 1 Department of Life Sciences, Imperial College London, London, UK; 2 Polo d’Innovazione Genomica, Genetica e Biologia, Terni, Italy; 3 W. Harry Feinstone Department of Molecular Microbiology and Immunology, Johns Hopkins University, Baltimore, MD USA; 4 Centre for Applied Entomology and Parasitology, School of Life Sciences, Keele University, Keele, UK; 5 Department of Life Sciences, Imperial College London, Silwood Park, Ascot, UK; 6 Liverpool School of Tropical Medicine, Liverpool, UK; 7 Department of Molecular Medicine, University of Padova, Padova, Italy

**Keywords:** Molecular engineering, Genetics, Gene regulation

Correction to: *Nature Biotechnology* 10.1038/s41587-020-0508-1, published online 11 May 2020.

In the version of this article initially published online, a plot from a different dataset was substituted for the left panel of Fig. 2b subsequent to peer review. The conclusions of the study are unaffected. The error has been corrected in the print, PDF and HTML versions of the article.

**Fig. 2:**
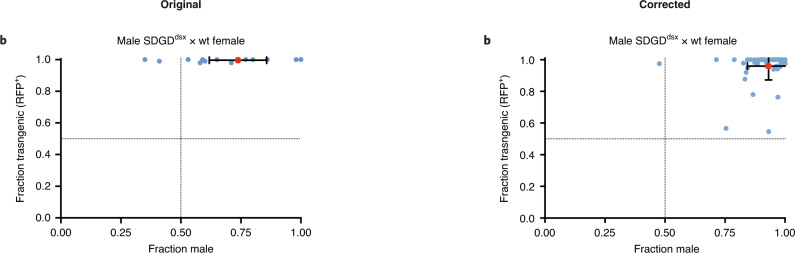
Original and corrected. Left panel, Fig. 2b.

