# Supplementary figures and images for: A male-biased sex-distorter gene drive for the human malaria vector Anopheles gambiae
Source: Nat Biotechnol. 2020 May 11;38(9):1054–60. doi: 10.1038/s41587-020-0508-1 (PMC7473848; doi:10.1038/s41587-020-0508-1)

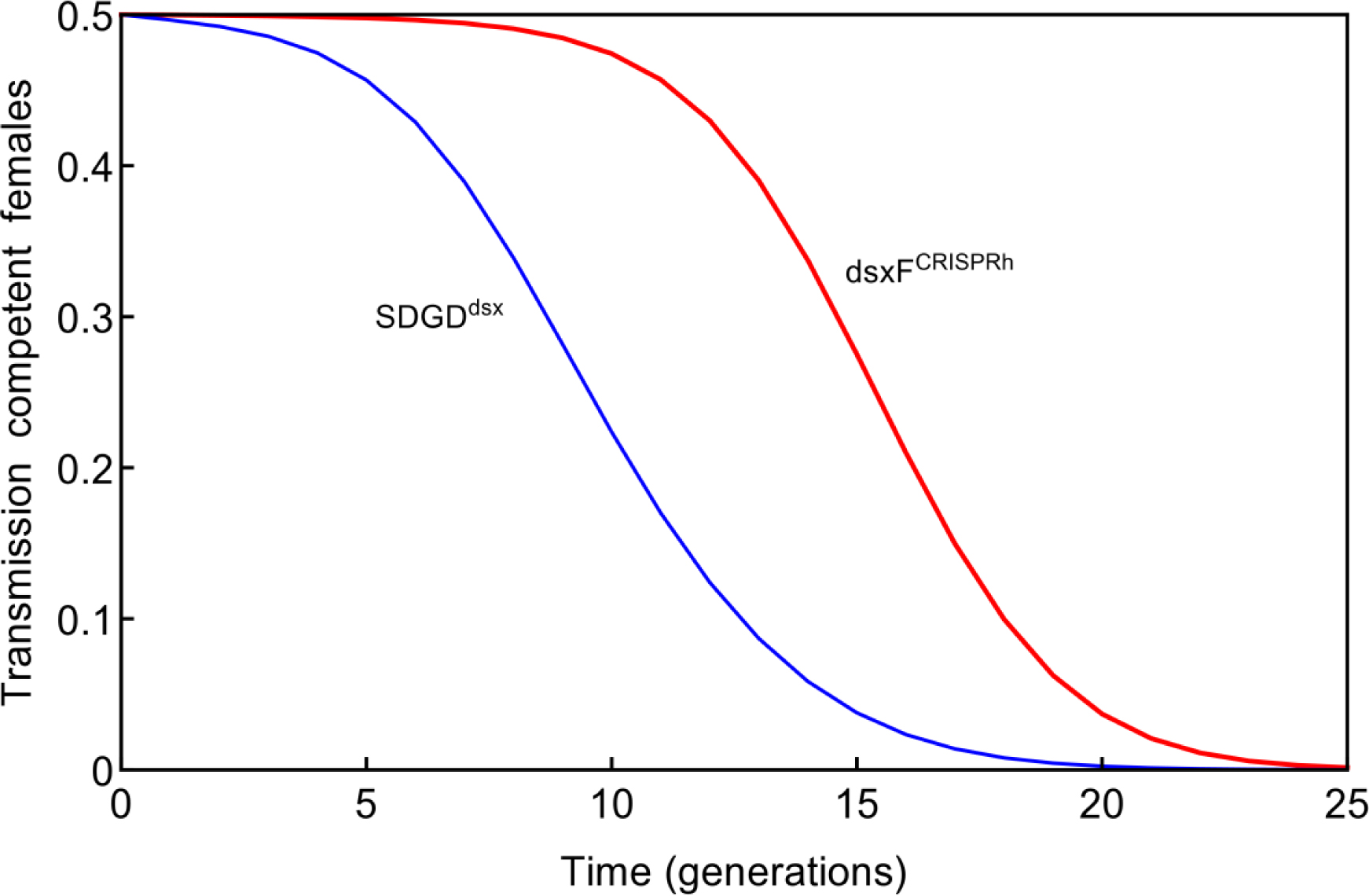

Supplement: Model prediction of the reduction in abundance of transmission competent females. — Model prediction of the reduction over time in abundance of transmission competent (that is biting) females, normalised by the initial total population size, for the SDGDdsx construct compared to dsxFCRISPRh (Kyrou et al., 2018), using a continuous-time population dynamics model (see Supp. modeling Methods) for ‘field' release of 1% heterozygous transgenic males in the male population. The SDGDdsx construct is predicted to suppress the population of transmission competent females faster than the dsxFCRISPRh, mainly due to the creation of a male bias in the population by the sex distorter. Parameters used for SDGDdsx are in Table S2; dsxFCRISPRh parameters were estimated from Kyrou et al. (2018) using an average W/D female fitness of 0.4335; for both, Rm (intrinsic growth rate per generation) = 6. At long times (not shown), the SDGDdsx population rebounds to an intermediate equilibrium (suppressed) population. [file 41587_2020_508_Fig4_ESM.jpg]

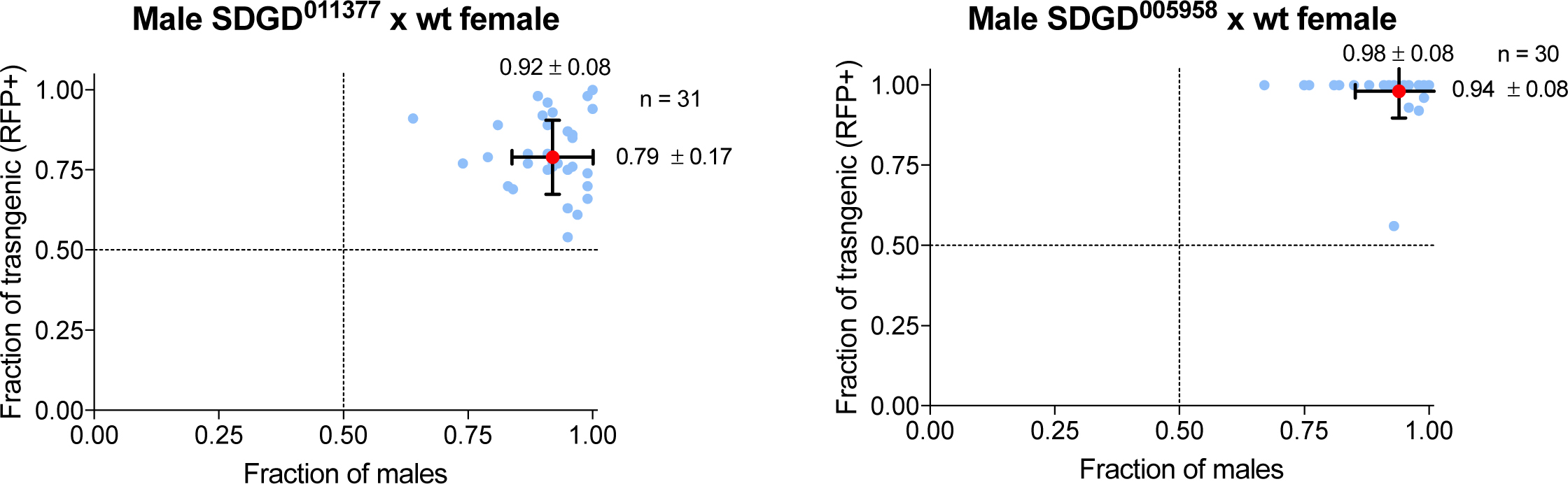

Supplement: Sex and inheritance bias caused by SDGD011377 and SDGD005958 males. — Scattered plots showing the fraction of transgene inheritance (y-axis) against male bias (x-axis) in the progeny of heterozygous male SDGD011377 (left-hand panel) SDGD005958 and (right-hand panel) crossed to wild type females. Individual coloured dots represent the progeny derived from a single female and the red dots indicate the average of the population (with respective values indicated next to the plot ± s.e.m.). Error bars indicate standard deviation. SDGD at both loci showed a high transmission rate of the transgene determined by scoring in the progeny the presence of RFP marker that is linked to the SDGD allele. The progeny of SDGD/+ at both loci showed a strong sex ratio distortion towards males. Dotted lines indicate expected Mendelian inheritance. [file 41587_2020_508_Fig5_ESM.jpg]

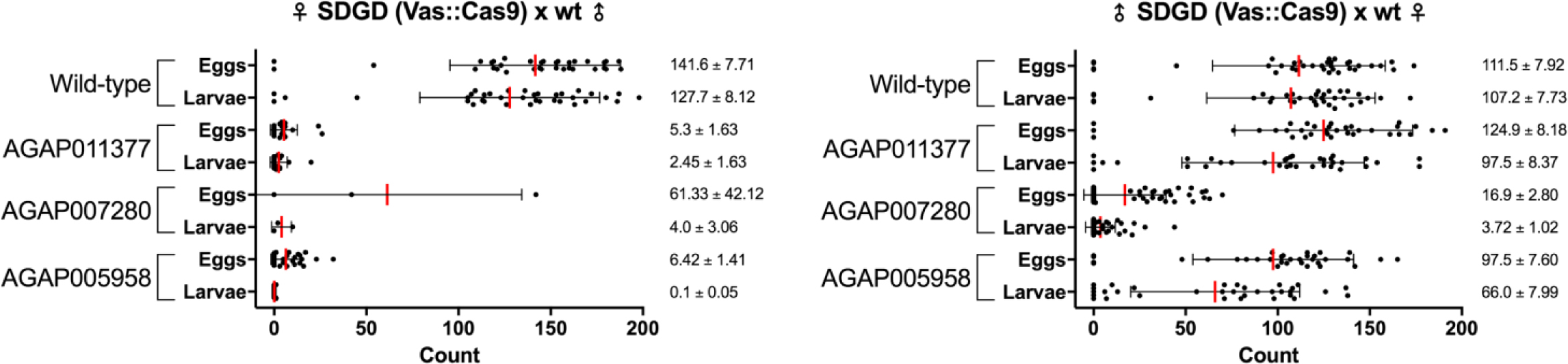

Supplement: Fecundity phenotype of SDGD targeting 3 different fertility loci in An. gambiae. — SDGD constructs expressing the Cas9 nuclease under the control of the Vas2 promoter were generated targeting the fertility loci AGAP011377, AGAP007280 and AGAP005859 (as indicated). SDGD heterozygous male and female were crossed to wild-type counterparts. Each dot represents progeny of individual females. Fecundity was measured by counting the number of eggs per female and the hatched larvae. Values on the right represent average ± s.e.m. A strong fertility effect was observed in heterozygous SDGD females at 3 loci, while male fecundity was strongly impaired by targeting 7280 and 5859 loci. Vertical red bars indicate average count, and error bars indicate standard deviation. A minimum of 20 females were analysed for each cross. [file 41587_2020_508_Fig6_ESM.jpg]

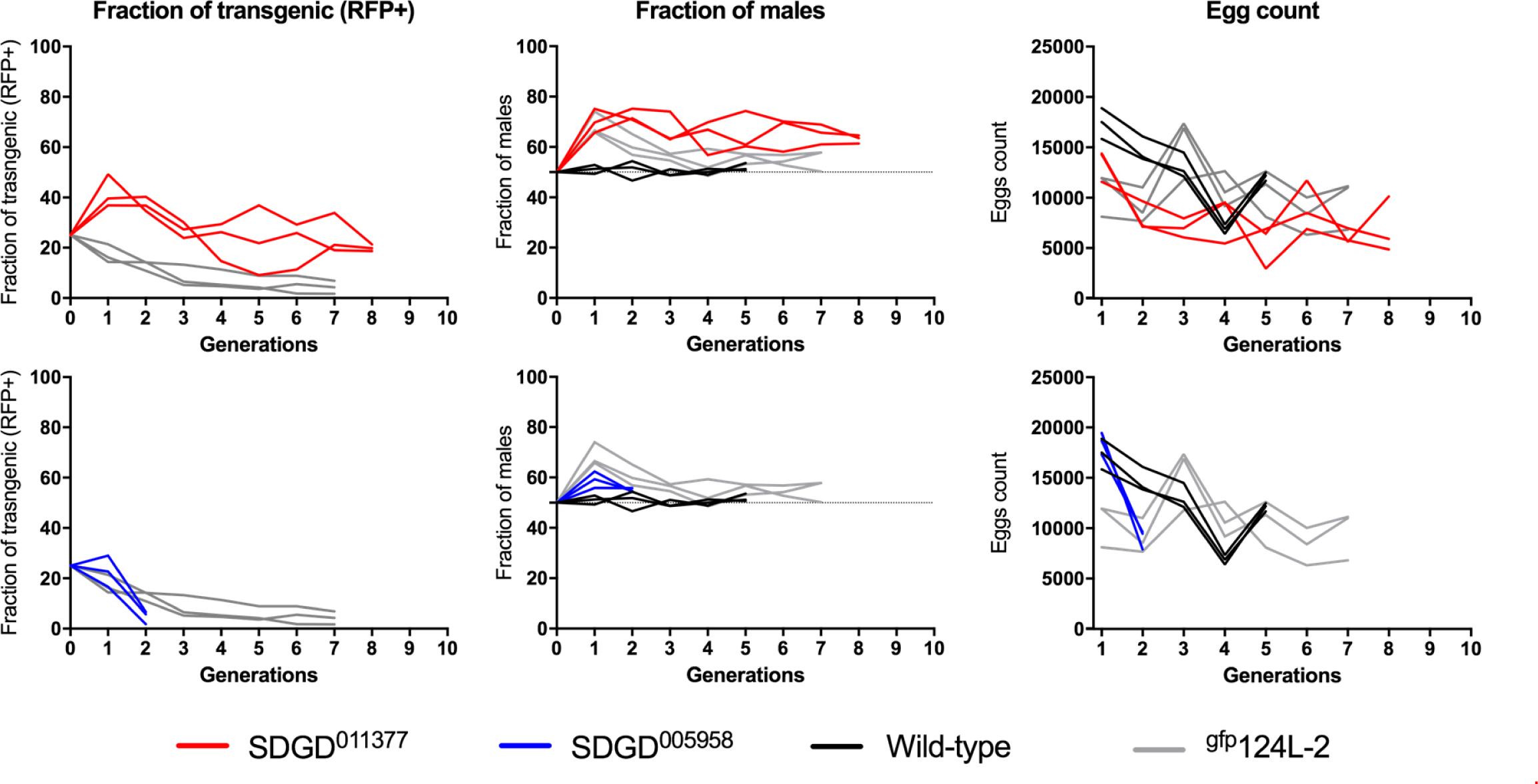

Supplement: Kinetics of SDGD011377 and SDGD005958 spread in target mosquito populations. — In these experiments 100 heterozygous transgenic males were introduced into a population of 100 wild-type males and 200 wild-type females (transgenic allele frequency of 12.5%). The frequency of the transgene was monitored every generation together with the fraction of males in the population and the total number of eggs laid. Each consecutive generation was established by collecting 450 eggs. The frequency of the SDGD011377 (red lines) and SDGD005958 (blue lines) was compared to that of the autosomal self-limiting sex-distorter gfp124L-2 (grey lines) (Galizi et al., 2014) as well as to that of wild-type populations (black lines) as control. Each genotype was tested in triplicate cages. The SDGD005958 allele disappeared from the populations at generation 2 due to the strong fertility effects. The SDGD011377 alleles persisted in the populations despite the fertility effects but failed to increase over the frequency of release on subsequent generations. The fraction of males in the population was stably biased to about 65%. [file 41587_2020_508_Fig7_ESM.jpg]

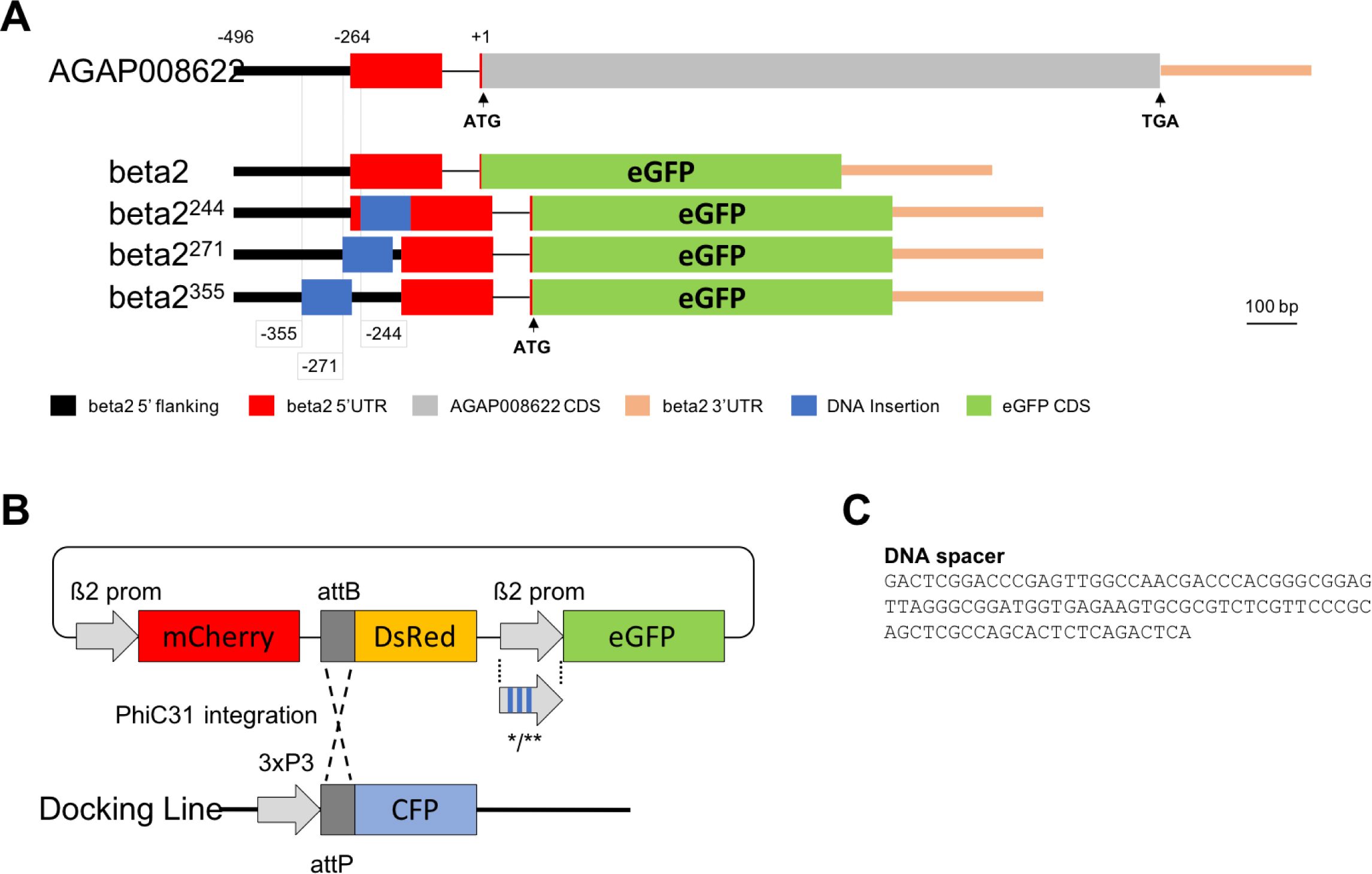

Supplement: Mutagenesis of the beta2 tubulin promoter. — (a) Schematic overview of the AGAP008622 (beta2-tubulin) locus and the three beta2 promoter variants generated by placing a 100bp GC-rich DNA sequence (blue rectangle) upstream of the start codon at position 244, 271 and 355. (b) The double-fluorescence reporter assay developed to detect the effects of the beta2 promoter modifications on its transcription level. A construct for each modification was generated harbouring the modified beta2 promoter (stars key) driving an eGFP marker, while a second, unmodified, beta2 promoter was driving a mCherry protein. The constructs were integrated within the same autosomal docking line by PhiC31-mediated integration by replacing a 3xP3::CFP cassette with a 3xP3::DsRed as integration marker. C) The sequence of 100bp GC-rich DNA region inserted at the 5’ of the beta2 promoter. [file 41587_2020_508_Fig8_ESM.jpg]

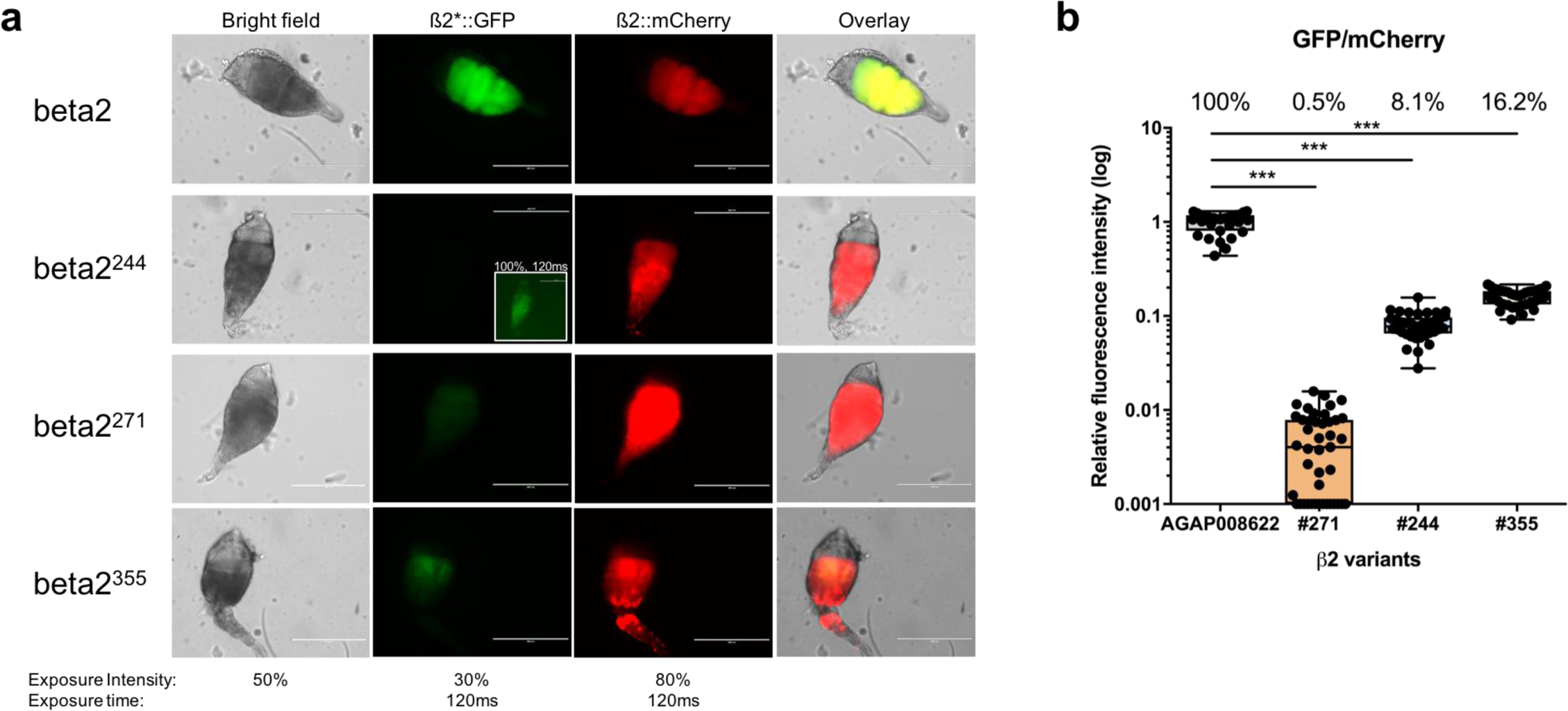

Supplement: GFP and mCherry signal quantification from mosquito testes transformed with modified beta2 promoters. — (a) Dissected testis from beta2wt, beta2271, beta2244 and beta2355 transgenic lines containing a single heterozygous insertion were microphotographed under the same exposure settings (as indicated), using mCherry as internal control. Line beta2271 showed GFP fluorescence intensity comparable to background; GFP expression was detectable at increased exposure (100% gain, 120ms, inset). Scale bar, 200 µm. (b) Quantification of fluorescence intensity as GFP/mCherry ratio, normalized to the beta2wt control (100%). Average relative intensity is indicated above the bars. *** indicates P value < 0.001 (ordinary One-way ANOVA). A minimum of 31 testes were analysed from individual expressing each promoter variant. [file 41587_2020_508_Fig9_ESM.jpg]

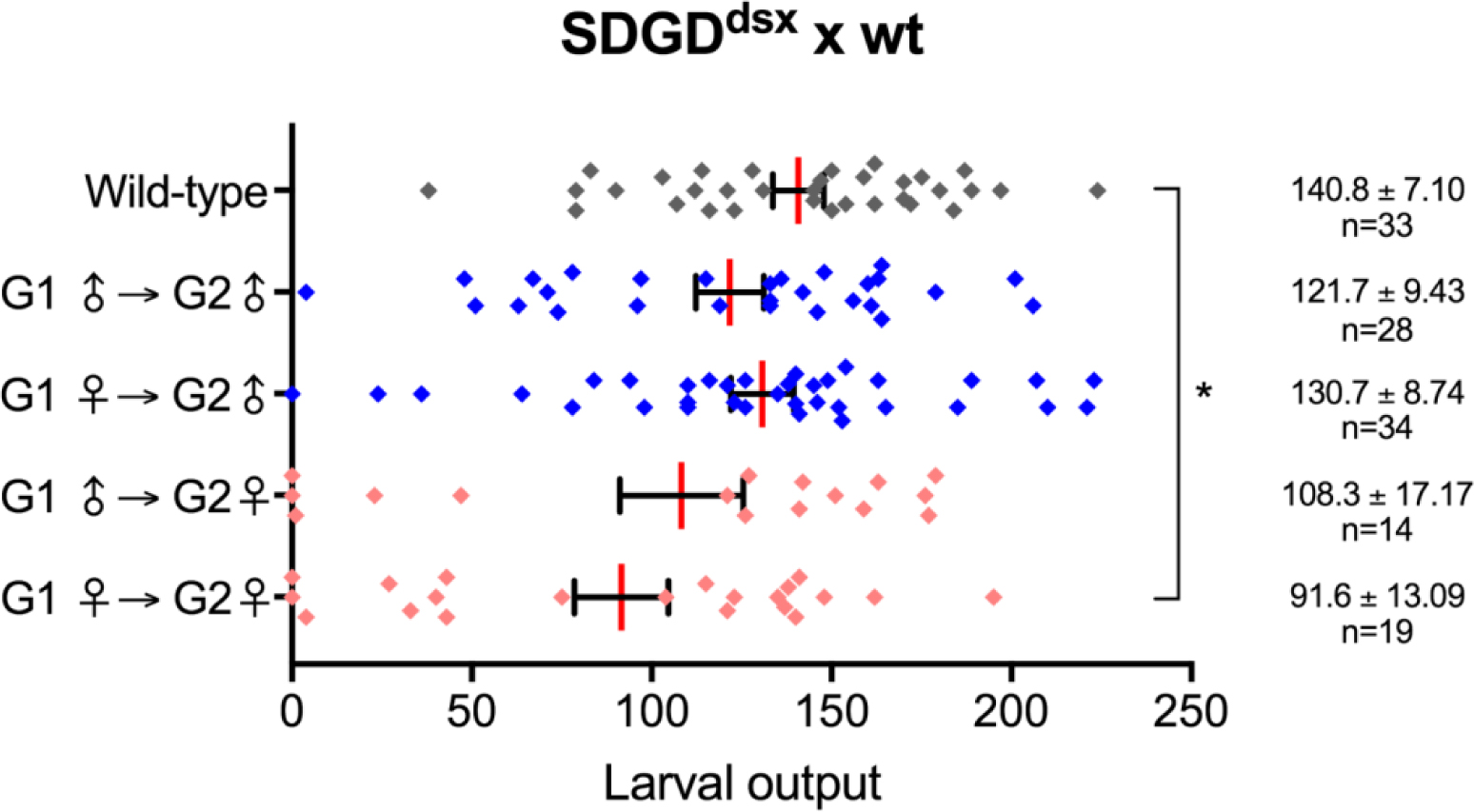

Supplement: Maternal or paternal contribution to the fecundity of the SDGDdsx allele. — Male and female SDGDdsx heterozygotes that had inherited a maternal or paternal copy of the SDGDdsx allele were crossed to wild type counterparts and assessed for fecundity. The total larval output is plotted for individual females (dots). Red bars indicate the average and the mean count (± s.e.m.) is shown. Females inheriting the transgene from the mother (G1♀ → G2♀) have significantly lower larval progeny (*P = 0.0256, Kruskal-Wallis test) compared to wild-type control. [file 41587_2020_508_Fig10_ESM.jpg]

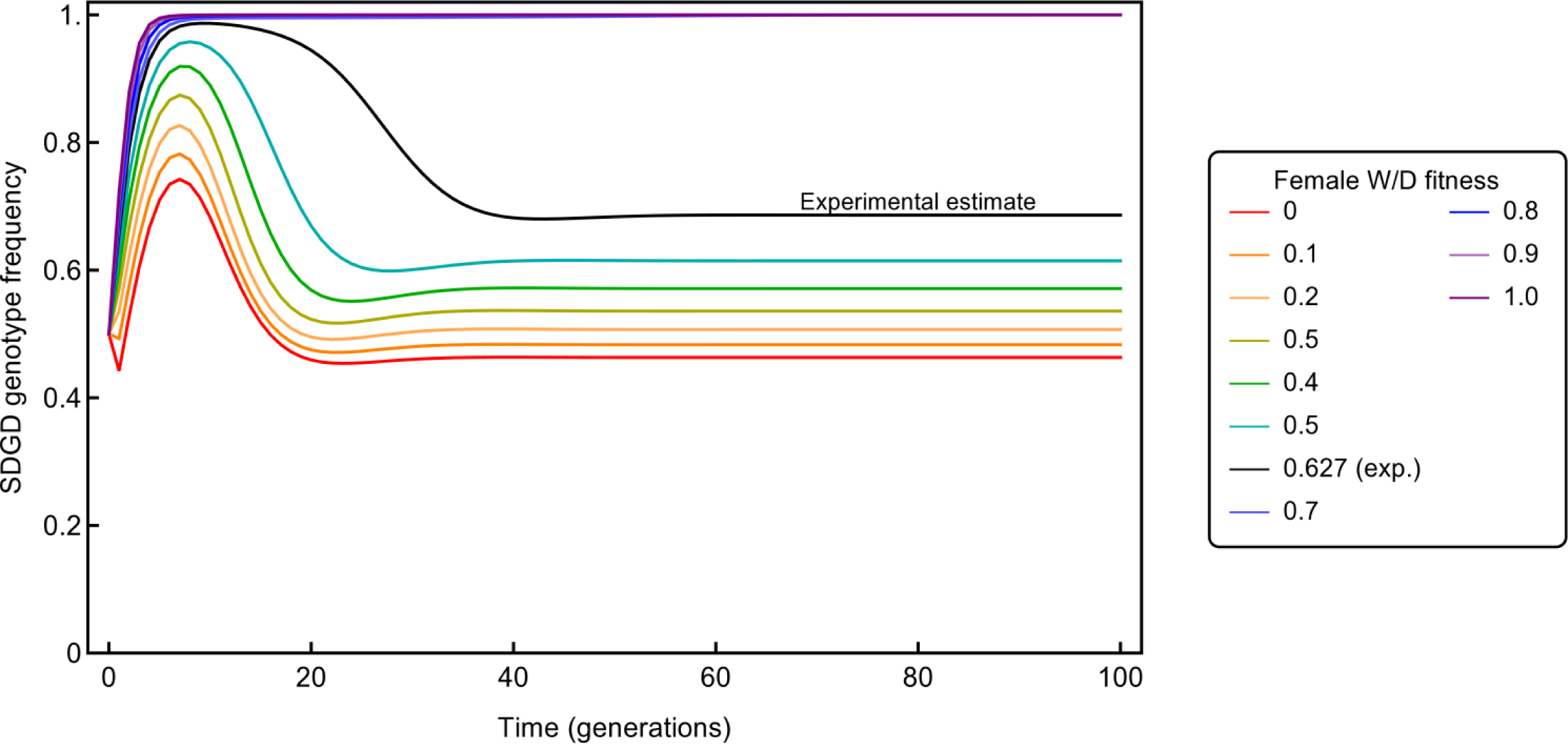

Supplement: Time dynamics of the frequency of SDGDdsx as function of female fitness. — Time dynamics of the frequency of SDGDdsx transgenic individuals in the population as a function of W/D (where D represent the SDGD allele and W the wild-type allele) female fitness (wWD,xx), as predicted by the deterministic discrete-generation model at 25% initial allelic frequency. The graph shows the frequency of SDGD heterozygote males and females as a proportion of the male (or female) population, with other parameter estimates and baseline values given in Supp Table 2 (SDGD male fitness = 0.854; m (sex distortion) = 0.93). The predicted outcome at high W/D female fitness is elimination of the population, and at lower fitness, an intermediate equilibrium with W, R and D alleles. The result (black line) for the experimental estimate for female SDGD heterozygote fitness, wWD,xx = 0.627, is in a parameter region where even a small (positive) change leads to a prediction of population elimination instead of suppression. [file 41587_2020_508_Fig11_ESM.jpg]

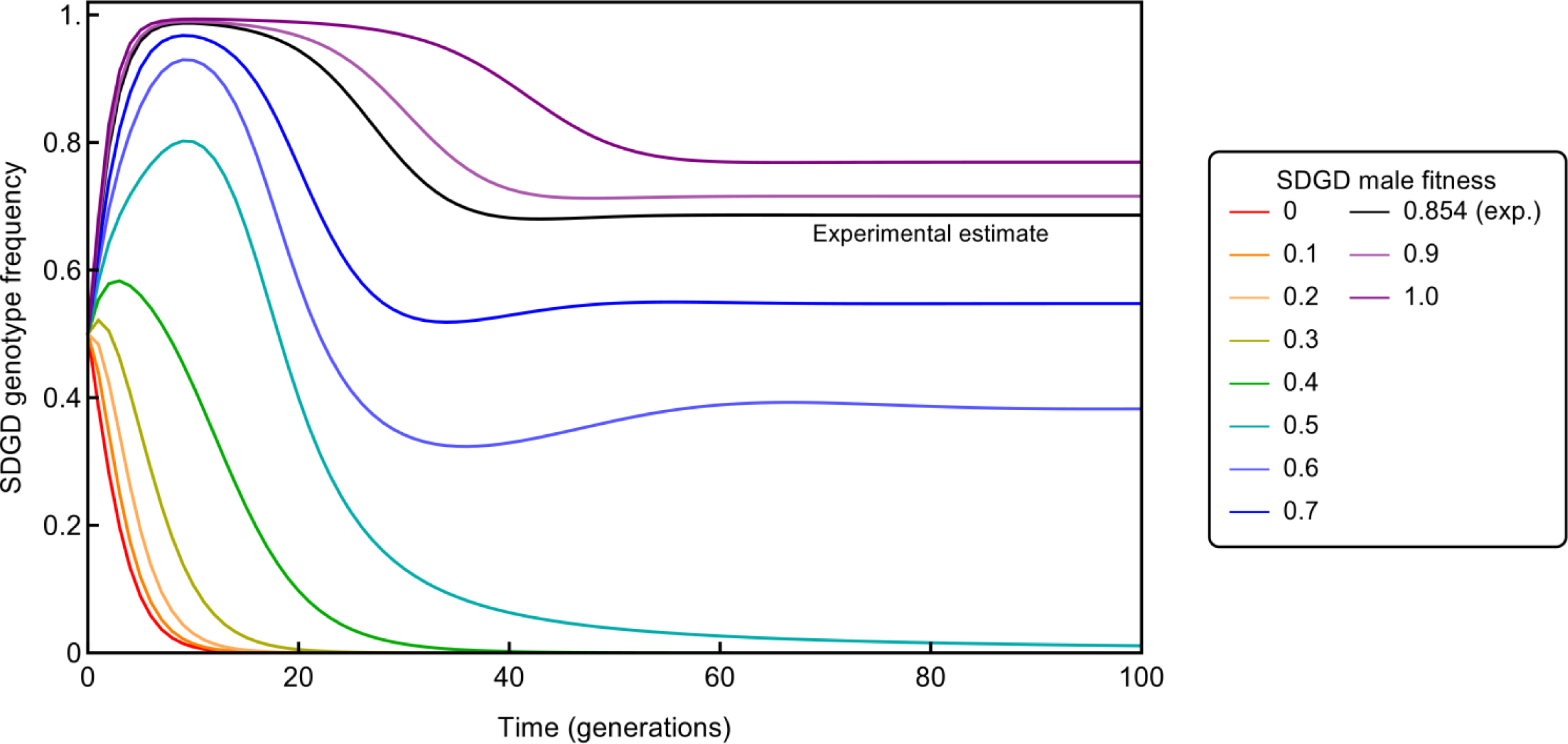

Supplement: Time dynamics of the frequency of SDGDdsx as function of male fitness. — Time dynamics of the frequency of SDGDdsx transgenic individuals in the population as a function of the fitness of SDGD males (assume wWD,xY = wDR,xY = wDD,xY) as predicted by the deterministic discrete-generation model. Initial release is 50% of SDGD heterozygote males and females as a proportion of the male (or female) population, with other parameter estimates and baseline values given in Supp Table 2 (W/D female fitness wWD,xx = 0.627; m = 0.93). For low SDGD male fitness (<≈0.5), the construct is eventually lost. [file 41587_2020_508_Fig12_ESM.jpg]

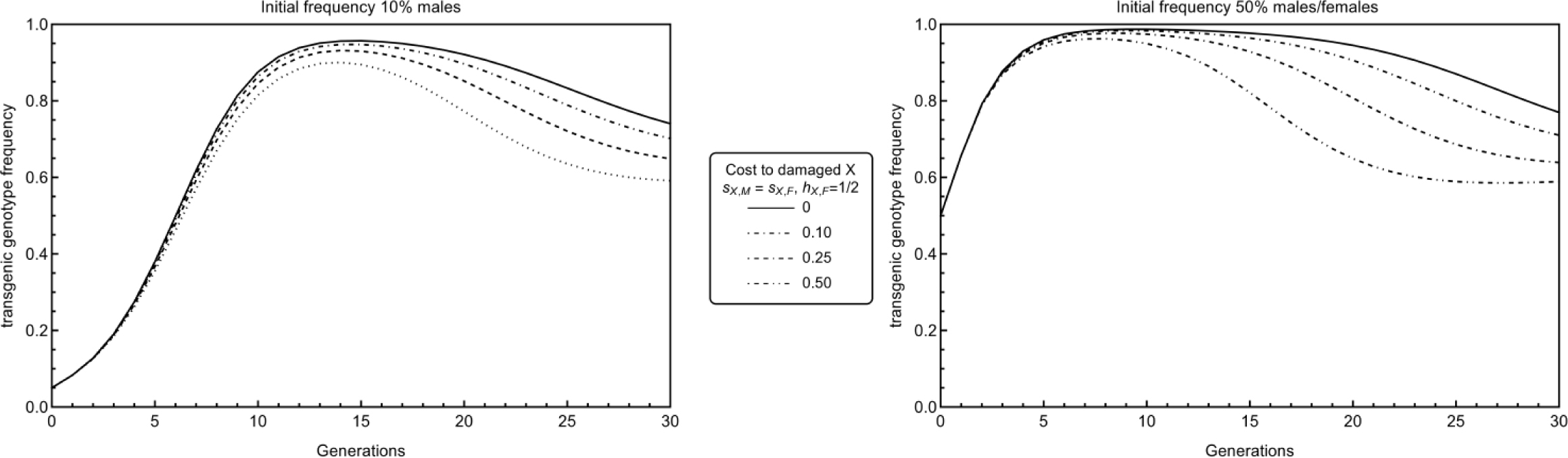

Supplement: Impact of fitness due to damaged X chromosome. — modeling the impact of fitness reduction, due to the inheritance of damaged X chromosomes passed through X-shredding in a SDGD male, on the spread of the SDGD transgenics as predicted by the deterministic discrete-generation cage model. For simplicity, we assume that the additional cost to carrying one damaged X chromosome in males is the same as the cost in females that carry two copies of the damaged X (sX,f = sX,m), and females with one damaged X and one wildtype X chromosome have only half the fitness cost of females with two copies (dominance coefficient hX,f = 1/2). Estimates used for other parameters given in Supplementary Table 2. [file 41587_2020_508_Fig13_ESM.jpg]

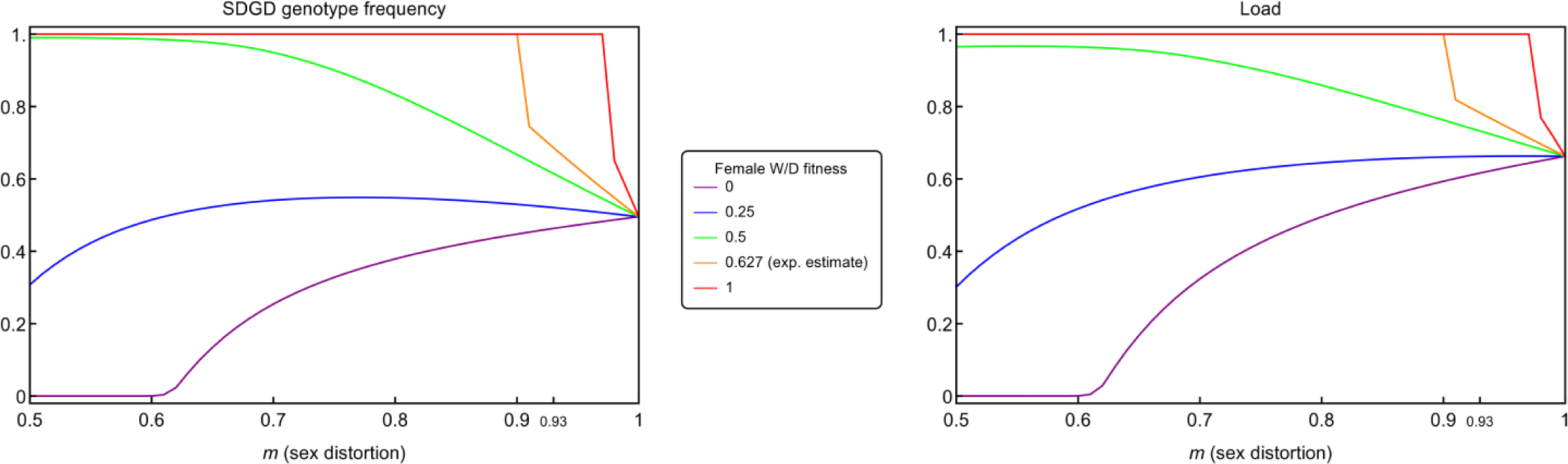

Supplement: Effect of female fitness on SDGD frequency and population load. — Effect of female W/D heterozygote fitness (D refers to the SDGDdsx allele) on the SDGD genotype frequency (that is, individuals with at least one copy of the SDGD) and load after 200 generations, as predicted by the deterministic discrete-generation model. Parameter estimates and baseline values given in Supp Table 2 (SDGD male fitness = 0.854). The possible outcomes (load = 1 and population elimination; intermediate equilibrium with W, D and R; or load = 0 and construct lost) depend upon the sex distortion m (0.5 [no sex bias] to 1 [only male progeny]) and the female W/D heterozygote fitness ($0 \le w_{{\mathrm{WD}},{\mathrm{xx}}} \le 1$0≤wWD,xx≤1). For low female fitness, adding an X-shredder (increasing sex distortion m) is predicted to beneficially increase the load on the population. For complete female heterozygous sterility (purple line), the drive construct disappears from the population if there is no sex distortion (m = 1/2), whereas sufficiently increasing the sex distortion allows the SDGDdsx to spread and impose a load on the population. The presence of the X-shredder keeps the construct predominantly in males, therefore mitigating the heterozygous female sterility effect. By contrast if female fitness is sufficiently high (lines of fitness 0.5 or greater on plot), the load decreases at high sex distortion because due to male bias, SDGDdsx males replace high-fitness W/D heterozygote females which decreases the ability of the construct to spread. For a complete male sex bias, m = 1, no W/D heterozygote females are created (and therefore no female/male SDGDdsx homozygotes), since all X chromosomes are shredded and SDGDdsx males have no female progeny; thus the load at m = 1 does not depend on the fitness of female W [file 41587_2020_508_Fig14_ESM.jpg]

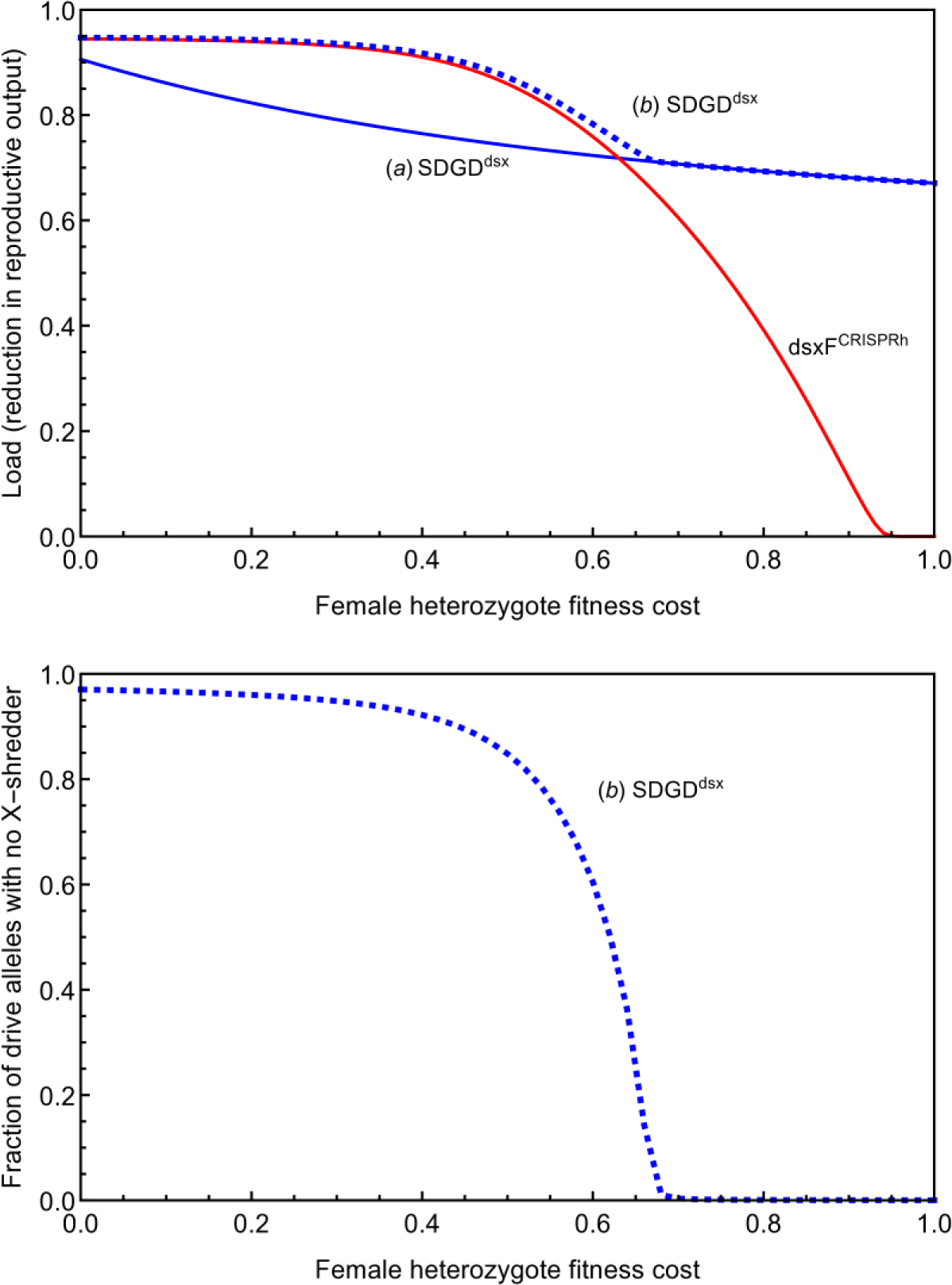

Supplement: Comparison of the predicted equilibrium for release of SDGDdsx or dsxFCRISPRh into a wild-type population. — (Top panel) Comparison of the predicted equilibrium load (that is, the reduction in reproductive output by the population after 400 generations) for release of SDGDdsx or dsxFCRISPRh into a wild-type population, varying the fitness cost to heterozygote W/D females. The discrete generation model predicts that the SDGDdsx construct (blue solid line) is more robust to reductions in female heterozygote fitness compared to dsxFCRISPRh (red line), still maintaining a substantial load even at 100% reduction in female heterozygote fitness (that is females heterozygous for the drive are completely non-viable). We also consider the possibility that the X-shredder component may be lost from the SDGDdsx construct during homing (bottom panel, blue dashed line), such that out of the drive alleles transmitted from female or male W/D individuals, 0.01% will not have a functioning X-shredder component. For low to mid-fitness costs, the predicted load is similar to that of the dsxFCRISPRh drive-only construct since the fraction of drive individuals without an intact X-shredder is high; for high fitness costs, the load merges with that of intact SDGDdsx (blue solid line) since almost all drive individuals have an intact X-shredder. We use representative parameters for both constructs for comparison (drive transmission df = dm = 0.95 for both males and females; rate of resistance uf = um = 0.5; X-shredding parameter for SDGDdsx is m=0.95; no reduction in fitness for heterozygote males). [file 41587_2020_508_Fig15_ESM.jpg]

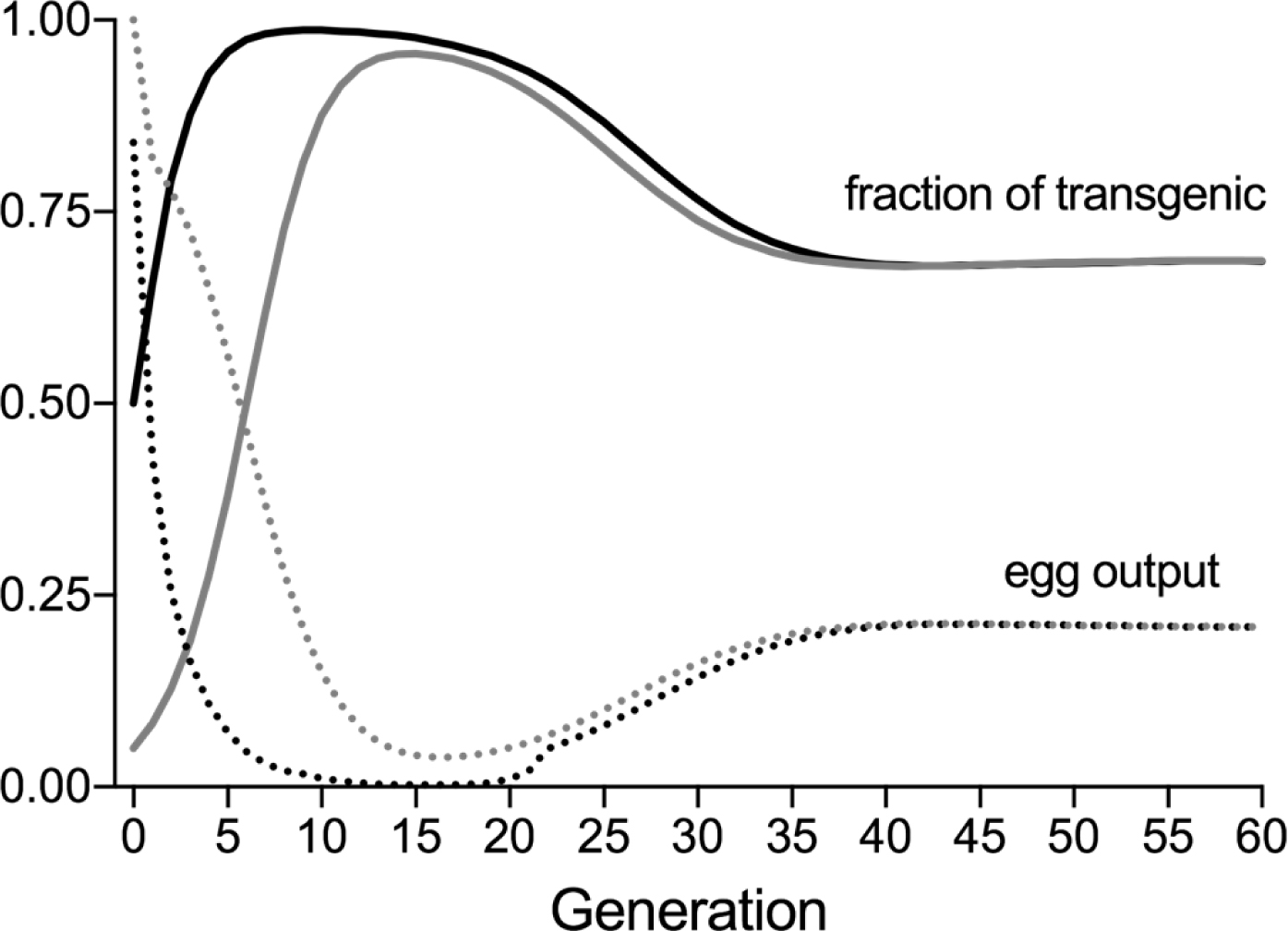

Supplement: Time dynamics of the frequency of SDGDdsx transgenic individuals and relative egg output. — Time dynamics of the frequency of SDGDdsx transgenic individuals (solid lines) and relative eggs output (dotted lines) in the population as predicted by the deterministic discrete-generation model using experimental parameters given in Supp Table 2 and assuming two initial releases of 50% of SDGD heterozygote males and females (black line) or 10% SDGD heterozygous males only (grey line). Independently of the release scenarios, for these parameters, the frequency of transgenic individuals reaches an intermediate equilibrium while W, R and D alleles and the egg output is reduced (population suppressed). [file 41587_2020_508_Fig16_ESM.jpg]
