# Supplementary material for: A male-biased sex-distorter gene drive for the human malaria vector Anopheles gambiae
Source: Nat Biotechnol. 2020 May 11;38(9):1054–60. doi: 10.1038/s41587-020-0508-1 (PMC7473848; doi:10.1038/s41587-020-0508-1)
Supplement: Supplementary file 14 — Supplementary Figs. 1–13, Tables 1–5 and Note [file 41587_2020_508_MOESM1_ESM.pdf]

nature biotechnology

SUPPLEMENTARY INFORMATION

https://doi.org/10.1038/s41587-020-0508-1

In the format provided by the authors and unedited.

A male-biased sex-distorter gene drive for the human malaria vector *Anopheles gambiae*

In the format provided by the authors and unedited

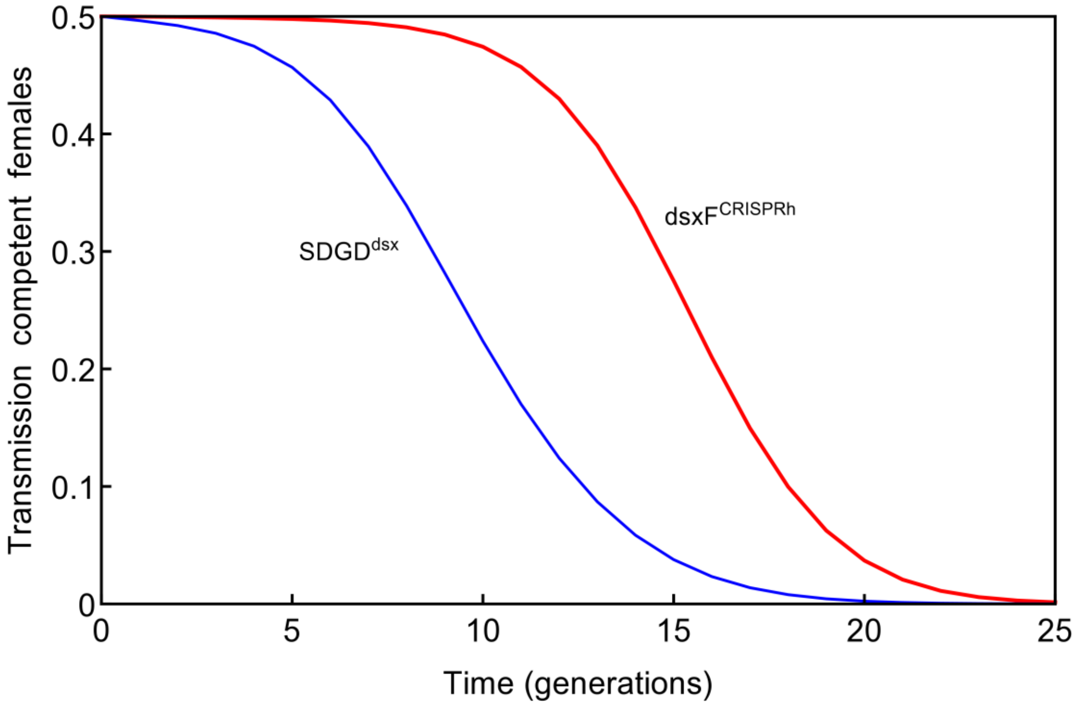

Supplementary Figure 1

Model prediction of the reduction in abundance of transmission competent females.

Model prediction of the reduction over time in abundance of transmission competent (i.e. biting) females, normalised by the initial total population size, for the SDGD^{dsx} construct compared to dsxF^{CRISPRh} (Kyrou et al., 2018), using a continuous-time population dynamics model (see Supp. Modelling Methods) for 'field' release of 1% heterozygous transgenic males in the male population. The SDGD^{dsx} construct is predicted to suppress the population of transmission competent females faster than the dsxF^{CRISPRh}, mainly due to the creation of a male bias in the population by the sex distorter. Parameters used for SDGD^{dsx} are in Table S2; dsxF^{CRISPRh} parameters were estimated from Kyrou et al. (2018) using an average W/D female fitness of 0.4335; for both, R_m (intrinsic growth rate per generation) = 6. At long times (not shown), the SDGD^{dsx} population rebounds to an intermediate equilibrium (suppressed) population.

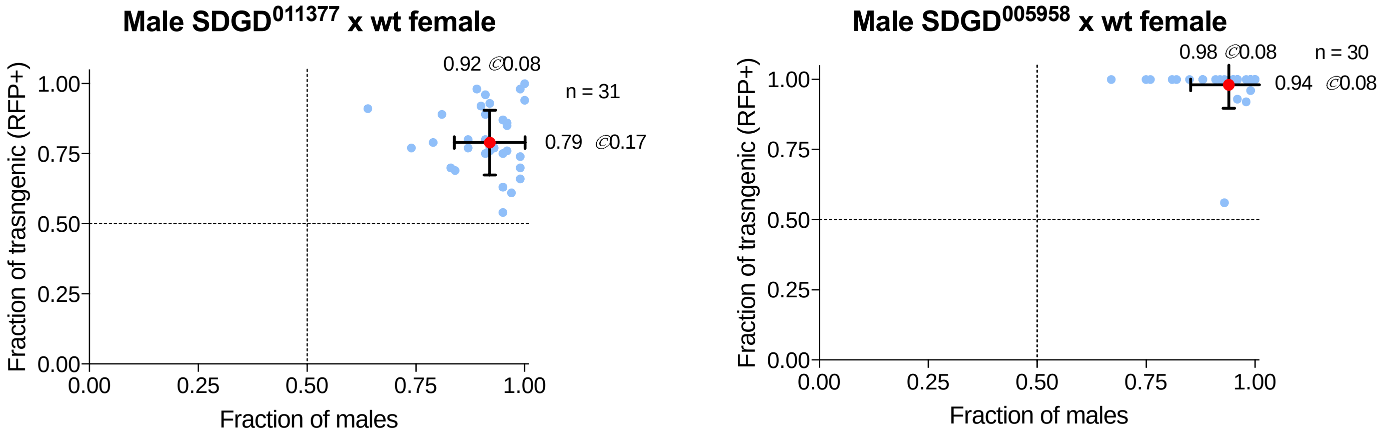

Supplementary Figure 2

Sex and inheritance bias caused by SDGD^{011377} and SDGD^{005958} males.

Scattered plots showing the fraction of transgene inheritance (y-axis) against male bias (x-axis) in the progeny of heterozygous male SDGD^{011377} (left-hand panel) SDGD^{005958} and (right-hand panel) crossed to wild type females. Individual coloured dots represent the progeny derived from a single female and the red dots indicate the average of the population (with respective values indicated next to the plot ± s.e.m.). Error bars indicate standard deviation. SDGD at both loci showed a high transmission rate of the transgene determined by scoring in the progeny the presence of RFP marker that is linked to the SDGD allele. The progeny of SDGD/+ at both loci showed a strong sex ratio distortion towards males. Dotted lines indicate expected Mendelian inheritance.

.

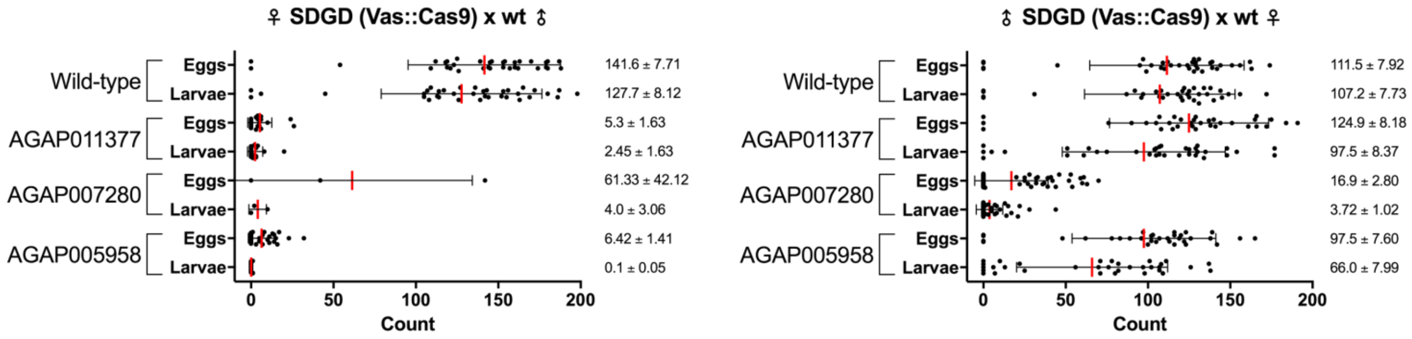

Supplementary Figure 3

Fecundity phenotype of SDGD targeting 3 different fertility loci in *An. gambiae*

SDGD constructs expressing the Cas9 nuclease under the control of the *Vas2* promoter were generated targeting the fertility loci *AGAP011377*, *AGAP007280* and *AGAP005859* (as indicated). SDGD heterozygous male and female were crossed to wild-type counterparts. Each dot represents progeny of individual females. Fecundity was measured by counting the number of eggs per female and the hatched larvae. Values on the right represent average ± s.e.m. A strong fertility effect was observed in heterozygous SDGD females at 3 loci, while male fecundity was strongly impaired by targeting 7280 and 5859 loci. Vertical red bars indicate average count, and error bars indicate standard deviation. A minimum of 20 females were analysed for each cross.

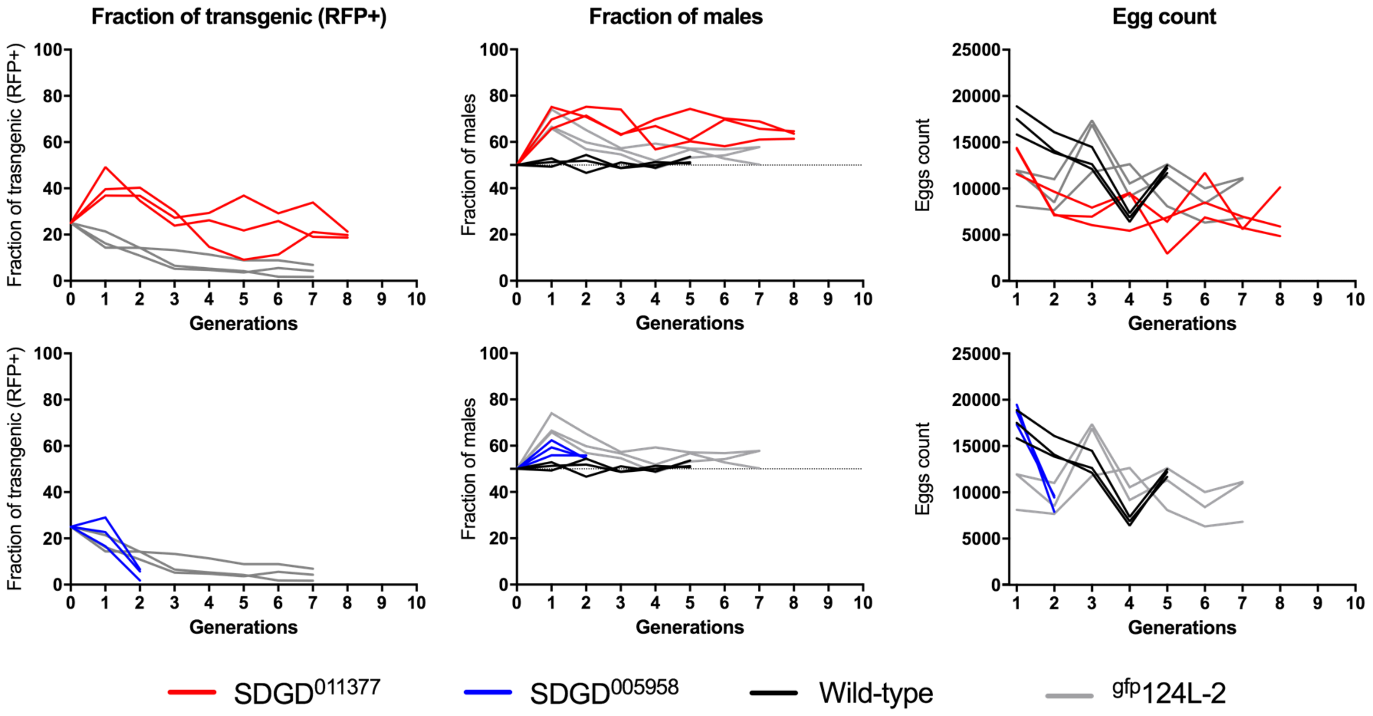

Supplementary Figure 4

Kinetics of SDGD^{011377} and SDGD^{005958} spread in target mosquito populations.

In these experiments 100 heterozygous transgenic males were introduced into a population of 100 wild-type males and 200 wild-type females (transgenic allele frequency of 12.5%). The frequency of the transgene was monitored every generation together with the fraction of males in the population and the total number of eggs laid. Each consecutive generation was established by collecting 450 eggs. The frequency of the SDGD^{011377} (red lines) and SDGD^{005958} (blue lines) was compared to that of the autosomal self-limiting sex-distorter ^{gfp}124L-2 (grey lines) (Galizi et al., 2014) as well as to that of wild-type populations (black lines) as control. Each genotype was tested in triplicate cages. The SDGD^{005958} allele disappeared from the populations at generation 2 due to the strong fertility effects. The SDGD^{011377} alleles persisted in the populations despite the fertility effects but failed to increase over the frequency of release on subsequent generations. The fraction of males in the population was stably biased to about 65%.

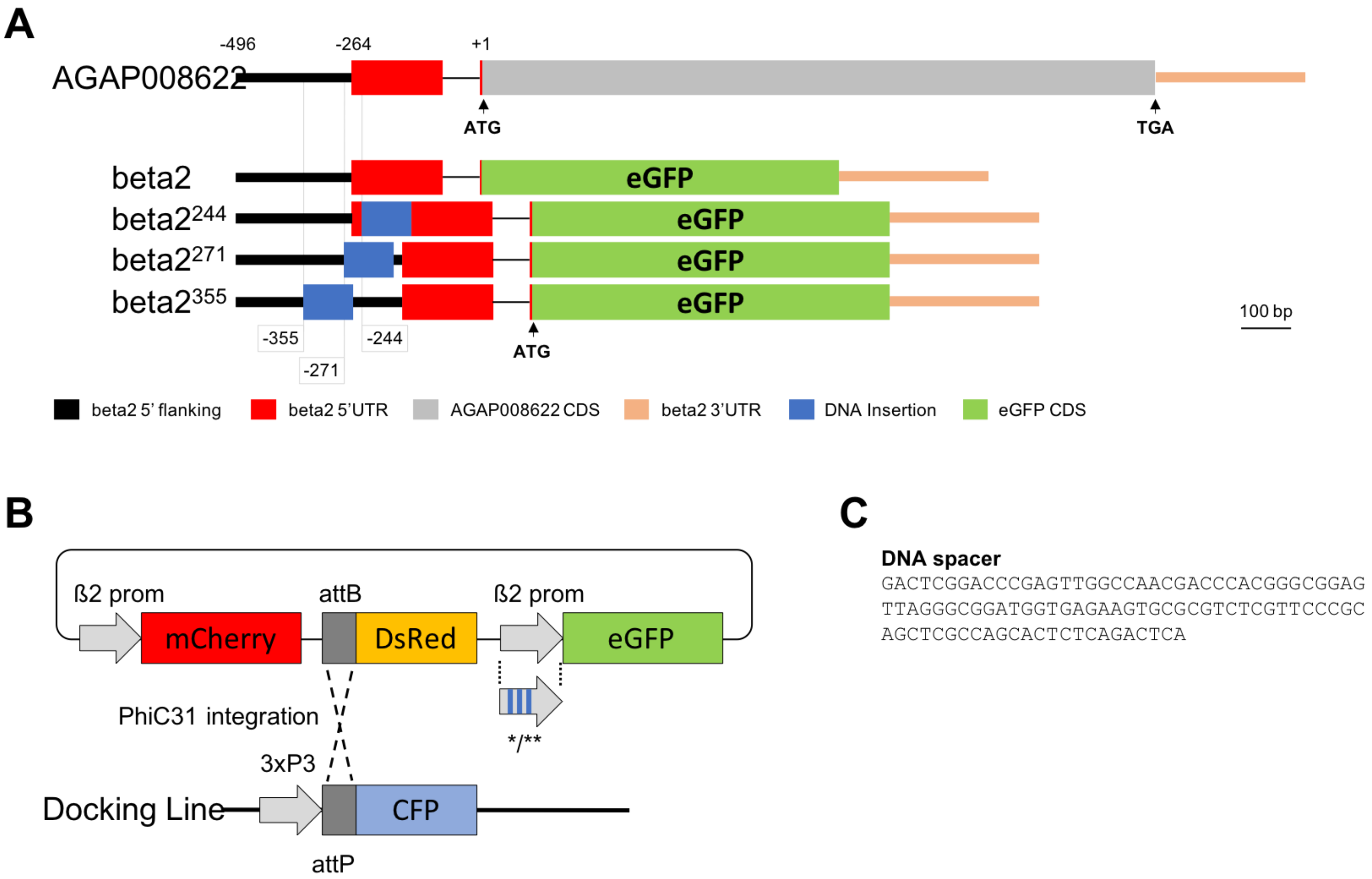

Supplementary Figure 5

Mutagenesis of the beta2 tubulin promoter.

A) Schematic overview of the *AGAP008622* (*beta2-tubulin*) locus and the three *beta2* promoter variants generated by placing a 100bp GC-rich DNA sequence (blue rectangle) upstream of the start codon at position 244, 271 and 355. B) The double-fluorescence reporter assay developed to detect the effects of the *beta2* promoter modifications on its transcription level. A construct for each modification was generated harbouring the modified *beta2* promoter (stars key) driving an eGFP marker, while a second, unmodified, *beta2* promoter was driving a mCherry protein. The constructs were integrated within the same autosomal docking line by PhiC31-mediated integration by replacing a 3xP3::CFP cassette with a 3xP3::DsRed as integration marker. C) The sequence of 100bp GC-rich DNA region inserted at the 5' of the *beta2* promoter.

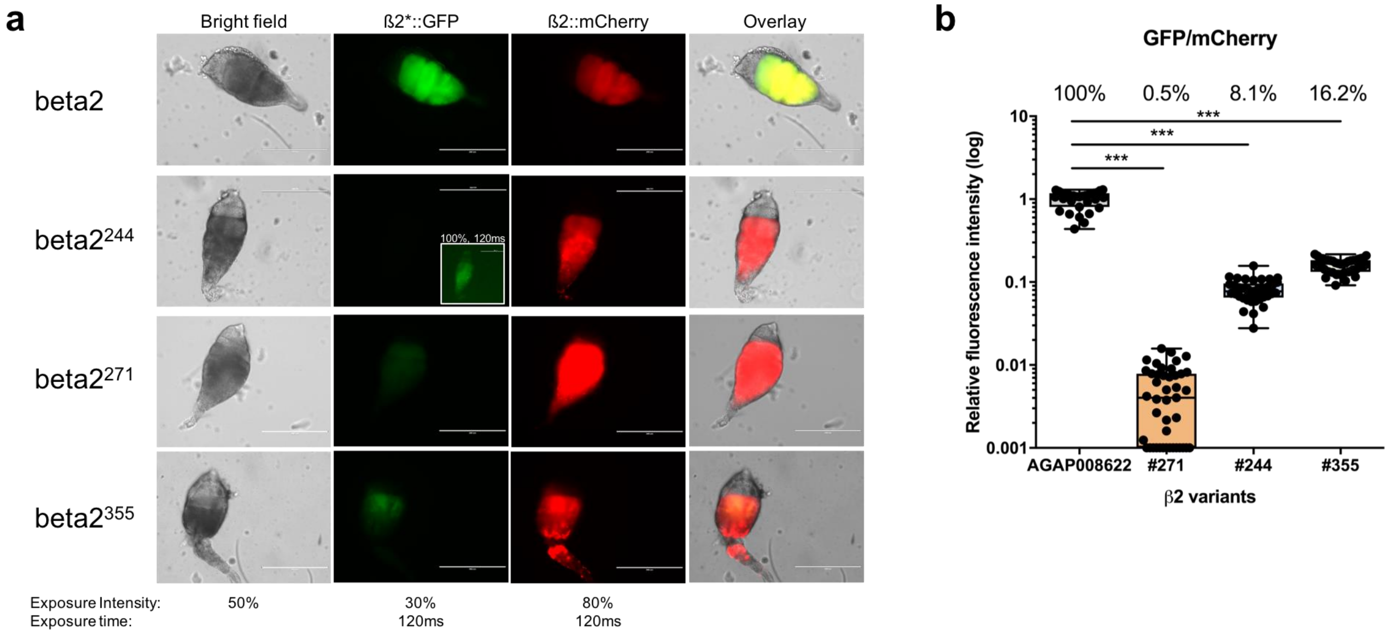

Supplementary Figure 6

GFP and mCherry signal quantification from mosquito testes transformed with modified *beta2* promoters.

a) Dissected testis from beta2^{wt}, beta2^{271}, beta2^{244} and beta2^{355} transgenic lines containing a single heterozygous insertion were microphotographed under the same exposure settings (as indicated), using mCherry as internal control. Line beta2^{271} showed GFP fluorescence intensity comparable to background; GFP expression was detectable at increased exposure (100% gain, 120ms, inset). Scale bar, 200 µm. b) Quantification of fluorescence intensity as GFP/mCherry ratio, normalized to the beta2^{wt} control (100%). Average relative intensity is indicated above the bars. *** indicates P value < 0.001 (ordinary One-way ANOVA). A minimum of 31 testes were analysed from individual expressing each promoter variant.

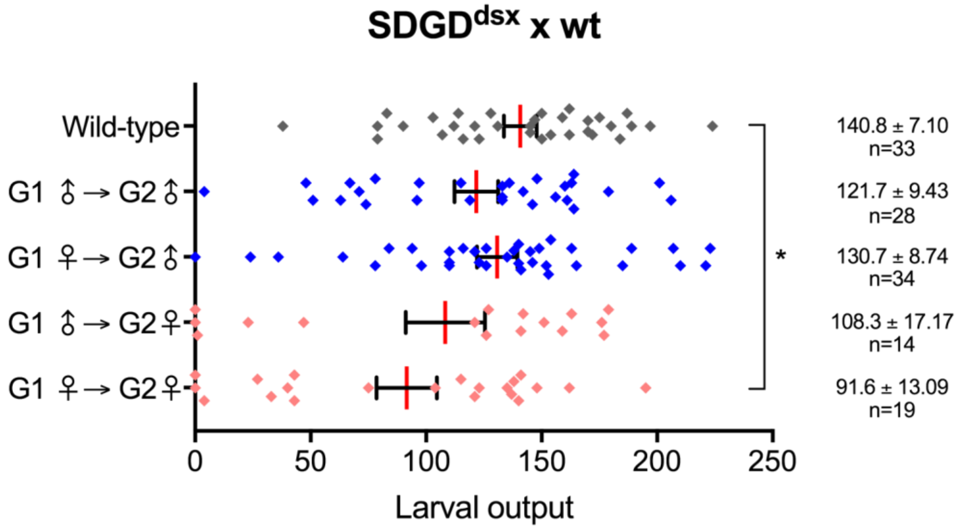

Supplementary Figure 7

Maternal or paternal contribution to the fecundity of the SDGD^{dsx} allele

Male and female SDGD^{dsx} heterozygotes that had inherited a maternal or paternal copy of the SDGD^{dsx} allele were crossed to wild type counterparts and assessed for fecundity. The total larval output is plotted for individual females (dots). Red bars indicate the average and the mean count (± s.e.m.) is shown. Females inheriting the transgene from the mother (G1♀ → G2♀) have significantly lower larval progeny (* P = 0.0256, Kruskal-Wallis test) compared to wild-type control.

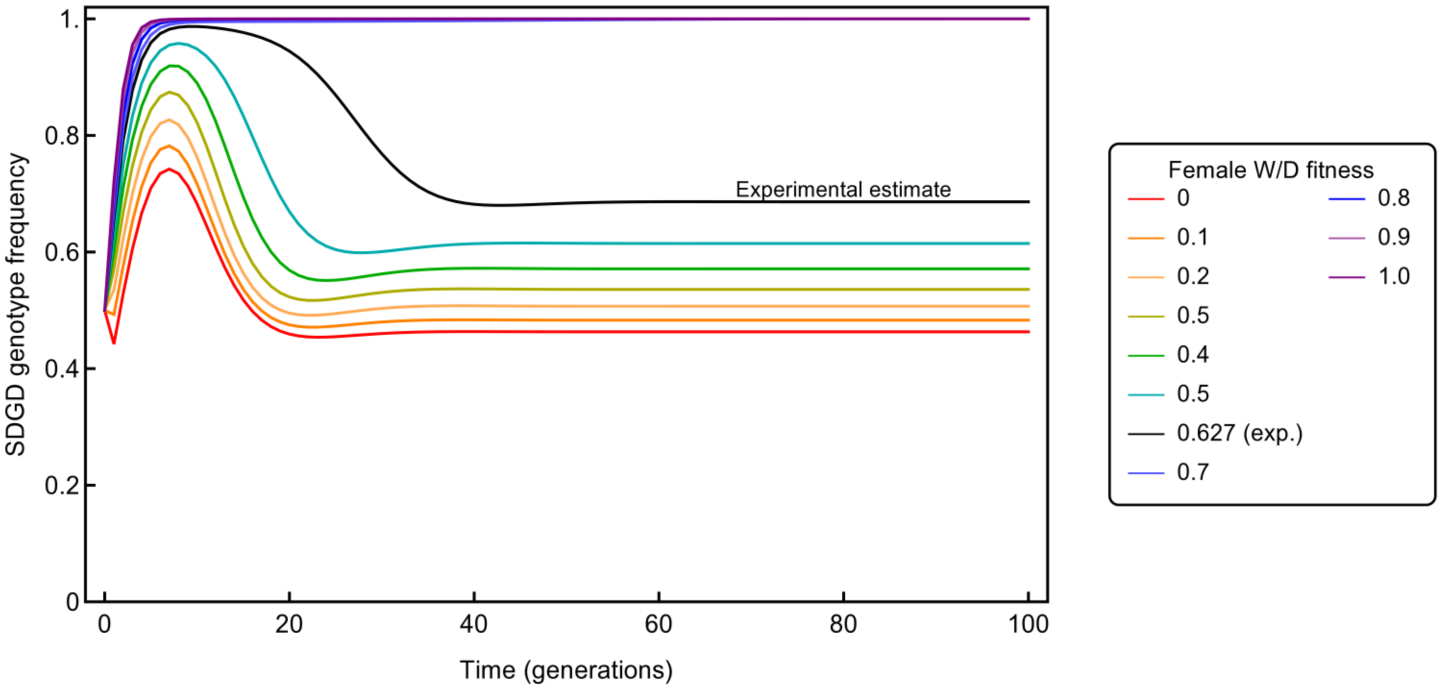

Supplementary Figure 8

Time dynamics of the frequency of SDGD^{dsx} as function of female fitness

Time dynamics of the frequency of SDGD^{dsx} transgenic individuals in the population as a function of W/D (where D represent the SDGD allele and W the wild-type allele) female fitness ($w_{\mathrm{WD,xx}}$), as predicted by the deterministic discrete-generation model at 25% initial allelic frequency. The graph shows the frequency of SDGD heterozygote males and females as a proportion of the male (or female) population, with other parameter estimates and baseline values given in Supp Table 2 (SDGD male fitness = 0.854; m (sex distortion) = 0.93). The predicted outcome at high W/D female fitness is elimination of the population, and at lower fitness, an intermediate equilibrium with W, R and D alleles. The result (black line) for the experimental estimate for female SDGD heterozygote fitness, $w_{\mathrm{WD,xx}} = 0.627$, is in a parameter region where even a small (positive) change leads to a prediction of population elimination instead of suppression.

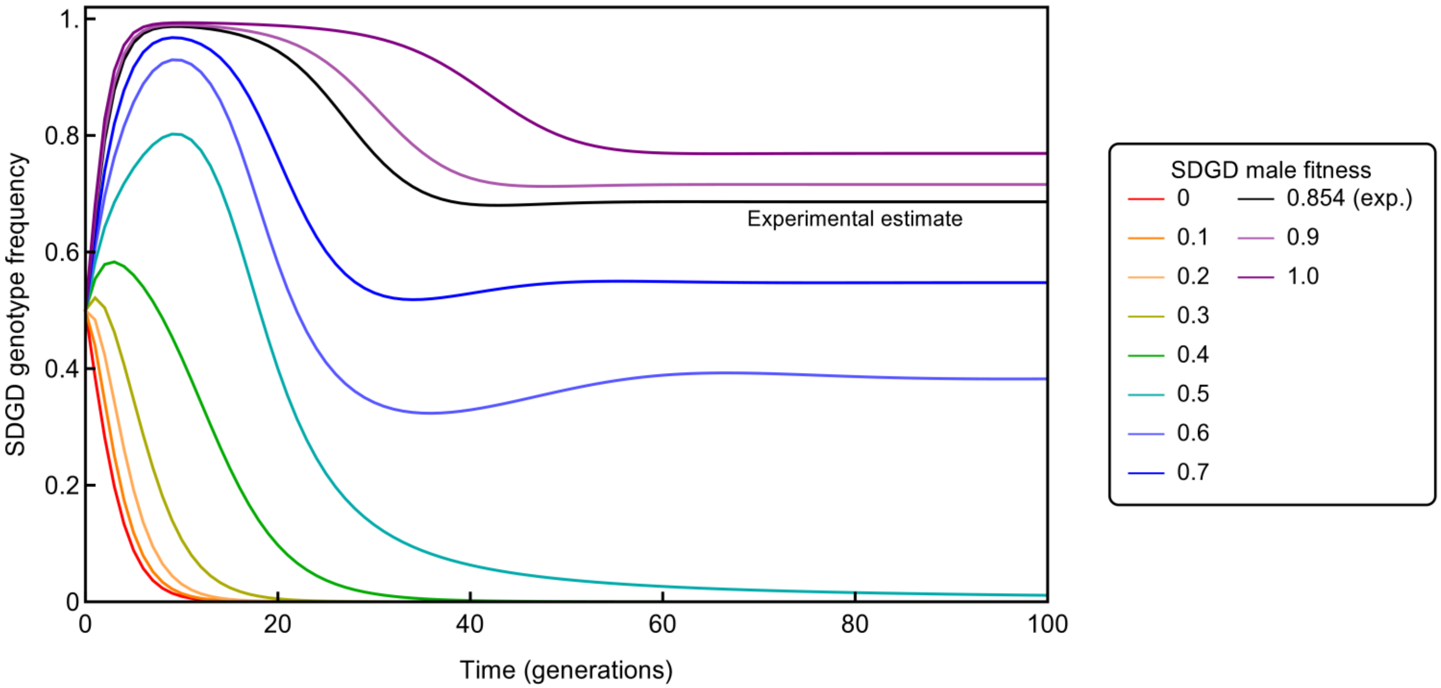

Supplementary Figure 9

Time dynamics of the frequency of $SDGD^{dsx}$ as function of male fitness

Time dynamics of the frequency of $SDGD^{dsx}$ transgenic individuals in the population as a function of the fitness of SDGD males (assume $w_{\mathrm{WD,xY}} = w_{\mathrm{DR,xY}} = w_{\mathrm{DD,xY}}$) as predicted by the deterministic discrete-generation model. Initial release is 50% of SDGD heterozygote males and females as a proportion of the male (or female) population, with other parameter estimates and baseline values given in Supp Table 2 (W/D female fitness $w_{\mathrm{WD,xx}}$ = 0.627; m = 0.93). For low SDGD male fitness ($<\approx 0.5$), the construct is eventually lost.

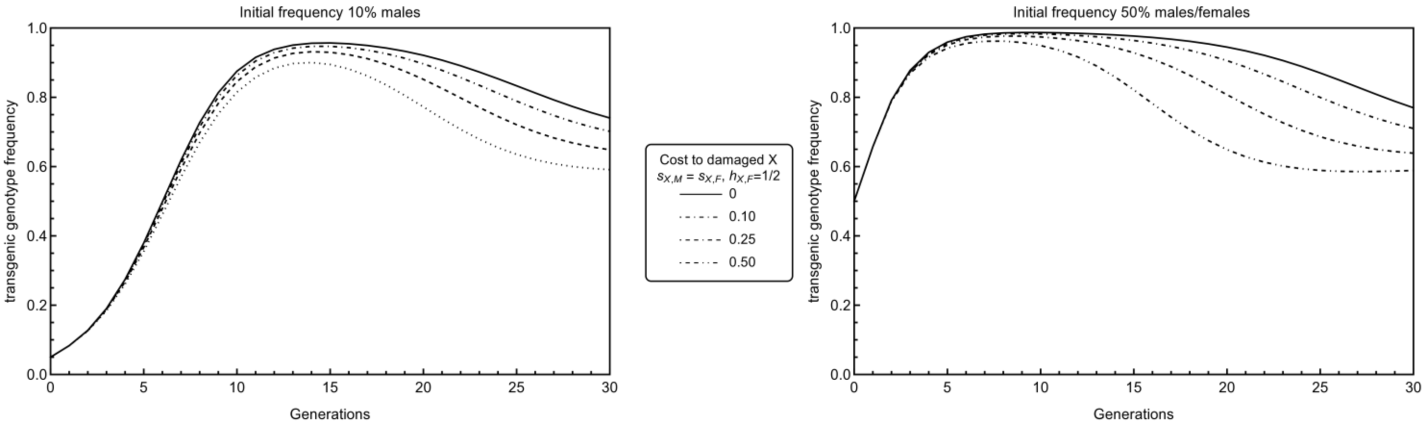

Supplementary Figure 10

Impact of fitness due to damaged X chromosome.

Modelling the impact of fitness reduction, due to the inheritance of damaged X chromosomes passed through X-shredding in a SDGD male, on the spread of the SDGD transgenics as predicted by the deterministic discrete-generation cage model. For simplicity, we assume that the additional cost to carrying one damaged X chromosome in males is the same as the cost in females that carry two copies of the damaged X ($s_{\mathrm{X,f}} = s_{\mathrm{X,m}}$), and females with one damaged X and one wildtype X chromosome have only half the fitness cost of females with two copies (dominance coefficient $h_{\mathrm{X,f}} = 1/2$). Estimates used for other parameters given in Supplementary Table 2.

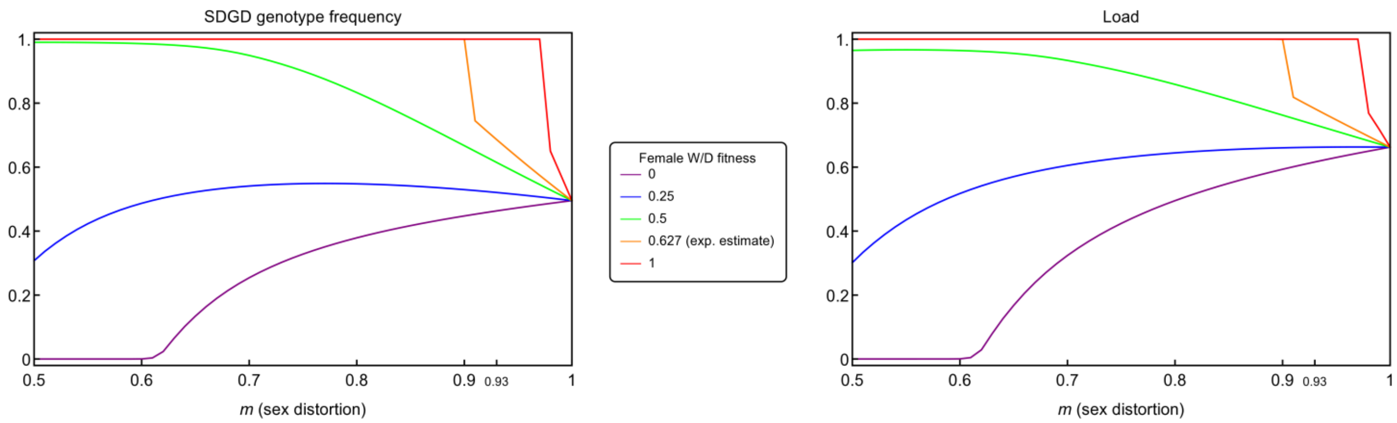

Supplementary Figure 11

Effect of female fitness on SDGD frequency and population load.

Effect of female W/D heterozygote fitness (D refers to the SDGD^{dsx} allele) on the SDGD genotype frequency (i.e., individuals with at least one copy of the SDGD) and load after 200 generations, as predicted by the deterministic discrete-generation model. Parameter estimates and baseline values given in Supp Table 2 (SDGD male fitness $= 0.854$). The possible outcomes (load $= 1$ and population elimination; intermediate equilibrium with W, D and R; or load $= 0$ and construct lost) depend upon the sex distortion *m* (0.5 [no sex bias] to 1 [only male progeny]) and the female W/D heterozygote fitness ($0 \leq w_{\mathrm{WD,xx}} \leq 1$). For low female fitness, adding an X-shredder (increasing sex distortion m) is predicted to beneficially increase the load on the population. For complete female heterozygous sterility (purple line), the drive construct disappears from the population if there is no sex distortion (*m* = 1/2), whereas sufficiently increasing the sex distortion allows the SDGD^{dsx} to spread and impose a load on the population. The presence of the X-shredder keeps the construct predominantly in males, therefore mitigating the heterozygous female sterility effect. By contrast if female fitness is sufficiently high (lines of fitness 0.5 or greater on plot), the load decreases at high sex distortion because due to male bias, SDGD^{dsx} males replace high-fitness W/D heterozygote females which decreases the ability of the construct to spread. For a complete male sex bias, *m* = 1, no W/D heterozygote females are created (and therefore no female/male SDGD^{dsx} homozygotes), since all X chromosomes are shredded and SDGD^{dsx} males have no female progeny; thus the load at *m* = 1 does not depend on the fitness of female W/D heterozygote individuals since none are present. Only SDGD^{dsx} heterozygous males can pass on the construct, with the SDGD^{dsx} allele present in reduced frequency in an intermediate equilibrium with wildtype and resistance alleles and a load less than one for these parameters. (In general, the amount of reduction in SDGD^{dsx} frequency and load at *m* = 1 will depend on parameters such as the homing rate, here less than 100%, the rate of resistant R mutation, and the relative fertility of the R and SDGD^{dsx} allele).

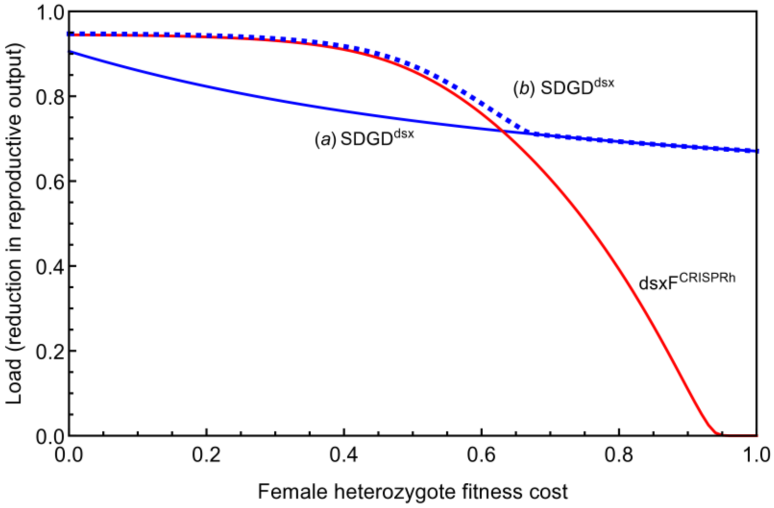

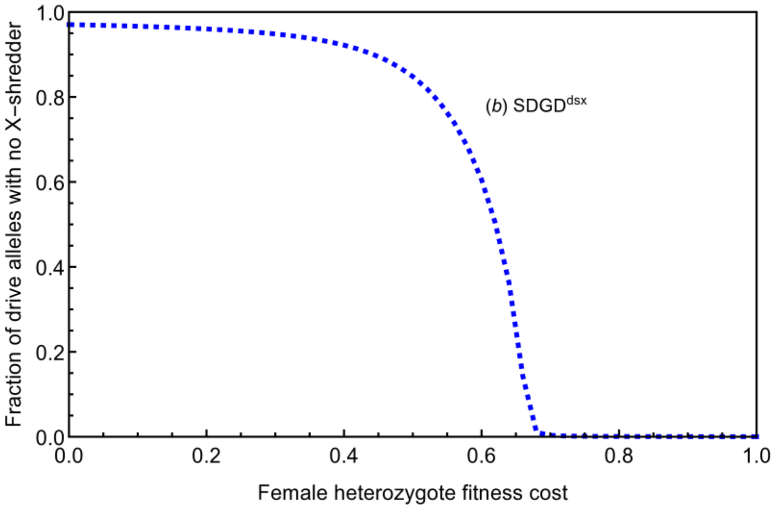

Supplementary Figure 12

Comparison of the predicted equilibrium for release of $SDGD^{dsx}$ or $dsxF^{CRISPRh}$ into a wild-type population

(Top panel) Comparison of the predicted equilibrium load (i.e., the reduction in reproductive output by the population after 400 generations) for release of $SDGD^{dsx}$ or $dsxF^{CRISPRh}$ into a wild-type population, varying the fitness cost to heterozygote W/D females. The discrete generation model predicts that the $SDGD^{dsx}$ construct (blue solid line) is more robust to reductions in female heterozygote fitness compared to $dsxF^{CRISPRh}$ (red line), still maintaining a substantial load even at 100% reduction in female heterozygote fitness (i.e. females heterozygous for the drive are completely non-viable). We also consider the possibility that the X-shredder component may be lost from the $SDGD^{dsx}$ construct during homing (bottom panel, blue dashed line), such that out of the drive alleles transmitted from female or male W/D individuals, 0.01% will not have a functioning X-shredder component. For low to mid-fitness costs, the predicted load is similar to that of the $dsxF^{CRISPRh}$ drive-only construct since the fraction of drive individuals without an intact X-shredder is high; for high fitness costs, the load merges with that of intact $SDGD^{dsx}$ (blue solid line) since almost all drive individuals have an intact X-shredder. We use representative parameters for both constructs for comparison (drive transmission $d_f = d_m = 0.95$ for both males and females; rate of resistance $u_f = u_m = 0.5$; X-shredding parameter for $SDGD^{dsx}$ is m=0.95; no reduction in fitness for heterozygote males).

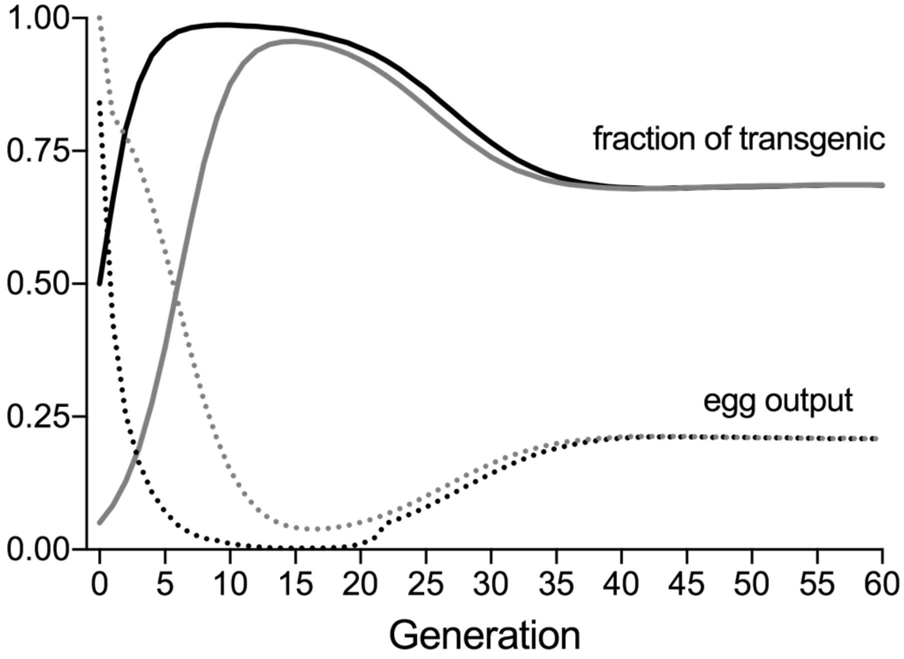

Supplementary Figure 13

Time dynamics of the frequency of SDGD^{dsx} transgenic individuals and relative egg output

Time dynamics of the frequency of SDGD^{dsx} transgenic individuals (solid lines) and relative eggs output (dotted lines) in the population as predicted by the deterministic discrete-generation model using experimental parameters given in Supp Table 2 and assuming two initial releases of 50% of SDGD heterozygote males and females (black line) or 10% SDGD heterozygous males only (grey line). Independently of the release scenarios, for these parameters, the frequency of transgenic individuals reaches an intermediate equilibrium while W, R and D alleles and the egg output is reduced (population suppressed).

Supplementary Tables

Supplementary Table 1 | Summary of values (± s.e.m.) obtained from crossed of the different SDGD developed in this study.

Target gene	Promoter Cas9	Promoter I-PpoI	Transgenic parent	N	Average eggs per female	Hatched larvae	Hatching rate	Transmission rate (RFP+)	Fraction of Males
AGAP007280	vasa2	beta2	Male x wt	23	16.9 ± 2.80	3.72 ± 1.02	0.23	na	na
			Female x wt	2	61.3 ± 42.12	2.45 ± 1.63	0.07	na	na
AGAP011377	vasa2	beta2	Male x wt	35	124.9 ± 8.18	97.5 ± 8.37	0.78	0.79 ± 0.17	0.92 ± 0.08
			Female x wt	20	5.3 ± 1.63	2.45 ± 1.03	0.47	0.69 ± 0.07	0.48 ± 0.13
AGAP005958	vasa2	beta2	Male x wt	33	97.5 ± 7.61	66.0 ± 7.99	0.68	0.94 ± 0.08	0.94 ± 0.08
			Female x wt	32	6.42 ± 1.41	0.1 ± 0.05	0.014	na	na
AGAP004050 (dsx)	zpg	$beta2^{244}$	Male x wt	71	130.2 ± 7.72	126.7 ± 6.39	0.84 ± 0.02	0.96 ± 0.01	0.93 ± 0.08
			Female x wt	44	115.1 ± 10.2	98.8 ± 10.4	0.67 ± 0.05	0.99 ± 0.01	0.48 ± 0.14

Supplementary Table 2 | Model parameters for Sex Distorter Gene Drive ($SDGD^{dsx}$)

Parameter	Estimate	Method of estimation
Mating probability ⱡ	0.85	Estimated from Hammond et al. 2017; Kyros et al. 2018
Egg production, wildtype female crossed with wildtype male ⱡ	150	From assays of mated females
Egg production, W/D female crossed with wildtype male ⱡ	115 (relative to 150 for WT = 0.77)	From assays of mated females
Egg production, wildtype female crossed with W/D maleⱡ	132 (relative to 150 for WT = 0.88)	From assays of mated females
Hatching probability, wildtype ⱡ	0.88	From assays of mated females
Hatching probability, W/D heterozygous female crossed with wildtype male ⱡ	0.72 (relative to 0.88 for WT = 0.81)	From assays of mated females
Hatching probability, W/D heterozygous male crossed with wildtype female ⱡ	0.86 (relative to 0.88 for WT = 0.97)	From assays of mated females
Fertility of W/D female (relative to WT, for deterministic model), ($w_{WD,xx}$)	0.627	From assays of mated females
Fertility of W/D male (relative to WT, for deterministic model)*	0.8536	From assays of mated females
Additional cost to females due to carrying two damaged X-chromosomes ($s_{X,f}$)	0	Baseline
Dominance coefficient for damaged X in females ($h_{X,f}$)	½	Baseline
Additional cost to males due to carrying damaged X-chromosome ($s_{X,m}$)	0	Baseline
Transmission of drive by W/D heterozygous females, (d_f)	1	Observed fraction transgenic from assays
Transmission of drive by W/D heterozygous males, (d_m)	0.96	Observed fraction transgenic from assays
Male sex bias of progeny of W/D heterozygous males ($m = m_1 = m_2$)	0.93	Observed from assays
Meiotic EJ parameter (fraction non-drive alleles that are resistant) ($u = u_f = u_m$)	0.469	Estimated from Hammond et al. 2016; Kyros et al. 2018
Probability of emergence from pupa (survival from larva) ⱡ	0.89	Estimated from Kyros et al. 2018
Initial population (zero generation) ⱡ	50% release: 150 WT males/females, 150 W/D heterozygous males/females; 10% release: 270 WT males, 30 W/D heterozygous males, 300 WT females	Following cage experiments

ⱡ For stochastic model. Note values are averages if there are parental effects (in fitnesses, drive).
*This fertility is assumed in the model for all males with one or two copies of the SDGD allele ($w_{WD,xY}, w_{DD,xY}, w_{DR,xY}$).

Supplementary Table 3 | Analysis of SDGD^{dsx} females with and without a copy of "modified" X chromosome.

Cross	Eggs[1]	% eggs hatching[2]	Transgenic rate[3]	Male progeny (% of sum ± SD)[4]	Male progeny (average of % ± SEM)[4]	P value
X^{mod}/X;SDGD^{dsx}/+ ♀<br>x<br>wild-type ♂	130.7 ± 15.2 (n=19)	69.8% n= 16 (2484)	100% (509)	47.1 ± 0.17 n=13 (1261)	0.41 ± 0.05, n=13 (1261)	n.s. (P= 0.242, t=1.203, df=21.09)
X/X;SDGD^{dsx}/+ ♀<br>x<br>wild-type ♂	103.2 ± 13.1 (n=25)	74.6% n=21 (2580)	100% (728)	50.5 ± 0.13 n=19 (1190)	0.48 ± 0.03, n=19 (1190)	

Average number of eggs laid by n number of females analysed (±SEM). (2) Average percentage of larvae hatching from the eggs, from n females analysed. (3) Average percentage of transgenic progeny (RFP positive) out of total larvae screened. (4) Average percentage of males in the progeny (±SD) established from n females. Significance (Welch's t-test, two-tailed, unpaired two-sample with unequal variance) was tested comparing logit transformed percentages observed from the progeny of n females of the two groups. The total number of eggs or individuals counted in each experiment is given in parentheses.

Supplementary Table 4 | The fitnesses of the 18 female genotypes and the proportion of each type of egg produced by them.

Female genotype	Fitness	Eggs produced					
		W, x	D, x	R, x	W, X	D, X	R, X
WW, xx	1	1	0	0	0	0	0
WD, xx	$w_{\mathrm{WD,xx}}$	$(1-d_f)\,(1-u_f)$	d_f	$(1-d_f)\,u_f$	0	0	0
WR, xx	$w_{\mathrm{WR,xx}}$	1/2	0	1/2	0	0	0
DD, xx	$w_{\mathrm{DD,xx}}$	0	1	0	0	0	0
DR, xx	$w_{\mathrm{DR,xx}}$	0	1/2	1/2	0	0	0
RR, xx	$w_{\mathrm{RR,xx}}$	0	0	1	0	0	0
WW, xX	$1-h_{X,f}s_{X,f}$	1/2	0	0	1/2	0	0
WD, xX	$w_{\mathrm{WD,xx}}(1-h_{X,f}s_{X,f})$	$1/2\,\left(1-d_f\right)(1-u_f)$	$d_f/2$	$1/2\,(1-d_f)\,u_f$	$1/2\left(1-d_f\right)(1-u_f)$	$d_f/2$	$1/2\,(1-d_f)\,u_f$
WR, xX	$w_{\mathrm{WR,xx}}(1-h_{X,f}s_{X,f})$	1/4	0	1/4	1/4	0	1/4
DD, xX	$w_{\mathrm{DD,xx}}(1-h_{X,f}s_{X,f})$	0	1/2	0	0	1/2	0
DR, xX	$w_{\mathrm{DR,xx}}(1-h_{X,f}s_{X,f})$	0	1/4	1/4	0	1/4	1/4
RR, xX	$w_{\mathrm{RR,xx}}(1-h_{X,f}s_{X,f})$	0	0	1/2	0	0	1/2
WW, XX	$1-s_{X,f}$	0	0	0	1	0	0
WD, XX	$w_{\mathrm{WD,xx}}(1-s_{X,f})$	0	0	0	$(1-d_f)\,(1-u_f)$	d_f	$(1-d_f)u_f$
WR, XX	$w_{\mathrm{WR,xx}}(1-s_{X,f})$	0	0	0	1/2	0	1/2
DD, XX	$w_{\mathrm{DD,xx}}(1-s_{X,f})$	0	0	0	0	1	0
DR, XX	$w_{\mathrm{DR,xx}}(1-s_{X,f})$	0	0	0	0	1/2	1/2
RR, XX	$w_{\mathrm{RR,xx}}(1-s_{X,f})$	0	0	0	0	0	1

Supplementary Table 5 | The fitnesses of the 12 male genotypes and the proportion of each type of sperm produced by them.

Male genotype	Fitness	Sperm produced							
		W, x	R, x	W, X	D, X	R, X	W, Y	D, Y	R, Y
WW, xY	1	1/2	0	0	0	0	1/2	0	0
WD, xY	$w_{\mathrm{WD,xY}}$	0	0	$(1-m_1)(1-d_m)(1-u_m)$	$(1-m_1)d_m$	$(1-m_1)(1-d_m)u_m$	$m_1(1-d_m)(1-u_m)$	$m_1 d_m$	$m_1(1-d_m)u_m$
WR, xY	$w_{\mathrm{WR,xY}}$	1/4	1/4	0	0	0	1/4	0	1/4
DD, xY	$w_{\mathrm{DD,xY}}$	0	0	0	$(1-m_2)$	0	0	m_2	0
DR, xY	$w_{\mathrm{DR,xY}}$	0	0	0	$(1-m_1)/2$	$(1-m_1)/2$	0	$m_1/2$	$m_1/2$
RR, xY	$w_{\mathrm{RR,xY}}$	0	1/2	0	0	0	0	0	1/2
WW, XY	$1-s_{X,m}$	0	0	1/2	0	0	1/2	0	0
WD, XY	$w_{\mathrm{WD,xY}}(1-s_{X,m})$	0	0	$(1-m_1)(1-d_m)(1-u_m)$	$(1-m_1)d_m$	$(1-m_1)(1-d_m)u_m$	$m_1(1-d_m)(1-u_m)$	$m_1 d_m$	$m_1(1-d_m)u_m$
WR, XY	$w_{\mathrm{WR,xY}}(1-s_{X,m})$	0	0	1/4	0	1/4	1/4	0	1/4
DD, XY	$w_{\mathrm{DD,xY}}(1-s_{X,m})$	0	0	0	$(1-m_2)$	0	0	m_2	0
DR, XY	$w_{\mathrm{DR,xY}}(1-s_{X,m})$	0	0	0	$(1-m_1)/2$	$(1-m_1)/2$	0	$m_1/2$	$m_1/2$
RR, XY	$w_{\mathrm{RR,xY}}(1-s_{X,m})$	0	0	0	0	1/2	0	0	1/2

Supplementary Note

Full sequence of following vectors (in GenBank format)

pB\(b2-mCherry)-attB-dsRFP-244beta2-GFP vector......6

pB\(b2-mCherry)-attB-dsRFP-271beta2-GFP......11

pB\(b2-mCherry)-attB-dsRFP-355beta2-GFP......16

pB\(b2-mCherry)-attB-dsRFP-WTbeta2-GFP_v2......20

pB\(b2-mCherry)-attB-dsRFP-244beta2-GFP vector

```
LOCUS       pB\(b2-mCherry)-attB-dsRFP-244beta2-GFP        7976 bp    DNA     circular UNA 05-APR-
2017
DEFINITION  Mars-244.
ACCESSION   urn.local...z-781tst0
VERSION     urn.local...z-781tst0
KEYWORDS    .
SOURCE
  ORGANISM  .
FEATURES             Location/Qualifiers
     rep_origin      21..327
                     /label="f1_origin"
     CDS             complement(471..614)
                     /modified_by="User"
                     /label="lacZ_a"
     promoter        600..616
                     /label="M13_forward20_primer"
     promoter        626..644
                     /label="T7_promoter"
     misc_feature    join(677..>711,<5296..5321)
                     /created_by="User"
                     /label="MCS"
     ligation        708..711
                     /label="Ligation"
     ligation        712..715
                     /label="Ligation"
     misc_feature    716..2513
                     /Source="referenceToDocument: urn:local:.:14s-6crwd4p"
                     /label="Digestion of pUC-mCherry - Fragment 3 extraction
                     (concatenated sequence 2)"
                     /note="Geneious type: Concatenated sequence"
     promoter        725..1223
                     /vntifkey=29
                     /label="b2\promoter"
     CDS             1230..1940
                     /vntifkey=4
                     /label="mCherry"
     misc_feature    1949..2435
                     /vntifkey=21
                     /label="b2\3'UTR"
     misc_recomb     2458..2513
                     /vntifkey=86
                     /label="attB"
     ligation        2514..2517
                     /label="Ligation"
     misc_feature    2517^2518
                     /Original_Bases="A"
                     /label="A"
                     /note="Geneious type: Editing History Deletion"
     misc_feature    2518..5289
                     /Source="referenceToDocument: urn:local:.:14s-6crwd4p"
                     /label="Digestion of pUC-RFP-244b2GFP - Fragment 1
                     extraction (concatenated sequence 3)"
                     /note="Geneious type: Concatenated sequence"
     primer_bind     2518..2538
                     /Sequence="ATGGTGCGCTCCTCCAAGAAC"
                     /Hairpin_Tm="40.3"
                     /Tm="63.0"
                     /Self_Dimer_Tm="19.3"
                     /%GC="57.1"
                     /created_by="primer3"
                     /label="aaa"
```

```
CDS             2518..3195
                /vntifkey=4
                /label="DsRed\(RFP)"
terminator      3206..3439
                /vntifkey=43
                /label="SV40"
promoter        3469..4067
                /vntifkey=21
                /modified_by="User"
                /label="b2promoter"
misc_feature    3484..3487
                /vntifkey=21
                /label="GATA"
misc_feature    3570..3619
                /created_by="User"
                /label="RE 0.41"
misc_feature    3578..3627
                /created_by="User"
                /label="RE 0.49"
misc_feature    3627..3676
                /created_by="User"
                /label="RE 0.36"
Synthetic       3721..3820
                /created_by="User"
                /modified_by="User"
                /label="Spacer"
misc_feature    3852..3855
                /vntifkey=21
                /label="GATA"
misc_feature    3962..3965
                /vntifkey=21
                /label="GATA"
misc_feature    4059..4062
                /vntifkey=21
                /label="GATA"
CDS             4071..>4787
                /vntifkey=4
                /modified_by="User"
                /label="eGFP"
terminator      4790..5285
                /vntifkey=21
                /modified_by="User"
                /label="b2terminator"
misc_feature    <5290..5295
                /Source="referenceToDocument: urn:local:.:1i-781tstq"
                /label="Annealed link-E2-F2 extraction (concatenated
                sequence 2)"
                /note="Geneious type: Concatenated sequence"
ligation        5290..5293
                /label="Ligation"
misc_feature    5294..711
                /label="Digestion of pBC-Linker-E2-F2 - Fragment 2
                (concatenated sequence 1)"
                /note="Geneious type: Concatenated sequence"
ligation        5296..5299
                /label="Ligation"
promoter        complement(5348..5367)
                /label="T3_promoter"
promoter        complement(5384..5402)
                /label="M13_reverse_primer"
misc_feature    complement(5401..5423)
                /label="M13_pUC_rev_primer"
promoter        complement(5437..5466)
                /label="lac_promoter"
primer_bind     complement(5700..5723)
                /Sequence="CCTGCGTTATCCCCTGATTCTGTG"
                /Hairpin_Tm="32.2"
                /Tm="63.3"
                /Self_Dimer_Tm="None"
                /%GC="54.2"
                /created_by="primer3"
                /label="Bluescript_r2"
primer_bind     5733..5754
                /Sequence="GTGAGCAAAAGGCCAGCAAAAG"
                /Hairpin_Tm="46.5"
                /Tm="61.4"
                /Self_Dimer_Tm="11.3"
                /%GC="50.0"
                /created_by="primer3"
                /Extension="TTAATTAA"
```

```
                     /modified_by="User"
                     /label="pBC_Pac"
     primer_bind     5754..5774
                     /Sequence="GGCCAGGAACCGTAAAAAGGC"
                     /Hairpin_Tm="None"
                     /Tm="62.1"
                     /Self_Dimer_Tm="0.9"
                     /%GC="57.1"
                     /created_by="primer3"
                     /Extension="TTAATTAA"
                     /modified_by="User"
                     /label="BluescriptPacI_f"
     rep_origin      complement(5775..6394)
                     /label="pBR322_origin"
     gene            6701..7360
                     /gene="CAT/CamR"
                     /label="CAT/CamR"
     CDS             6701..7360
                     /translation="MEKKITGYTTVDISQWHRKEHFEAFQSVAQCTYNQTVQLDITAF
                     LKTVKKNKHKFYPAFIHILARLMNAHPELRMAMKDGELVIWDSVHPCYTVFHEQTETF
                     SSLWSEYHDDFRQFLHIYSQDVACYGENLAYFPKGFIENMFFVSANPWVSFTSFDLNV
                     ANMDNFFAPVFTMGKYYTQGDKVLMPLAIQVHHAVCDGFHVGRMLNELQQYCDEWQGG
                     A*"
                     /label="ORF frame 1"
     gene            complement(7647..7847)
                     /gene="Ampicillin (860 - 660)"
                     /translation="MEKKITGYTTVDISQWHRKEHFEAFQSVAQCTYNQTVQLDITAF
                     LKTVKKNKHKFYPAFIHILARLMNAHPELRMAMKDGELVIWDSVHPCYTVFHEQTETF
                     SSLWSEYHDDFRQFLHIYSQDVACYGENLAYFPKGFIENMFFVSANPWVSFTSFDLNV
                     ANMDNFFAPVFTMGKYYTQGDKVLMPLAIQVHHAVCDGFHVGRMLNELQQYCDEWQGG
                     A*"
                     /label="Ampicillin (860 - 660)"
     promoter        complement(7890..7918)
                     /translation="MEKKITGYTTVDISQWHRKEHFEAFQSVAQCTYNQTVQLDITAF
                     LKTVKKNKHKFYPAFIHILARLMNAHPELRMAMKDGELVIWDSVHPCYTVFHEQTETF
                     SSLWSEYHDDFRQFLHIYSQDVACYGENLAYFPKGFIENMFFVSANPWVSFTSFDLNV
                     ANMDNFFAPVFTMGKYYTQGDKVLMPLAIQVHHAVCDGFHVGRMLNELQQYCDEWQGG
                     A*"
                     /label="AmpR_promoter"
ORIGIN
        1 ctgacgcgcc ctgtagcggc gcattaagcg cggcgggtgt ggtggttacg cgcagcgtga
       61 ccgctacact tgccagcgcc ctagcgcccg ctcctttcgc tttcttccct tcctttctcg
      121 ccacgttcgc cggctttccc cgtcaagctc taaatcgggg gctcccttta gggttccgat
      181 ttagtgcttt acggcacctc gaccccaaaa aacttgatta gggtgatggt tcacgtagtg
      241 ggccatcgcc ctgatagacg gtttttcgcc ctttgacgtt ggagtccacg ttctttaata
      301 gtggactctt gttccaaact ggaacaacac tcaaccctat ctcggtctat tcttttgatt
      361 tataagggat tttgccgatt tcggcctatt ggttaaaaaa tgagctgatt taacaaaaat
      421 ttaacgcgaa ttttaacaaa atattaacgc ttacaatttc cattcgccat tcaggctgcg
      481 caactgttgg gaagggcgat cggtgcgggc ctcttcgcta ttacgccagc tggcgaaagg
      541 gggatgtgct gcaaggcgat taagttgggt aacgccaggg ttttcccagt cacgacgttg
      601 taaaacgacg gccagtgagc gcgcgtaata cgactcacta tagggcgaat tggagctcca
      661 ccgcggtggc ggccgctcta gaactagtgg atcccccggg ctgcaggaat tatccccgac
      721 cggtctagcg ttcataattg atatagtttt gtaaatgaca tgacagtttt ttattttttt
      781 tttatccata attacgaatt gaacaactct acacacatat taattgcaag aacttatgct
      841 acataatatg gaggaaagtg gatgcatcat cccatccaag aagacatacg aatttatttg
      901 tggcatcgca atcggccgaa ccagcaaaag atgatcatag tagtcatatg agccgtacgt
      961 gccggatcat ttcgtgcaga accttcagag acgttggtcg acagattgat agaaactgtg
     1021 tagttagtcc attcacaaag ttgttccatt agggacaaaa gaaaaaaacg gcttaaacta
     1081 gaaatttgtg tacccagtag gaatcgcgta ttcggccgat agaggtcctt ccgtaagtat
     1141 tcccagcgct tagagagcaa cgctcgtgcg ttccaaaatc cgctaaatat caaacggctt
     1201 tcacagtttc gaaagatatc aagctcgaga tggtgagcaa gggcgaggag gataacatgg
     1261 ccatcatcaa ggagttcatg cgcttcaagg tgcacatgga gggctccgtg aacggccacg
     1321 agttcgagat cgagggcgag ggcgagggcc gcccctacga gggcacccag accgccaagc
     1381 tgaaggtgac caagggtggc cccctgccct tcgcctggga catcctgtcc cctcagttca
     1441 tgtacggctc caaggcctac gtgaagcacc ccgccgacat ccccgactac ttgaagctgt
     1501 ccttccccga gggcttcaag tgggagcgcg tgatgaactt cgaggacggc ggcgtggtga
     1561 ccgtgaccca ggactcctcc ctgcaggacg gcgagttcat ctacaaggtg aagctgcgcg
     1621 gcaccaactt cccctccgac ggccccgtaa tgcagaagaa gaccatgggc tgggaggcct
     1681 cctccgagcg gatgtacccc gaggacggcg ccctgaaggg cgagatcaag cagaggctga
     1741 agctgaagga cggcggccac tacgacgctg aggtcaagac cacctacaag gccaagaagc
     1801 ccgtgcagct gcccggcgcc tacaacgtca acatcaagtt ggacatcacc tcccacaacg
     1861 aggactacac catcgtggaa cagtacgaac gcgccgaggg ccgccactcc accggcggca
     1921 tggacgagct gtacaagtaa ttaattaact aaagctaaat tgaacaccct aaattatgtg
     1981 taaaatttct gctaagcagc ggtgttgggg tcaataaaaa tgttttttttc cactctattc
     2041 gcttcgtttt tgttgccatt tctcagtttt tgcttcgtac tcatgtgtaa ggattagtgc
     2101 agtgatggga agtagctccg aagttttctg gaatcgtttc cggatagtag gttcggtatt
     2161 agtttccgga atcggctccg gaattggttc cggaattgat tccgggatca gaattggctc
     2221 aaaattctca tggagattcc cagagtgatt tcgcttctga aacttcgtat ttaattcaag
     2281 aattgatccc cattctggag ctaattccaa ttctggagtc aattctgatt ctgttacccg
     2341 aacaaattgc gattcccagg tcaatgcccg ttccggggtn gattctgatt ccggagttgg
```

2401 aatcagctcc ggaattggaa tcggtcctta aatcgggcgc gccacatgtg gcccagctgc
2461 gggtgccagg gcgtgccctt gggctccccg ggcgcgtact ccacctcacc catcaacatg
2521 gtgcgctcct ccaagaacgt catcaaggag ttcatgcgct tcaaggtgcg catggagggc
2581 accgtgaacg gccacgagtt cgagatcgag ggcgagggcg agggccgccc ctacgagggc
2641 cacaacaccg tgaagctgaa ggtgaccaag ggcggccccc tgcccttcgc ctgggacatc
2701 ctgtcccccc agttccagta cggctccaag gtgtacgtga agcaccccgc cgacatcccc
2761 gactacaaga agctgtcctt ccccgagggc ttcaagtggg agcgcgtgat gaacttcgag
2821 gacggcggcg tggtgaccgt gacccaggac tcctccctgc aggacggctg cttcatctac
2881 aaggtgaagt tcatcggcgt gaacttcccc tccgacggcc ccgtaatgca gaagaagacc
2941 atgggctggg aggcctccac cgagcgcctg taccccgcg acggcgtgct gaagggcgag
3001 atccacaagg ccctgaagct gaaggacggc ggccactacc tggtggagtt caagtccatc
3061 tacatggcca agaagcccgt gcagctgccc ggctactact acgtggactc caagctggac
3121 atcacctccc acaacgagga ctacaccatc gtggagcagt acgagcgcac cgagggccgc
3181 caccacctgt tcctgtagcg gccgcgactc tagatcataa tcagccatac cacatttgta
3241 gaggttttac ttgctttaaa aaacctccca cacctccccc tgaacctgaa acataaaatg
3301 aatgcaattg ttgttgttaa cttgtttatt gcagcttata atggttacaa ataaagcaat
3361 agcatcacaa atttcacaaa taaagcattt ttttcactgc attctagttg tggtttgtcc
3421 aaactcatca atgtatctta aagcttatcg atacgcgtac ggcgcgccct agcgttcata
3481 attgatatag ttttgtaaat gacatgacag tttttatttt ttttttatc cataattacg
3541 aattgaacaa ctctacacac atattaattg caagaactta tgctacataa tatggaggaa
3601 agtggatgca tcatcccatc caagaagaca tacgaattta tttgtggcat cgcaatcggc
3661 cgaaccagca aaagatgatc atagtagtca tatgagccgt acgtgccgga tcatttcgtg
3721 gactcggacc cgagttggcc aacgacccac gggcggagtt agggcggatg gtgagaagtg
3781 cgcgtctcgt tcccgcagct cgccagcact ctcagactca cagaaccttc agagacgttg
3841 gtcgacagat tgatagaaac tgtgtagtta gtccattcac aaagttgttc cattagggac
3901 aaaagaaaaa aacggcttaa actagaaatt tgtgtaccca gtaggaatcg cgtattcggc
3961 cgatagaggt ccttccgtaa gtattcccag cgcttagaga gcaacgctcg tgcgttccaa
4021 aatccgctaa atatcaaacg gctttcacag tttcgaaaga tatcaagctt atggtgagca
4081 agggcgagga gctgttcacc ggggtggtgc ccatcctggt cgagctggac ggcgacgtaa
4141 acggccacaa gttcagcgtg tccggcgagg gcgagggcga tgccacctac ggcaagctga
4201 ccctgaagtt catctgcacc accggcaagc tgcccgtgcc ctggcccacc ctcgtgacca
4261 ccctgaccta cggcgtgcag tgcttcagcc gctaccccga ccacatgaag cagcacgact
4321 tcttcaagtc cgccatgccc gaaggctacg tccaggagcg caccatcttc ttcaaggacg
4381 acggcaacta caagacccgc gccgaggtga agttcgaggg cgacaccctg gtgaaccgca
4441 tcgagctgaa gggcatcgac ttcaaggagg acggcaacat cctggggcac aagctggagt
4501 acaactacaa cagccacaac gtctatatca tggccgacaa gcagaagaac ggcatcaagg
4561 tgaacttcaa gatccgccac aacatcgagg acggcagcgt gcagctcgcc gaccactacc
4621 agcagaacac ccccatcggc gacggccccg tgctgctgcc cgacaaccac tacctgagca
4681 cccagtccgc cctgagcaaa gaccccaacg agaagcgcga tcacatggtc ctgctggagt
4741 tcgtgaccgc cgccgggatc actctcggca tggacgagct gtacaagtaa gcttaactaa
4801 agctaaattg aacaccctaa attatgtgta aaatttctgc taagcagcgg tgttggggtc
4861 aataaaaatg tttttttcca ctctattcgc ttcgttttg ttgccatttc tcagtttttg
4921 cttcgtactc atgtgtaagg attagtgcag tgatgggaag tagctccgaa gttttctgga
4981 atcgtttccg gatagtaggt tcggtattag tttccggaat cggctccgga attggttccg
5041 gaattgattc cgggatcaga attggctcaa aattctcatg gagattccca gagtgatttc
5101 gcttctgaaa cttcgtattt aattcaagaa ttgatcccca ttctggagct aattccaatt
5161 ctggagtcaa ttctgattct gttacccgaa caaattgcga ttcccaggtc aatgcccatt
5221 ccggggacga ttctgattcc ggagttggaa tcagctccgg aattggaatc ggtccttaaa
5281 tcgggcgcgc ctaggagctt atcgataccg tcgacctcga gggggggccc ggtacccagc
5341 ttttgttccc tttagtgagg gttaattgcg cgcttggcgt aatcatggtc atagctgttt
5401 cctgtgtgaa attgttatcc gctcacaatt ccacacaaca tacgagccgg aagcataaag
5461 tgtaaagcct ggggtgccta atgagtgagc taactcacat taattgcgtt gcgctcactg
5521 cccgctttcc agtcgggaaa cctgtcgtgc cagctgcatt aatgaatcgg ccaacgcgcg
5581 gggagaggcg gtttgcgtat tgggcgctct tccgcttcct cgctcactga ctcgctgcgc
5641 tcggtcgttc ggctgcggcg agcggtatca gctcactcaa aggcggtaat acggttatcc
5701 acagaatcag gggataacgc aggaaagaac atgtgagcaa aaggccagca aaaggccagg
5761 aaccgtaaaa aggccgcgtt gctggcgttt ttccataggc tccgcccccc tgacgagcat
5821 cacaaaaatc gacgctcaag tcagaggtgg cgaaacccga caggactata aagataccag
5881 gcgtttcccc ctggaagctc cctcgtgcgc tctcctgttc cgaccctgcc gcttaccgga
5941 tacctgtccg cctttctccc ttcgggaagc gtggcgcttt ctcatagctc acgctgtagg
6001 tatctcagtt cggtgtaggt cgttcgctcc aagctgggct gtgtgcacga accccccgtt
6061 cagcccgacc gctgcgcctt atccggtaac tatcgtcttg agtccaaccc ggtaagacac
6121 gacttatcgc cactggcagc agccactggt aacaggatta gcagagcgag gtatgtaggc
6181 ggtgctacag agttcttgaa gtggtggcct aactacggct acactagaag gacagtattt
6241 ggtatctgcg ctctgctgaa gccagttacc ttcggaaaaa gagttggtag ctcttgatcc
6301 ggcaaacaaa ccaccgctgg tagcggtggt ttttttgttt gcaagcagca gattacgcgc
6361 agaaaaaaag gatctcaaga agatcctttg atcttttcta cggggtctga cgctcagtgg
6421 aacgaaaact cacgttaagg gattttggtc atgagattat caaaaaggat cttcacctag
6481 atccttttcg accgaataaa tacctgtgac ggaagatcac ttcgcagaat aaataaatcc
6541 tggtgtccct gttgataccg ggaagccctg ggccaacttt tggcgaaaat gagacgttga
6601 tcggcacgta agaggttcca actttcacca taatgaaata agatcactac cgggcgtatt
6661 ttttgagttg tcgagatttt caggagctaa ggaagctaaa atggagaaaa aaatcactgg
6721 atataccacc gttgatatat cccaatggca tcgtaaagaa cattttgagg catttcagtc
6781 agttgctcaa tgtacctata accagaccgt tcagctggat attacggcct ttttaaagac
6841 cgtaaagaaa aataagcaca agttttatcc ggcctttatt cacattcttg cccgcctgat
6901 gaatgctcat ccggaattac gtatggcaat gaaagacggt gagctggtga tatgggatag
6961 tgttcaccct tgttacaccg ttttccatga gcaaactgaa acgttttcat cgctctggag
7021 tgaataccac gacgatttcc ggcagtttct acacatatat tcgcaagatg tggcgtgtta
7081 cggtgaaaac ctggcctatt tccctaaagg gtttattgag aatatgtttt tcgtctcagc
7141 caatccctgg gtgagtttca ccagttttga tttaaacgtg gccaatatgg acaacttctt

```
     7201 cgcccccgtt ttcaccatgg gcaaatatta tacgcaaggc gacaaggtgc tgatgccgct
     7261 ggcgattcag gttcatcatg ccgtttgtga tggcttccat gtcggcagaa tgcttaatga
     7321 attacaacag tactgcgatg agtggcaggg cggggcgtaa tttttttaag gcagttattg
     7381 gtgcccttaa acgcctggtt gctacgcctg aataagtgat aataagcgga tgaatggcag
     7441 aaattcgaaa gcaaattcga cccggtcgtc ggttcagggc agggtcgtta aatagccgct
     7501 tatgtctatt gctggtttac cggtttattg actaccggaa gcagtgtgac cgtgtgcttc
     7561 tcaaatgcct gaggccagtt tgctcaggct ctccccgtgg aggtaataat tgacgatatg
     7621 atcctttttt tctgatcaaa agtgctcatc attggaaaac gttcttcggg gcgaaaactc
     7681 tcaaggatct taccgctgtt gagatccagt tcgatgtaac ccactcgtgc acccaactga
     7741 tcttcagcat cttttacttt caccagcgtt tctgggtgag caaaaacagg aaggcaaaat
     7801 gccgcaaaaa agggaataag ggcgacacgg aaatgttgaa tactcatact cttccttttt
     7861 caatattatt gaagcattta tcaagggtta ttgtctcatg agcggataca tatttgaatg
     7921 tatttagaaa aataaacaaa taggggttcc gcgcacattt ccccgaaaag tgccac
//
```

pB\(b2-mCherry)-attB-dsRFP-271beta2-GFP

```
LOCUS       pB\(b2-mCherry)-attB-dsRFP-271beta2-GFP        7976 bp    DNA     circular UNA 23-MAR-
2017
DEFINITION  Mars-271.
ACCESSION   urn.local...10-781tst0
VERSION     urn.local...10-781tst0
KEYWORDS    .
SOURCE
  ORGANISM  .
FEATURES             Location/Qualifiers
     rep_origin      21..327
                     /label="f1_origin"
     CDS             complement(471..614)
                     /modified_by="User"
                     /label="lacZ_a"
     promoter        600..616
                     /label="M13_forward20_primer"
     promoter        626..644
                     /label="T7_promoter"
     misc_feature    join(677..>711,<5296..5321)
                     /created_by="User"
                     /label="MCS"
     ligation        708..711
                     /label="Ligation"
     ligation        712..715
                     /label="Ligation"
     misc_feature    716..2513
                     /Source="referenceToDocument: urn:local:.:14s-6crwd4p"
                     /label="Digestion of pUC-mCherry - Fragment 3 extraction
                     (concatenated sequence 2)"
                     /note="Geneious type: Concatenated sequence"
     promoter        725..1223
                     /vntifkey=29
                     /label="b2\promoter"
     misc_feature    945..950
                     /Recognition_pattern="CA^TATG"
                     /annotation_group="CATATG"
                     /label="NdeI@1"
                     /note="Geneious type: restriction site"
     CDS             1230..1940
                     /vntifkey=4
                     /label="mCherry"
     misc_feature    1663..1668
                     /Recognition_pattern="C^CATGG"
                     /annotation_group="CCATGG"
                     /label="NcoI@1"
                     /note="Geneious type: restriction site"
     misc_feature    1949..2435
                     /vntifkey=21
                     /label="b2\3'UTR"
     misc_recomb     2458..2513
                     /vntifkey=86
                     /label="attB"
     ligation        2514..2517
                     /label="Ligation"
     misc_feature    2517^2518
                     /Original_Bases="A"
                     /label="A"
                     /note="Geneious type: Editing History Deletion"
     misc_feature    2518..5289
                     /Source="referenceToDocument: urn:local:.:14s-6crwd4p"
                     /label="Digestion of pUC-RFP-271b2GFP - Fragment 1
                     extraction (concatenated sequence 3)"
                     /note="Geneious type: Concatenated sequence"
     primer_bind     2518..2538
                     /Sequence="ATGGTGCGCTCCTCCAAGAAC"
                     /Hairpin_Tm="40.3"
                     /Tm="63.0"
                     /Self_Dimer_Tm="19.3"
                     /%GC="57.1"
                     /created_by="primer3"
                     /label="aaa"
     CDS             2518..3195
                     /vntifkey=4
                     /label="DsRed\(RFP)"
     misc_feature    2939..2944
                     /Recognition_pattern="C^CATGG"
                     /annotation_group="CCATGG"
                     /label="NcoI@1"
```

```
                     /note="Geneious type: restriction site"
terminator           3206..3439
                     /vntifkey=43
                     /label="SV40"
promoter             3469..4067
                     /vntifkey=21
                     /modified_by="User"
                     /label="b2promoter"
misc_feature         3484..3487
                     /vntifkey=21
                     /label="GATA"
misc_feature         3570..3619
                     /created_by="User"
                     /label="RE 0.41"
misc_feature         3578..3627
                     /created_by="User"
                     /label="RE 0.49"
misc_feature         3627..3676
                     /created_by="User"
                     /label="RE 0.36"
misc_feature         3689..3694
                     /Recognition_pattern="CA^TATG"
                     /annotation_group="CATATG"
                     /label="NdeI@1"
                     /note="Geneious type: restriction site"
Synthetic            3694..3793
                     /created_by="User"
                     /modified_by="User"
                     /label="spacer"
misc_feature         3852..3855
                     /vntifkey=21
                     /label="GATA"
misc_feature         3962..3965
                     /vntifkey=21
                     /label="GATA"
misc_feature         4059..4062
                     /vntifkey=21
                     /label="GATA"
CDS                  4071..>4787
                     /vntifkey=4
                     /modified_by="User"
                     /label="eGFP"
terminator           4790..5285
                     /vntifkey=21
                     /modified_by="User"
                     /label="b2terminator"
misc_feature         <5290..5295
                     /Source="referenceToDocument: urn:local:.:1l-781tstv"
                     /label="Annealed link-E2-F2 extraction (concatenated
                     sequence 2)"
                     /note="Geneious type: Concatenated sequence"
ligation             5290..5293
                     /label="Ligation"
misc_feature         5294..711
                     /label="Digestion of pBC-Linker-E2-F2 - Fragment 2
                     (concatenated sequence 1)"
                     /note="Geneious type: Concatenated sequence"
ligation             5296..5299
                     /label="Ligation"
promoter             complement(5348..5367)
                     /label="T3_promoter"
promoter             complement(5384..5402)
                     /label="M13_reverse_primer"
misc_feature         complement(5401..5423)
                     /label="M13_pUC_rev_primer"
promoter             complement(5437..5466)
                     /label="lac_promoter"
primer_bind          complement(5700..5723)
                     /Sequence="CCTGCGTTATCCCCTGATTCTGTG"
                     /Hairpin_Tm="32.2"
                     /Tm="63.3"
                     /Self_Dimer_Tm="None"
                     /%GC="54.2"
                     /created_by="primer3"
                     /label="Bluescript_r2"
primer_bind          5733..5754
                     /Sequence="GTGAGCAAAAGGCCAGCAAAAG"
                     /Hairpin_Tm="46.5"
                     /Tm="61.4"
                     /Self_Dimer_Tm="11.3"
```

```
                     /%GC="50.0"
                     /created_by="primer3"
                     /Extension="TTAATTAA"
                     /modified_by="User"
                     /label="pBC_Pac"
     primer_bind     5754..5774
                     /Sequence="GGCCAGGAACCGTAAAAAGGC"
                     /Hairpin_Tm="None"
                     /Tm="62.1"
                     /Self_Dimer_Tm="0.9"
                     /%GC="57.1"
                     /created_by="primer3"
                     /Extension="TTAATTAA"
                     /modified_by="User"
                     /label="BluescriptPacI_f"
     rep_origin      complement(5775..6394)
                     /label="pBR322_origin"
     gene            6701..7360
                     /gene="CAT/CamR"
                     /label="CAT/CamR"
     CDS             6701..7360
                     /translation="MEKKITGYTTVDISQWHRKEHFEAFQSVAQCTYNQTVQLDITAF
                     LKTVKKNKHKFYPAFIHILARLMNAHPELRMAMKDGELVIWDSVHPCYTVFHEQTETF
                     SSLWSEYHDDFRQFLHIYSQDVACYGENLAYFPKGFIENMFFVSANPWVSFTSFDLNV
                     ANMDNFFAPVFTMGKYYTQGDKVLMPLAIQVHHAVCDGFHVGRMLNELQQYCDEWQGG
                     A*"
                     /label="ORF frame 1"
     misc_feature    7215..7220
                     /Recognition_pattern="C^CATGG"
                     /annotation_group="CCATGG"
                     /label="NcoI@1"
                     /note="Geneious type: restriction site"
     gene            complement(7647..7847)
                     /gene="Ampicillin (860 - 660)"
                     /translation="MEKKITGYTTVDISQWHRKEHFEAFQSVAQCTYNQTVQLDITAF
                     LKTVKKNKHKFYPAFIHILARLMNAHPELRMAMKDGELVIWDSVHPCYTVFHEQTETF
                     SSLWSEYHDDFRQFLHIYSQDVACYGENLAYFPKGFIENMFFVSANPWVSFTSFDLNV
                     ANMDNFFAPVFTMGKYYTQGDKVLMPLAIQVHHAVCDGFHVGRMLNELQQYCDEWQGG
                     A*"
                     /label="Ampicillin (860 - 660)"
     promoter        complement(7890..7918)
                     /translation="MEKKITGYTTVDISQWHRKEHFEAFQSVAQCTYNQTVQLDITAF
                     LKTVKKNKHKFYPAFIHILARLMNAHPELRMAMKDGELVIWDSVHPCYTVFHEQTETF
                     SSLWSEYHDDFRQFLHIYSQDVACYGENLAYFPKGFIENMFFVSANPWVSFTSFDLNV
                     ANMDNFFAPVFTMGKYYTQGDKVLMPLAIQVHHAVCDGFHVGRMLNELQQYCDEWQGG
                     A*"
                     /label="AmpR_promoter"
ORIGIN
        1 ctgacgcgcc ctgtagcggc gcattaagcg cggcgggtgt ggtggttacg cgcagcgtga
       61 ccgctacact tgccagcgcc ctagcgcccg ctcctttcgc tttcttccct tcctttctcg
      121 ccacgttcgc cggctttccc cgtcaagctc taaatcgggg gctcccttta gggttccgat
      181 ttagtgcttt acggcacctc gaccccaaaa aacttgatta gggtgatggt tcacgtagtg
      241 ggccatcgcc ctgatagacg gtttttcgcc ctttgacgtt ggagtccacg ttctttaata
      301 gtggactctt gttccaaact ggaacaacac tcaaccctat ctcggtctat tcttttgatt
      361 tataagggat tttgccgatt tcggcctatt ggttaaaaaa tgagctgatt taacaaaaat
      421 ttaacgcgaa ttttaacaaa atattaacgc ttacaatttc cattcgccat tcaggctgcg
      481 caactgttgg gaagggcgat cggtgcgggc ctcttcgcta ttacgccagc tggcgaaagg
      541 gggatgtgct gcaaggcgat taagttgggt aacgccaggg ttttcccagt cacgacgttg
      601 taaaacgacg gccagtgagc gcgcgtaata cgactcacta tagggcgaat tggagctcca
      661 ccgcggtggc ggccgctcta gaactagtgg atcccccggg ctgcaggaat tatccccgac
      721 cggtctagcg ttcataattg atatagtttt gtaaatgaca tgacagtttt ttattttttt
      781 tttatccata attacgaatt gaacaactct acacacatat taattgcaag aacttatgct
      841 acataatatg gaggaaagtg gatgcatcat cccatccaag aagacatacg aatttatttg
      901 tggcatcgca atcggccgaa ccagcaaaag atgatcatag tagtcatatg agccgtacgt
      961 gccggatcat ttcgtgcaga accttcagag acgttggtcg acagattgat agaaactgtg
     1021 tagttagtcc attcacaaag ttgttccatt agggacaaaa gaaaaaaacg gcttaaacta
     1081 gaaatttgtg tacccagtag gaatcgcgta ttcggccgat agaggtcctt ccgtaagtat
     1141 tcccagcgct tagagagcaa cgctcgtgcg ttccaaaatc cgctaaatat caaacggctt
     1201 tcacagtttc gaaagatatc aagctcgaga tggtgagcaa gggcgaggag gataacatgg
     1261 ccatcatcaa ggagttcatg cgcttcaagg tgcacatgga gggctccgtg aacggccacg
     1321 agttcgagat cgagggcgag ggcgagggcc gcccctacga gggcacccag accgccaagc
     1381 tgaaggtgac caagggtggc cccctgccct tcgcctggga catcctgtcc cctcagttca
     1441 tgtacggctc caaggcctac gtgaagcacc ccgccgacat ccccgactac ttgaagctgt
     1501 ccttccccga gggcttcaag tgggagcgcg tgatgaactt cgaggacggc ggcgtggtga
     1561 ccgtgaccca ggactcctcc ctgcaggacg gcgagttcat ctacaaggtg aagctgcgcg
     1621 gcaccaactt cccctccgac ggccccgtaa tgcagaagaa gaccatgggc tgggaggcct
     1681 cctccgagcg gatgtacccc gaggacggcg ccctgaaggg cgagatcaag cagaggctga
     1741 agctgaagga cggcggccac tacgacgctg aggtcaagac cacctacaag gccaagaagc
     1801 ccgtgcagct gcccggcgcc tacaacgtca acatcaagtt ggacatcacc tcccacaacg
     1861 aggactacac catcgtggaa cagtacgaac gcgccgaggg ccgccactcc accggcggca
```

1921 tggacgagct gtacaagtaa ttaattaact aaagctaaat tgaacaccct aaattatgtg
1981 taaaatttct gctaagcagc ggtgttgggg tcaataaaaa tgtttttttc cactctattc
2041 gcttcgtttt tgttgccatt tctcagtttt tgcttcgtac tcatgtgtaa ggattagtgc
2101 agtgatggga agtagctccg aagttttctg gaatcgtttc cggatagtag gttcggtatt
2161 agtttccgga atcggctccg gaattggttc cggaattgat tccgggatca gaattggctc
2221 aaaattctca tggagattcc cagagtgatt tcgcttctga aacttcgtat ttaattcaag
2281 aattgatccc cattctggag ctaattccaa ttctggagtc aattctgatt ctgttacccg
2341 aacaaattgc gattcccagg tcaatgcccg ttccggggtn gattctgatt ccggagttgg
2401 aatcagctcc ggaattggaa tcggtcctta aatcgggcgc gccacatgtg gcccagctgc
2461 gggtgccagg gcgtgccctt gggctccccg ggcgcgtact ccacctcacc catcaacatg
2521 gtgcgctcct ccaagaacgt catcaaggag ttcatgcgct tcaaggtgcg catggagggc
2581 accgtgaacg gccacgagtt cgagatcgag ggcgagggcg agggccgccc ctacgagggc
2641 cacaacaccg tgaagctgaa ggtgaccaag ggcggccccc tgcccttcgc ctgggacatc
2701 ctgtcccccc agttccagta cggctccaag gtgtacgtga agcaccccgc cgacatcccc
2761 gactacaaga agctgtcctt ccccgagggc ttcaagtggg agcgcgtgat gaacttcgag
2821 gacggcggcg tggtgaccgt gacccaggac tcctccctgc aggacggctg cttcatctac
2881 aaggtgaagt tcatcggcgt gaacttcccc tccgacggcc ccgtaatgca gaagaagacc
2941 atgggctggg aggcctccac cgagcgcctg tacccccgcg acggcgtgct gaagggcgag
3001 atccacaagg ccctgaagct gaaggacggc ggccactacc tggtggagtt caagtccatc
3061 tacatggcca agaagcccgt gcagctgccc ggctactact acgtggactc caagctggac
3121 atcacctccc acaacgagga ctacaccatc gtggagcagt acgagcgcac cgagggccgc
3181 caccacctgt tcctgtagcg gccgcgactc tagatcataa tcagccatac cacatttgta
3241 gaggttttac ttgctttaaa aaacctccca cacctccccc tgaacctgaa acataaaatg
3301 aatgcaattg ttgttgttaa cttgtttatt gcagcttata atggttacaa ataaagcaat
3361 agcatcacaa atttcacaaa taaagcattt ttttcactgc attctagttg tggtttgtcc
3421 aaactcatca atgtatctta aagcttatcg atacgcgtac ggcgcgccct agcgttcata
3481 attgatatag ttttgtaaat gacatgacag tttttttattt tttttttatc cataattacg
3541 aattgaacaa ctctacacac atattaattg caagaactta tgctacataa tatggaggaa
3601 agtggatgca tcatcccatc caagaagaca tacgaattta tttgtggcat cgcaatcggc
3661 cgaaccagca aaagatgatc atagtagtca tatgactcgg acccgagttg gccaacgacc
3721 cacgggcgga gttagggcgg atggtgagaa gtgcgcgtct cgttcccgca gctcgccagc
3781 actctcagac tcagagccgt acgtgccgga tcatttcgtg cagaaccttc agagacgttg
3841 gtcgacagat tgatagaaac tgtgtagtta gtccattcac aaagttgttc cattagggac
3901 aaaagaaaaa aacggcttaa actagaaatt tgtgtaccca gtaggaatcg cgtattcggc
3961 cgatagaggt ccttccgtaa gtattcccag cgcttagaga gcaacgctcg tgcgttccaa
4021 aatccgctaa atatcaaacg gctttcacag tttcgaaaga tatcaagctt atggtgagca
4081 agggcgagga gctgttcacc ggggtggtgc ccatcctggt cgagctggac ggcgacgtaa
4141 acggccacaa gttcagcgtg tccggcgagg gcgagggcga tgccacctac ggcaagctga
4201 ccctgaagtt catctgcacc accggcaagc tgcccgtgcc ctggcccacc ctcgtgacca
4261 ccctgaccta cggcgtgcag tgcttcagcc gctaccccga ccacatgaag cagcacgact
4321 tcttcaagtc cgccatgccc gaaggctacg tccaggagcg caccatcttc ttcaaggacg
4381 acggcaacta caagacccgc gccgaggtga agttcgaggg cgacaccctg gtgaaccgca
4441 tcgagctgaa gggcatcgac ttcaaggagg acggcaacat cctggggcac aagctggagt
4501 acaactacaa cagccacaac gtctatatca tggccgacaa gcagaagaac ggcatcaagg
4561 tgaacttcaa gatccgccac aacatcgagg acggcagcgt gcagctcgcc gaccactacc
4621 agcagaacac ccccatcggc gacggccccg tgctgctgcc cgacaaccac tacctgagca
4681 cccagtccgc cctgagcaaa gaccccaacg agaagcgcga tcacatggtc ctgctggagt
4741 tcgtgaccgc cgccgggatc actctcggca tggacgagct gtacaagtaa gcttaactaa
4801 agctaaattg aacaccctaa attatgtgta aaatttctgc taagcagcgg tgttggggtc
4861 aataaaaatg tttttttcca ctctattcgc ttcgtttttg ttgccatttc tcagtttttg
4921 cttcgtactc atgtgtaagg attagtgcag tgatgggaag tagctccgaa gttttctgga
4981 atcgtttccg gatagtaggt tcggtattag tttccggaat cggctccgga attggttccg
5041 gaattgattc cgggatcaga attggctcaa aattctcatg gagattccca gagtgatttc
5101 gcttctgaaa cttcgtattt aattcaagaa ttgatcccca ttctggagct aattccaatt
5161 ctggagtcaa ttctgattct gttacccgaa caaattgcga ttcccaggtc aatgcccatt
5221 ccggggacga ttctgattcc ggagttggaa tcagctccgg aattggaatc ggtccttaaa
5281 tcgggcgcgc ctaggagctt atcgataccg tcgacctcga gggggggccc ggtacccagc
5341 ttttgttccc tttagtgagg gttaattgcg cgcttggcgt aatcatggtc atagctgttt
5401 cctgtgtgaa attgttatcc gctcacaatt ccacacaaca tacgagccgg aagcataaag
5461 tgtaaagcct ggggtgccta atgagtgagc taactcacat taattgcgtt gcgctcactg
5521 cccgctttcc agtcgggaaa cctgtcgtgc cagctgcatt aatgaatcgg ccaacgcgcg
5581 gggagaggcg gtttgcgtat tgggcgctct tccgcttcct cgctcactga ctcgctgcgc
5641 tcggtcgttc ggctgcggcg agcggtatca gctcactcaa aggcggtaat acggttatcc
5701 acagaatcag gggataacgc aggaaagaac atgtgagcaa aaggccagca aaaggccagg
5761 aaccgtaaaa aggccgcgtt gctggcgttt ttccataggc tccgcccccc tgacgagcat
5821 cacaaaaatc gacgctcaag tcagaggtgg cgaaacccga caggactata aagataccag
5881 gcgtttcccc ctggaagctc cctcgtgcgc tctcctgttc cgaccctgcc gcttaccgga
5941 tacctgtccg cctttctccc ttcgggaagc gtggcgcttt ctcatagctc acgctgtagg
6001 tatctcagtt cggtgtaggt cgttcgctcc aagctgggct gtgtgcacga accccccgtt
6061 cagcccgacc gctgcgcctt atccggtaac tatcgtcttg agtccaaccc ggtaagacac
6121 gacttatcgc cactggcagc agccactggt aacaggatta gcagagcgag gtatgtaggc
6181 ggtgctacag agttcttgaa gtggtggcct aactacggct acactagaag gacagtattt
6241 ggtatctgcg ctctgctgaa gccagttacc ttcggaaaaa gagttggtag ctcttgatcc
6301 ggcaaacaaa ccaccgctgg tagcggtggt ttttttgttt gcaagcagca gattacgcgc
6361 agaaaaaaag gatctcaaga agatcctttg atcttttcta cggggtctga cgctcagtgg
6421 aacgaaaact cacgttaagg gattttggtc atgagattat caaaaaggat cttcacctag
6481 atccttttcg accgaataaa tacctgtgac ggaagatcac ttcgcagaat aaataaatcc
6541 tggtgtccct gttgataccg ggaagccctg ggccaacttt tggcgaaaat gagacgttga
6601 tcggcacgta agaggttcca actttcacca taatgaaata agatcactac cgggcgtatt
6661 ttttgagttg tcgagatttt caggagctaa ggaagctaaa atggagaaaa aaatcactgg

6721 atataccacc gttgatatat cccaatggca tcgtaaagaa cattttgagg catttcagtc
6781 agttgctcaa tgtacctata accagaccgt tcagctggat attacggcct ttttaaagac
6841 cgtaaagaaa aataagcaca agttttatcc ggcctttatt cacattcttg cccgcctgat
6901 gaatgctcat ccggaattac gtatggcaat gaaagacggt gagctggtga tatgggatag
6961 tgttcaccct tgttacaccg ttttccatga gcaaactgaa acgttttcat cgctctggag
7021 tgaataccac gacgatttcc ggcagtttct acacatatat tcgcaagatg tggcgtgtta
7081 cggtgaaaac ctggcctatt tccctaaagg gtttattgag aatatgtttt tcgtctcagc
7141 caatccctgg gtgagtttca ccagttttga tttaaacgtg gccaatatgg acaacttctt
7201 cgcccccgtt ttcaccatgg gcaaatatta tacgcaaggc gacaaggtgc tgatgccgct
7261 ggcgattcag gttcatcatg ccgtttgtga tggcttccat gtcggcagaa tgcttaatga
7321 attacaacag tactgcgatg agtggcaggg cggggcgtaa tttttttaag gcagttattg
7381 gtgcccttaa acgcctggtt gctacgcctg aataagtgat aataagcgga tgaatggcag
7441 aaattcgaaa gcaaattcga cccggtcgtc ggttcagggc agggtcgtta aatagccgct
7501 tatgtctatt gctggtttac cggtttattg actaccggaa gcagtgtgac cgtgtgcttc
7561 tcaaatgcct gaggccagtt tgctcaggct ctccccgtgg aggtaataat tgacgatatg
7621 atcctttttt tctgatcaaa agtgctcatc attggaaaac gttcttcggg gcgaaaactc
7681 tcaaggatct taccgctgtt gagatccagt tcgatgtaac ccactcgtgc acccaactga
7741 tcttcagcat cttttacttt caccagcgtt tctgggtgag caaaaacagg aaggcaaaat
7801 gccgcaaaaa agggaataag ggcgacacgg aaatgttgaa tactcatact cttccttttt
7861 caatattatt gaagcattta tcaagggtta ttgtctcatg agcggataca tatttgaatg
7921 tatttagaaa aataaacaaa taggggttcc gcgcacattt ccccgaaaag tgccac
//

pB\(b2-mCherry)-attB-dsRFP-355beta2-GFP

```
LOCUS       pB\(b2-mCherry)-attB-dsRFP-355beta2-GFP        7976 bp    DNA     circular UNA 23-MAR-
2017
DEFINITION  Mars-355.
ACCESSION   urn.local...11-781tst0
VERSION     urn.local...11-781tst0
KEYWORDS    .
SOURCE
  ORGANISM  .
FEATURES             Location/Qualifiers
     rep_origin      21..327
                     /label="f1_origin"
     CDS             complement(471..614)
                     /modified_by="User"
                     /label="lacZ_a"
     promoter        600..616
                     /label="M13_forward20_primer"
     promoter        626..644
                     /label="T7_promoter"
     misc_feature    join(677..>711,<5296..5321)
                     /created_by="User"
                     /label="MCS"
     ligation        708..711
                     /label="Ligation"
     ligation        712..715
                     /label="Ligation"
     misc_feature    716..2513
                     /Source="referenceToDocument: urn:local:.:14s-6crwd4p"
                     /label="Digestion of pUC-mCherry - Fragment 3 extraction
                     (concatenated sequence 2)"
                     /note="Geneious type: Concatenated sequence"
     promoter        725..1223
                     /vntifkey=29
                     /label="b2\promoter"
     CDS             1230..1940
                     /vntifkey=4
                     /label="mCherry"
     terminator      1949..2435
                     /vntifkey=21
                     /modified_by="User"
                     /label="b2\3'UTR"
     misc_recomb     2458..2513
                     /vntifkey=86
                     /label="attB"
     ligation        2514..2517
                     /label="Ligation"
     misc_feature    2517^2518
                     /Original_Bases="A"
                     /label="A"
                     /note="Geneious type: Editing History Deletion"
     misc_feature    2518..5289
                     /Source="referenceToDocument: urn:local:.:14s-6crwd4p"
                     /label="Digestion of pUC-RFP-355b2GFP - Fragment 1
                     extraction (concatenated sequence 3)"
                     /note="Geneious type: Concatenated sequence"
     primer_bind     2518..2538
                     /Sequence="ATGGTGCGCTCCTCCAAGAAC"
                     /Hairpin_Tm="40.3"
                     /Tm="63.0"
                     /Self_Dimer_Tm="19.3"
                     /%GC="57.1"
                     /created_by="primer3"
                     /label="aaa"
     CDS             2518..3195
                     /vntifkey=4
                     /label="DsRed\(RFP)"
     terminator      3206..3439
                     /vntifkey=43
                     /label="SV40"
     promoter        3469..4067
                     /vntifkey=21
                     /modified_by="User"
                     /label="b2promoter"
     misc_feature    3484..3487
                     /vntifkey=21
                     /label="GATA"
     misc_feature    3570..3619
                     /created_by="User"
                     /label="RE 0.41"
```

```
     misc_feature    3578..3627
                     /created_by="User"
                     /label="RE 0.49"
     Synthetic       3630..3729
                     /created_by="User"
                     /label="spacer"
     misc_feature    3730..3776
                     /created_by="User"
                     /label="RE 0.36"
     misc_feature    3852..3855
                     /vntifkey=21
                     /label="GATA"
     misc_feature    3962..3965
                     /vntifkey=21
                     /label="GATA"
     misc_feature    4059..4062
                     /vntifkey=21
                     /label="GATA"
     CDS             4071..>4787
                     /vntifkey=4
                     /modified_by="User"
                     /label="eGFP"
     terminator      4790..5285
                     /vntifkey=21
                     /modified_by="User"
                     /label="b2terminator"
     misc_feature    <5290..5295
                     /Source="referenceToDocument: urn:local:.:1o-781tstz"
                     /label="Annealed link-E2-F2 extraction (concatenated
                     sequence 2)"
                     /note="Geneious type: Concatenated sequence"
     ligation        5290..5293
                     /label="Ligation"
     misc_feature    5294..711
                     /label="Digestion of pBC-Linker-E2-F2 - Fragment 2
                     (concatenated sequence 1)"
                     /note="Geneious type: Concatenated sequence"
     ligation        5296..5299
                     /label="Ligation"
     promoter        complement(5348..5367)
                     /label="T3_promoter"
     promoter        complement(5384..5402)
                     /label="M13_reverse_primer"
     misc_feature    complement(5401..5423)
                     /label="M13_pUC_rev_primer"
     promoter        complement(5437..5466)
                     /label="lac_promoter"
     primer_bind     complement(5700..5723)
                     /Sequence="CCTGCGTTATCCCCTGATTCTGTG"
                     /Hairpin_Tm="32.2"
                     /Tm="63.3"
                     /Self_Dimer_Tm="None"
                     /%GC="54.2"
                     /created_by="primer3"
                     /label="Bluescript_r2"
     primer_bind     5733..5754
                     /Sequence="GTGAGCAAAAGGCCAGCAAAAG"
                     /Hairpin_Tm="46.5"
                     /Tm="61.4"
                     /Self_Dimer_Tm="11.3"
                     /%GC="50.0"
                     /created_by="primer3"
                     /Extension="TTAATTAA"
                     /modified_by="User"
                     /label="pBC_Pac"
     primer_bind     5754..5774
                     /Sequence="GGCCAGGAACCGTAAAAAGGC"
                     /Hairpin_Tm="None"
                     /Tm="62.1"
                     /Self_Dimer_Tm="0.9"
                     /%GC="57.1"
                     /created_by="primer3"
                     /Extension="TTAATTAA"
                     /modified_by="User"
                     /label="BluescriptPacI_f"
     rep_origin      complement(5775..6394)
                     /label="pBR322_origin"
     gene            6701..7360
                     /gene="CAT/CamR"
                     /label="CAT/CamR"
```

```
     CDS             6701..7360
                     /translation="MEKKITGYTTVDISQWHRKEHFEAFQSVAQCTYNQTVQLDITAF
                     LKTVKKNKHKFYPAFIHILARLMNAHPELRMAMKDGELVIWDSVHPCYTVFHEQTETF
                     SSLWSEYHDDFRQFLHIYSQDVACYGENLAYFPKGFIENMFFVSANPWVSFTSFDLNV
                     ANMDNFFAPVFTMGKYYTQGDKVLMPLAIQVHHAVCDGFHVGRMLNELQQYCDEWQGG
                     A*"
                     /label="ORF frame 1"
     gene            complement(7647..7847)
                     /gene="Ampicillin (860 - 660)"
                     /translation="MEKKITGYTTVDISQWHRKEHFEAFQSVAQCTYNQTVQLDITAF
                     LKTVKKNKHKFYPAFIHILARLMNAHPELRMAMKDGELVIWDSVHPCYTVFHEQTETF
                     SSLWSEYHDDFRQFLHIYSQDVACYGENLAYFPKGFIENMFFVSANPWVSFTSFDLNV
                     ANMDNFFAPVFTMGKYYTQGDKVLMPLAIQVHHAVCDGFHVGRMLNELQQYCDEWQGG
                     A*"
                     /label="Ampicillin (860 - 660)"
     promoter        complement(7890..7918)
                     /translation="MEKKITGYTTVDISQWHRKEHFEAFQSVAQCTYNQTVQLDITAF
                     LKTVKKNKHKFYPAFIHILARLMNAHPELRMAMKDGELVIWDSVHPCYTVFHEQTETF
                     SSLWSEYHDDFRQFLHIYSQDVACYGENLAYFPKGFIENMFFVSANPWVSFTSFDLNV
                     ANMDNFFAPVFTMGKYYTQGDKVLMPLAIQVHHAVCDGFHVGRMLNELQQYCDEWQGG
                     A*"
                     /label="AmpR_promoter"
ORIGIN
        1 ctgacgcgcc ctgtagcggc gcattaagcg cggcgggtgt ggtggttacg cgcagcgtga
       61 ccgctacact tgccagcgcc ctagcgcccg ctcctttcgc tttcttccct tcctttctcg
      121 ccacgttcgc cggctttccc cgtcaagctc taaatcgggg gctcccttta gggttccgat
      181 ttagtgcttt acggcacctc gaccccaaaa aacttgatta gggtgatggt tcacgtagtg
      241 ggccatcgcc ctgatagacg gtttttcgcc ctttgacgtt ggagtccacg ttctttaata
      301 gtggactctt gttccaaact ggaacaacac tcaaccctat ctcggtctat tcttttgatt
      361 tataagggat tttgccgatt tcggcctatt ggttaaaaaa tgagctgatt taacaaaaat
      421 ttaacgcgaa ttttaacaaa atattaacgc ttacaatttc cattcgccat tcaggctgcg
      481 caactgttgg gaagggcgat cggtgcgggc ctcttcgcta ttacgccagc tggcgaaagg
      541 gggatgtgct gcaaggcgat taagttgggt aacgccaggg ttttcccagt cacgacgttg
      601 taaaacgacg gccagtgagc gcgcgtaata cgactcacta tagggcgaat tggagctcca
      661 ccgcggtggc ggccgctcta gaactagtgg atcccccggg ctgcaggaat tatccccgac
      721 cggtctagcg ttcataattg atatagtttt gtaaatgaca tgacagtttt ttattttttt
      781 tttatccata attacgaatt gaacaactct acacacatat taattgcaag aacttatgct
      841 acataatatg gaggaaagtg gatgcatcat cccatccaag aagacatacg aatttatttg
      901 tggcatcgca atcggccgaa ccagcaaaag atgatcatag tagtcatatg agccgtacgt
      961 gccggatcat ttcgtgcaga accttcagag acgttggtcg acagattgat agaaactgtg
     1021 tagttagtcc attcacaaag ttgttccatt agggacaaaa gaaaaaaacg gcttaaacta
     1081 gaaatttgtg tacccagtag gaatcgcgta ttcggccgat agaggtcctt ccgtaagtat
     1141 tcccagcgct tagagagcaa cgctcgtgcg ttccaaaatc cgctaaatat caaacggctt
     1201 tcacagtttc gaaagatatc aagctcgaga tggtgagcaa gggcgaggag gataacatgg
     1261 ccatcatcaa ggagttcatg cgcttcaagg tgcacatgga gggctccgtg aacggccacg
     1321 agttcgagat cgagggcgag ggcgagggcc gcccctacga gggcacccag accgccaagc
     1381 tgaaggtgac caagggtggc cccctgccct tcgcctggga catcctgtcc cctcagttca
     1441 tgtacggctc caaggcctac gtgaagcacc ccgccgacat ccccgactac ttgaagctgt
     1501 ccttccccga gggcttcaag tgggagcgcg tgatgaactt cgaggacggc ggcgtggtga
     1561 ccgtgaccca ggactcctcc ctgcaggacg gcgagttcat ctacaaggtg aagctgcgcg
     1621 gcaccaactt ccccctccgac ggccccgtaa tgcagaagaa gaccatgggc tgggaggcct
     1681 cctccgagcg gatgtacccc gaggacggcg ccctgaaggg cgagatcaag cagaggctga
     1741 agctgaagga cggcggccac tacgacgctg aggtcaagac cacctacaag gccaagaagc
     1801 ccgtgcagct gcccggcgcc tacaacgtca acatcaagtt ggacatcacc tcccacaacg
     1861 aggactacac catcgtggaa cagtacgaac gcgccgaggg ccgccactcc accggcggca
     1921 tggacgagct gtacaagtaa ttaattaact aaagctaaat tgaacaccct aaattatgtg
     1981 taaaatttct gctaagcagc ggtgttgggg tcaataaaaa tgtttttttc cactctattc
     2041 gcttcgtttt tgttgccatt tctcagtttt tgcttcgtac tcatgtgtaa ggattagtgc
     2101 agtgatggga agtagctccg aagttttctg gaatcgtttc cggatagtag gttcggtatt
     2161 agtttccgga atcggctccg gaattggttc cggaattgat tccgggatca gaattggctc
     2221 aaaattctca tggagattcc cagagtgatt tcgcttctga aacttcgtat ttaattcaag
     2281 aattgatccc cattctggag ctaattccaa ttctggagtc aattctgatt ctgttacccg
     2341 aacaaattgc gattcccagg tcaatgcccg ttccggggtn gattctgatt ccggagttgg
     2401 aatcagctcc ggaattggaa tcggtcctta aatcgggcgc gccacatgtg gcccagctgc
     2461 gggtgccagg gcgtgccctt gggctccccg ggcgcgtact ccacctcacc catcaacatg
     2521 gtgcgctcct ccaagaacgt catcaaggag ttcatgcgct tcaaggtgcg catggagggc
     2581 accgtgaacg gccacgagtt cgagatcgag ggcgagggcg agggccgccc ctacgagggc
     2641 cacaacaccg tgaagctgaa ggtgaccaag ggcggccccc tgcccttcgc ctgggacatc
     2701 ctgtcccccc agttccagta cggctccaag gtgtacgtga agcaccccgc cgacatcccc
     2761 gactacaaga agctgtcctt ccccgagggc ttcaagtggg agcgcgtgat gaacttcgag
     2821 gacggcggcg tggtgaccgt gacccaggac tcctccctgc aggacggctg cttcatctac
     2881 aaggtgaagt tcatcggcgt gaacttcccc tccgacggcc ccgtaatgca gaagaagacc
     2941 atgggctggg aggcctccac cgagcgcctg taccccccgcg acggcgtgct gaagggcgag
     3001 atccacaagg ccctgaagct gaaggacggc ggccactacc tggtggagtt caagtccatc
     3061 tacatggcca agaagcccgt gcagctgccc ggctactact acgtggactc caagctggac
     3121 atcacctccc acaacgagga ctacaccatc gtggagcagt acgagcgcac cgagggccgc
     3181 caccacctgt tcctgtagcg gccgcgactc tagatcataa tcagccatac cacatttgta
     3241 gaggttttac ttgctttaaa aaacctccca cacctccccc tgaacctgaa acataaaatg
     3301 aatgcaattg ttgttgttaa cttgtttatt gcagcttata atggttacaa ataaagcaat
     3361 agcatcacaa atttcacaaa taaagcattt ttttcactgc attctagttg tggtttgtcc
```

```
3421 aaactcatca atgtatctta aagcttatcg atacgcgtac ggcgcgccct agcgttcata
3481 attgatatag ttttgtaaat gacatgacag tttttattt ttttttatc cataattacg
3541 aattgaacaa ctctacacac atattaattg caagaactta tgctacataa tatggaggaa
3601 agtggatgca tcatcccatc caagaagacg actcggaccc gagttggcca acgacccacg
3661 ggcggagtta gggcggatgg tgagaagtgc gcgtctcgtt cccgcagctc gccagcactc
3721 tcagactcaa tacgaattta tttgtggcat cgcaatcggc cgaaccagca aaagatgatc
3781 atagtagtca tatgagccgt acgtgccgga tcatttcgtg cagaaccttc agagacgttg
3841 gtcgacagat tgatagaaac tgtgtagtta gtccattcac aaagttgttc cattagggac
3901 aaaagaaaaa aacggcttaa actagaaatt tgtgtaccca gtaggaatcg cgtattcggc
3961 cgatagaggt ccttccgtaa gtattcccag cgcttagaga gcaacgctcg tgcgttccaa
4021 aatccgctaa atatcaaacg gctttcacag tttcgaaaga tatcaagctt atggtgagca
4081 agggcgagga gctgttcacc ggggtggtgc ccatcctggt cgagctggac ggcgacgtaa
4141 acggccacaa gttcagcgtg tccggcgagg gcgagggcga tgccacctac ggcaagctga
4201 ccctgaagtt catctgcacc accggcaagc tgcccgtgcc ctggcccacc ctcgtgacca
4261 ccctgaccta cggcgtgcag tgcttcagcc gctaccccga ccacatgaag cagcacgact
4321 tcttcaagtc cgccatgccc gaaggctacg tccaggagcg caccatcttc ttcaaggacg
4381 acggcaacta caagacccgc gccgaggtga agttcgaggg cgacaccctg gtgaaccgca
4441 tcgagctgaa gggcatcgac ttcaaggagg acggcaacat cctggggcac aagctggagt
4501 acaactacaa cagccacaac gtctatatca tggccgacaa gcagaagaac ggcatcaagg
4561 tgaacttcaa gatccgccac aacatcgagg acggcagcgt gcagctcgcc gaccactacc
4621 agcagaacac ccccatcggc gacggccccg tgctgctgcc cgacaaccac tacctgagca
4681 cccagtccgc cctgagcaaa gaccccaacg agaagcgcga tcacatggtc ctgctggagt
4741 tcgtgaccgc cgccgggatc actctcggca tggacgagct gtacaagtaa gcttaactaa
4801 agctaaattg aacaccctaa attatgtgta aaatttctgc taagcagcgg tgttggggtc
4861 aataaaaatg tttttttcca ctctattcgc ttcgtttttg ttgccatttc tcagtttttg
4921 cttcgtactc atgtgtaagg attagtgcag tgatgggaag tagctccgaa gttttctgga
4981 atcgtttccg gatagtaggt tcggtattag tttccggaat cggctccgga attggttccg
5041 gaattgattc cgggatcaga attggctcaa aattctcatg gagattccca gagtgatttc
5101 gcttctgaaa cttcgtattt aattcaagaa ttgatcccca ttctggagct aattccaatt
5161 ctggagtcaa ttctgattct gttacccgaa caaattgcga ttcccaggtc aatgcccatt
5221 ccggggacga ttctgattcc ggagttggaa tcagctccgg aattggaatc ggtccttaaa
5281 tcgggcgcgc ctaggagctt atcgataccg tcgacctcga ggggggcccc ggtacccagc
5341 ttttgttccc tttagtgagg gttaattgcg cgcttggcgt aatcatggtc atagctgttt
5401 cctgtgtgaa attgttatcc gctcacaatt ccacacaaca tacgagccgg aagcataaag
5461 tgtaaagcct ggggtgccta atgagtgagc taactcacat taattgcgtt gcgctcactg
5521 cccgctttcc agtcgggaaa cctgtcgtgc cagctgcatt aatgaatcgg ccaacgcgcg
5581 gggagaggcg gtttgcgtat tgggcgctct tccgcttcct cgctcactga ctcgctgcgc
5641 tcggtcgttc ggctgcggcg agcggtatca gctcactcaa aggcggtaat acggttatcc
5701 acagaatcag gggataacgc aggaaagaac atgtgagcaa aaggccagca aaaggccagg
5761 aaccgtaaaa aggccgcgtt gctggcgttt ttccataggc tccgcccccc tgacgagcat
5821 cacaaaaatc gacgctcaag tcagaggtgg cgaaacccga caggactata aagataccag
5881 gcgtttcccc ctggaagctc cctcgtgcgc tctcctgttc cgaccctgcc gcttaccgga
5941 tacctgtccg cctttctccc ttcgggaagc gtggcgcttt ctcatagctc acgctgtagg
6001 tatctcagtt cggtgtaggt cgttcgctcc aagctgggct gtgtgcacga accccccgtt
6061 cagcccgacc gctgcgcctt atccggtaac tatcgtcttg agtccaaccc ggtaagacac
6121 gacttatcgc cactggcagc agccactggt aacaggatta gcagagcgag gtatgtaggc
6181 ggtgctacag agttcttgaa gtggtggcct aactacggct acactagaag gacagtattt
6241 ggtatctgcg ctctgctgaa gccagttacc ttcggaaaaa gagttggtag ctcttgatcc
6301 ggcaaacaaa ccaccgctgg tagcggtggt ttttttgttt gcaagcagca gattacgcgc
6361 agaaaaaaag gatctcaaga agatcctttg atcttttcta cggggtctga cgctcagtgg
6421 aacgaaaact cacgttaagg gattttggtc atgagattat caaaaaggat cttcacctag
6481 atccttttcg accgaataaa tacctgtgac ggaagatcac ttcgcagaat aaataaatcc
6541 tggtgtccct gttgataccg ggaagccctg ggccaacttt tggcgaaaat gagacgttga
6601 tcggcacgta agaggttcca actttcacca taatgaaata agatcactac cgggcgtatt
6661 ttttgagttg tcgagatttt caggagctaa ggaagctaaa atggagaaaa aaatcactgg
6721 atataccacc gttgatatat cccaatggca tcgtaaagaa cattttgagg catttcagtc
6781 agttgctcaa tgtacctata accagaccgt tcagctggat attacggcct ttttaaagac
6841 cgtaaagaaa aataagcaca agttttatcc ggcctttatt cacattcttg cccgcctgat
6901 gaatgctcat ccggaattac gtatggcaat gaaagacggt gagctggtga tatgggatag
6961 tgttcaccct tgttacaccg ttttccatga gcaaactgaa acgttttcat cgctctggag
7021 tgaataccac gacgatttcc ggcagtttct acacatatat tcgcaagatg tggcgtgtta
7081 cggtgaaaac ctggcctatt tccctaaagg gtttattgag aatatgtttt tcgtctcagc
7141 caatccctgg gtgagtttca ccagttttga tttaaacgtg gccaatatgg acaacttctt
7201 cgcccccgtt ttcaccatgg gcaaatatta tacgcaaggc gacaaggtgc tgatgccgct
7261 ggcgattcag gttcatcatg ccgtttgtga tggcttccat gtcggcagaa tgcttaatga
7321 attacaacag tactgcgatg agtggcaggg cggggcgtaa tttttttaag gcagttattg
7381 gtgcccttaa acgcctggtt gctacgcctg aataagtgat aataagcgga tgaatggcag
7441 aaattcgaaa gcaaattcga cccggtcgtc ggttcagggc agggtcgtta aatagccgct
7501 tatgtctatt gctggtttac cggtttattg actaccggaa gcagtgtgac cgtgtgcttc
7561 tcaaatgcct gaggccagtt tgctcaggct ctccccgtgg aggtaataat tgacgatatg
7621 atcctttttt tctgatcaaa agtgctcatc attggaaaac gttcttcggg gcgaaaactc
7681 tcaaggatct taccgctgtt gagatccagt tcgatgtaac ccactcgtgc acccaactga
7741 tcttcagcat cttttacttt caccagcgtt tctgggtgag caaaaacagg aaggcaaaat
7801 gccgcaaaaa agggaataag ggcgacacgg aaatgttgaa tactcatact cttccttttt
7861 caatattatt gaagcattta tcaagggtta ttgtctcatg agcggataca tatttgaatg
7921 tatttagaaa aataaacaaa taggggttcc gcgcacattt ccccgaaaag tgccac
//
```

pB\(b2-mCherry)-attB-dsRFP-WTbeta2-GFP_v2

```
LOCUS       pB\(b2-mCherry)-attB-dsRFP-WTbeta2-GFP_v2        7876 bp    DNA     circular UNA 17-AUG-
2016
DEFINITION  Extra A removed.
ACCESSION   urn.local...13-781tst1
VERSION     urn.local...13-781tst1
KEYWORDS    .
SOURCE
  ORGANISM  .
FEATURES             Location/Qualifiers
     misc_feature    1..>711
                     /Source="referenceToDocument: urn:local::dh-656isi9"
                     /label="Digestion of pBC-Linker - Fragment 2 extraction -
                     Digestion of pBC KS(-) (modified) (concatenated sequence
                     1) (concatenated sequence 1)"
                     /note="Geneious type: Concatenated sequence"
     rep_origin      21..327
                     /label="f1_origin"
     CDS             complement(471..614)
                     /modified_by="User"
                     /label="lacZ_a"
     promoter        600..616
                     /label="M13_forward20_primer"
     promoter        626..644
                     /label="T7_promoter"
     misc_feature    join(677..>711,<5196..5221)
                     /created_by="User"
                     /label="MCS"
     misc_feature    711^712
                     /Original_Bases="CGAT"
                     /label="CGAT"
                     /note="Geneious type: Editing History Deletion"
     ligation        712..715
                     /label="Ligation"
     promoter        725..1223
                     /vntifkey=29
                     /label="b2\promoter"
     CDS             1230..1940
                     /vntifkey=4
                     /label="mCherry"
     misc_feature    1949..2435
                     /vntifkey=21
                     /label="b2\3'UTR"
     misc_recomb     2458..2513
                     /vntifkey=86
                     /label="attB"
     ligation        2514..2517
                     /label="Ligation"
     misc_feature    2517^2518
                     /Original_Bases="A"
                     /label="A"
                     /note="Geneious type: Editing History Deletion"
     CDS             2518..3195
                     /vntifkey=4
                     /label="DsRed\(RFP)"
     terminator      3206..3439
                     /vntifkey=43
                     /label="SV40"
     promoter        3469..3967
                     /vntifkey=21
                     /modified_by="User"
                     /label="b2promoter"
     misc_feature    3570..3619
                     /created_by="User"
                     /label="RE 0.41"
     misc_feature    3578..3627
                     /created_by="User"
                     /label="RE 0.49"
     misc_feature    3627..3676
                     /created_by="User"
                     /label="RE 0.36"
     CDS             3971..>4687
                     /vntifkey=4
                     /modified_by="User"
                     /label="eGFP"
     terminator      4690..5185
                     /vntifkey=21
                     /modified_by="User"
                     /label="b2terminator"
```

```
     misc_feature    <5190..5195
                     /Source="referenceToDocument: urn:local:.:1s-781tsu6"
                     /label="Linker E-F (concatenated sequence 2)"
                     /note="Geneious type: Concatenated sequence"
     ligation        5190..5193
                     /label="Ligation"
     misc_feature    5196^5197
                     /Original_Bases="TCAA"
                     /label="TCAA"
                     /note="Geneious type: Editing History Deletion"
     promoter        complement(5248..5267)
                     /label="T3_promoter"
     promoter        complement(5284..5302)
                     /label="M13_reverse_primer"
     misc_feature    complement(5301..5323)
                     /label="M13_pUC_rev_primer"
     promoter        complement(5337..5366)
                     /label="lac_promoter"
     rep_origin      complement(5675..6294)
                     /label="pBR322_origin"
     gene            6601..7260
                     /gene="CAT/CamR"
                     /label="CAT/CamR"
     CDS             6601..7260
                     /translation="MEKKITGYTTVDISQWHRKEHFEAFQSVAQCTYNQTVQLDITAF
                     LKTVKKNKHKFYPAFIHILARLMNAHPELRMAMKDGELVIWDSVHPCYTVFHEQTETF
                     SSLWSEYHDDFRQFLHIYSQDVACYGENLAYFPKGFIENMFFVSANPWVSFTSFDLNV
                     ANMDNFFAPVFTMGKYYTQGDKVLMPLAIQVHHAVCDGFHVGRMLNELQQYCDEWQGG
                     A*"
                     /label="ORF frame 1"
     gene            complement(7547..7747)
                     /gene="Ampicillin (860 - 660)"
                     /translation="MEKKITGYTTVDISQWHRKEHFEAFQSVAQCTYNQTVQLDITAF
                     LKTVKKNKHKFYPAFIHILARLMNAHPELRMAMKDGELVIWDSVHPCYTVFHEQTETF
                     SSLWSEYHDDFRQFLHIYSQDVACYGENLAYFPKGFIENMFFVSANPWVSFTSFDLNV
                     ANMDNFFAPVFTMGKYYTQGDKVLMPLAIQVHHAVCDGFHVGRMLNELQQYCDEWQGG
                     A*"
                     /label="Ampicillin (860 - 660)"
     promoter        complement(7790..7818)
                     /translation="MEKKITGYTTVDISQWHRKEHFEAFQSVAQCTYNQTVQLDITAF
                     LKTVKKNKHKFYPAFIHILARLMNAHPELRMAMKDGELVIWDSVHPCYTVFHEQTETF
                     SSLWSEYHDDFRQFLHIYSQDVACYGENLAYFPKGFIENMFFVSANPWVSFTSFDLNV
                     ANMDNFFAPVFTMGKYYTQGDKVLMPLAIQVHHAVCDGFHVGRMLNELQQYCDEWQGG
                     A*"
                     /label="AmpR_promoter"
ORIGIN
        1 ctgacgcgcc ctgtagcggc gcattaagcg cggcgggtgt ggtggttacg cgcagcgtga
       61 ccgctacact tgccagcgcc ctagcgcccg ctcctttcgc tttcttccct tcctttctcg
      121 ccacgttcgc cggctttccc cgtcaagctc taaatcgggg gctcccttta gggttccgat
      181 ttagtgcttt acggcacctc gaccccaaaa aacttgatta gggtgatggt tcacgtagtg
      241 ggccatcgcc ctgatagacg gtttttcgcc ctttgacgtt ggagtccacg ttctttaata
      301 gtggactctt gttccaaact ggaacaacac tcaaccctat ctcggtctat tcttttgatt
      361 tataagggat tttgccgatt tcggcctatt ggttaaaaaa tgagctgatt taacaaaaat
      421 ttaacgcgaa ttttaacaaa atattaacgc ttacaatttc cattcgccat tcaggctgcg
      481 caactgttgg gaagggcgat cggtgcgggc ctcttcgcta ttacgccagc tggcgaaagg
      541 gggatgtgct gcaaggcgat taagttgggt aacgccaggg ttttcccagt cacgacgttg
      601 taaaacgacg gccagtgagc gcgcgtaata cgactcacta tagggcgaat tggagctcca
      661 ccgcggtggc ggccgctcta gaactagtgg atcccccggg ctgcaggaat tatccccgac
      721 cggtctagcg ttcataattg atatagtttt gtaaatgaca tgacagtttt ttattttttt
      781 tttatccata attacgaatt gaacaactct acacacatat taattgcaag aacttatgct
      841 acataatatg gaggaaagtg gatgcatcat cccatccaag aagacatacg aatttatttg
      901 tggcatcgca atcggccgaa ccagcaaaag atgatcatag tagtcatatg agccgtacgt
      961 gccggatcat ttcgtgcaga accttcagag acgttggtcg acagattgat agaaactgtg
     1021 tagttagtcc attcacaaag ttgttccatt agggacaaaa gaaaaaaacg gcttaaacta
     1081 gaaatttgtg tacccagtag gaatcgcgta ttcggccgat agaggtcctt ccgtaagtat
     1141 tcccagcgct tagagagcaa cgctcgtgcg ttccaaaatc cgctaaatat caaacggctt
     1201 tcacagtttc gaaagatatc aagctcgaga tggtgagcaa gggcgaggag gataacatgg
     1261 ccatcatcaa ggagttcatg cgcttcaagg tgcacatgga gggctccgtg aacggccacg
     1321 agttcgagat cgagggcgag ggcgagggcc gcccctacga gggcacccag accgccaagc
     1381 tgaaggtgac caagggtggc cccctgccct tcgcctggga catcctgtcc cctcagttca
     1441 tgtacggctc caaggcctac gtgaagcacc ccgccgacat ccccgactac ttgaagctgt
     1501 ccttccccga gggcttcaag tgggagcgcg tgatgaactt cgaggacggc ggcgtggtga
     1561 ccgtgaccca ggactcctcc ctgcaggacg gcgagttcat ctacaaggtg aagctgcgcg
     1621 gcaccaactt cccctccgac ggccccgtaa tgcagaagaa gaccatgggc tgggaggcct
     1681 cctccgagcg gatgtacccc gaggacggcg ccctgaaggg cgagatcaag cagaggctga
     1741 agctgaagga cggcggccac tacgacgctg aggtcaagac cacctacaag gccaagaagc
     1801 ccgtgcagct gcccggcgcc tacaacgtca acatcaagtt ggacatcacc tcccacaacg
     1861 aggactacac catcgtggaa cagtacgaac gcgccgaggg ccgccactcc accggcggca
     1921 tggacgagct gtacaagtaa ttaattaact aaagctaaat tgaacaccct aaattatgtg
     1981 taaaatttct gctaagcagc ggtgttgggg tcaataaaaa tgtttttttc cactctattc
```

2041 gcttcgtttt tgttgccatt tctcagtttt tgcttcgtac tcatgtgtaa ggattagtgc
2101 agtgatggga agtagctccg aagttttctg gaatcgtttc cggatagtag gttcggtatt
2161 agtttccgga atcggctccg gaattggttc cggaattgat tccgggatca gaattggctc
2221 aaaattctca tggagattcc cagagtgatt tcgcttctga aacttcgtat ttaattcaag
2281 aattgatccc cattctggag ctaattccaa ttctggagtc aattctgatt ctgttacccg
2341 aacaaattgc gattcccagg tcaatgcccg ttccggggtn gattctgatt ccggagttgg
2401 aatcagctcc ggaattggaa tcggtcctta aatcgggcgc gccacatgtg gcccagctgc
2461 gggtgccagg gcgtgccctt gggctccccg ggcgcgtact ccacctcacc catcaacatg
2521 gtgcgctcct ccaagaacgt catcaaggag ttcatgcgct tcaaggtgcg catggagggc
2581 accgtgaacg gccacgagtt cgagatcgag ggcgagggcg agggccgccc ctacgagggc
2641 cacaacaccg tgaagctgaa ggtgaccaag ggcggccccc tgcccttcgc ctgggacatc
2701 ctgtcccccc agttccagta cggctccaag gtgtacgtga agcaccccgc cgacatcccc
2761 gactacaaga agctgtcctt ccccgagggc ttcaagtggg agcgcgtgat gaacttcgag
2821 gacggcggcg tggtgaccgt gacccaggac tcctccctgc aggacggctg cttcatctac
2881 aaggtgaagt tcatcggcgt gaacttcccc tccgacggcc ccgtaatgca gaagaagacc
2941 atgggctggg aggcctccac cgagcgcctg tacccccgcg acggcgtgct gaagggcgag
3001 atccacaagg ccctgaagct gaaggacggc ggccactacc tggtggagtt caagtccatc
3061 tacatggcca agaagcccgt gcagctgccc ggctactact acgtggactc caagctggac
3121 atcacctccc acaacgagga ctacaccatc gtggagcagt acgagcgcac cgagggccgc
3181 caccacctgt tcctgtagcg gccgcgactc tagatcataa tcagccatac cacatttgta
3241 gaggttttac ttgctttaaa aaacctccca cacctccccc tgaacctgaa acataaaatg
3301 aatgcaattg ttgttgttaa cttgtttatt gcagcttata atggttacaa ataaagcaat
3361 agcatcacaa atttcacaaa taaagcattt ttttcactgc attctagttg tggtttgtcc
3421 aaactcatca atgtatctta aagcttatcg atacgcgtac ggcgcgccct agcgttcata
3481 attgatatag ttttgtaaat gacatgacag tttttttattt tttttttatc cataattacg
3541 aattgaacaa ctctacacac atattaattg caagaactta tgctacataa tatggaggaa
3601 agtggatgca tcatcccatc caagaagaca tacgaattta tttgtggcat cgcaatcggc
3661 cgaaccagca aaagatgatc atagtagtca tatgagccgt acgtgccgga tcatttcgtg
3721 cagaaccttc agagacgttg gtcgacagat tgatagaaac tgtgtagtta gtccattcac
3781 aaagttgttc cattagggac aaaagaaaaa aacggcttaa actagaaatt tgtgtaccca
3841 gtaggaatcg cgtattcggc cgatagaggt ccttccgtaa gtattcccag cgcttagaga
3901 gcaacgctcg tgcgttccaa aatccgctaa atatcaaacg gctttcacag tttcgaaaga
3961 tatcaagctt atggtgagca agggcgagga gctgttcacc ggggtggtgc ccatcctggt
4021 cgagctggac ggcgacgtaa acggccacaa gttcagcgtg tccggcgagg gcgagggcga
4081 tgccacctac ggcaagctga ccctgaagtt catctgcacc accggcaagc tgcccgtgcc
4141 ctggcccacc ctcgtgacca ccctgaccta cggcgtgcag tgcttcagcc gctaccccga
4201 ccacatgaag cagcacgact tcttcaagtc cgccatgccc gaaggctacg tccaggagcg
4261 caccatcttc ttcaaggacg acggcaacta caagacccgc gccgaggtga agttcgaggg
4321 cgacaccctg gtgaaccgca tcgagctgaa gggcatcgac ttcaaggagg acggcaacat
4381 cctggggcac aagctggagt acaactacaa cagccacaac gtctatatca tggccgacaa
4441 gcagaagaac ggcatcaagg tgaacttcaa gatccgccac aacatcgagg acggcagcgt
4501 gcagctcgcc gaccactacc agcagaacac ccccatcggc gacggccccg tgctgctgcc
4561 cgacaaccac tacctgagca cccagtccgc cctgagcaaa gaccccaacg agaagcgcga
4621 tcacatggtc ctgctggagt tcgtgaccgc cgccgggatc actctcggca tggacgagct
4681 gtacaagtaa gcttaactaa agctaaattg aacaccctaa attatgtgta aaatttctgc
4741 taagcagcgg tgttggggtc aataaaaatg tttttttcca ctctattcgc ttcgtttttg
4801 ttgccatttc tcagtttttg cttcgtactc atgtgtaagg attagtgcag tgatgggaag
4861 tagctccgaa gttttctgga atcgtttccg gatagtaggt tcggtattag tttccggaat
4921 cggctccgga attggttccg gaattgattc cgggatcaga attggctcaa aattctcatg
4981 gagattccca gagtgatttc gcttctgaaa cttcgtattt aattcaagaa ttgatcccca
5041 ttctggagct aattccaatt ctggagtcaa ttctgattct gttacccgaa caaattgcga
5101 ttcccaggtc aatgcccatt ccggggacga ttctgattcc ggagttggaa tcagctccgg
5161 aattggaatc ggtccttaaa tcgggcgcgc ctaggagctt atcgataccg tcgacctcga
5221 gggggggccc ggtacccagc ttttgttccc tttagtgagg gttaattgcg cgcttggcgt
5281 aatcatggtc atagctgttt cctgtgtgaa attgttatcc gctcacaatt ccacacaaca
5341 tacgagccgg aagcataaag tgtaaagcct ggggtgccta atgagtgagc taactcacat
5401 taattgcgtt gcgctcactg cccgctttcc agtcgggaaa cctgtcgtgc cagctgcatt
5461 aatgaatcgg ccaacgcgcg gggagaggcg gtttgcgtat tgggcgctct tccgcttcct
5521 cgctcactga ctcgctgcgc tcggtcgttc ggctgcggcg agcggtatca gctcactcaa
5581 aggcggtaat acggttatcc acagaatcag gggataacgc aggaaagaac atgtgagcaa
5641 aaggccagca aaaggccagg aaccgtaaaa aggccgcgtt gctggcgttt ttccataggc
5701 tccgcccccc tgacgagcat cacaaaaatc gacgctcaag tcagaggtgg cgaaacccga
5761 caggactata aagataccag gcgtttcccc ctggaagctc cctcgtgcgc tctcctgttc
5821 cgaccctgcc gcttaccgga tacctgtccg cctttctccc ttcgggaagc gtggcgcttt
5881 ctcatagctc acgctgtagg tatctcagtt cggtgtaggt cgttcgctcc aagctgggct
5941 gtgtgcacga accccccgtt cagcccgacc gctgcgcctt atccggtaac tatcgtcttg
6001 agtccaaccc ggtaagacac gacttatcgc cactggcagc agccactggt aacaggatta
6061 gcagagcgag gtatgtaggc ggtgctacag agttcttgaa gtggtggcct aactacggct
6121 acactagaag gacagtattt ggtatctgcg ctctgctgaa gccagttacc ttcggaaaaa
6181 gagttggtag ctcttgatcc ggcaaacaaa ccaccgctgg tagcggtggt ttttttgttt
6241 gcaagcagca gattacgcgc agaaaaaaag gatctcaaga agatcctttg atcttttcta
6301 cggggtctga cgctcagtgg aacgaaaact cacgttaagg gattttggtc atgagattat
6361 caaaaaggat cttcacctag atccttttcg accgaataaa tacctgtgac ggaagatcac
6421 ttcgcagaat aaataaatcc tggtgtccct gttgataccg ggaagccctg ggccaacttt
6481 tggcgaaaat gagacgttga tcggcacgta agaggttcca actttcacca taatgaaata
6541 agatcactac cgggcgtatt ttttgagttg tcgagatttt caggagctaa ggaagctaaa
6601 atggagaaaa aaatcactgg atataccacc gttgatatat cccaatggca tcgtaaagaa
6661 cattttgagg catttcagtc agttgctcaa tgtacctata accagaccgt tcagctggat
6721 attacggcct ttttaaagac cgtaaagaaa aataagcaca agttttatcc ggcctttatt
6781 cacattcttg cccgcctgat gaatgctcat ccggaattac gtatggcaat gaaagacggt

```
6841 gagctggtga tatgggatag tgttcaccct tgttacaccg ttttccatga gcaaactgaa
6901 acgttttcat cgctctggag tgaataccac gacgatttcc ggcagtttct acacatatat
6961 tcgcaagatg tggcgtgtta cggtgaaaac ctggcctatt tccctaaagg gtttattgag
7021 aatatgtttt tcgtctcagc caatccctgg gtgagtttca ccagttttga tttaaacgtg
7081 gccaatatgg acaacttctt cgcccccgtt ttcaccatgg gcaaatatta tacgcaaggc
7141 gacaaggtgc tgatgccgct ggcgattcag gttcatcatg ccgtttgtga tggcttccat
7201 gtcggcagaa tgcttaatga attacaacag tactgcgatg agtggcaggg cggggcgtaa
7261 tttttttaag gcagttattg gtgcccttaa acgcctggtt gctacgcctg aataagtgat
7321 aataagcgga tgaatggcag aaattcgaaa gcaaattcga cccggtcgtc ggttcagggc
7381 agggtcgtta aatagccgct tatgtctatt gctggtttac cggtttattg actaccggaa
7441 gcagtgtgac cgtgtgcttc tcaaatgcct gaggccagtt tgctcaggct ctccccgtgg
7501 aggtaataat tgacgatatg atcctttttt tctgatcaaa agtgctcatc attggaaaac
7561 gttcttcggg gcgaaaactc tcaaggatct taccgctgtt gagatccagt tcgatgtaac
7621 ccactcgtgc acccaactga tcttcagcat cttttacttt caccagcgtt tctgggtgag
7681 caaaaacagg aaggcaaaat gccgcaaaaa agggaataag ggcgacacgg aaatgttgaa
7741 tactcatact cttccttttt caatattatt gaagcattta tcaagggtta ttgtctcatg
7801 agcggataca tatttgaatg tatttagaaa aataaacaaa taggggttcc gcgcacattt
7861 ccccgaaaag tgccac
//
```
